# Genome-wide identification and expression profiling analysis of Wnt family genes affecting adipocyte differentiation in cattle

**DOI:** 10.1038/s41598-021-04468-1

**Published:** 2022-01-11

**Authors:** Cuili Pan, Shuzhe Wang, Chaoyun Yang, Chunli Hu, Hui Sheng, Xiaoshu Xue, Honghong Hu, Zhaoxiong Lei, Mengli Yang, Yun Ma

**Affiliations:** 1 School of Agriculture, Ningxia University, Yinchuan, 750021 China; 2 Key Laboratory of Ruminant Molecular and Cellular Breeding, Ningxia Hui Autonomous Region, Ningxia University, Yinchuan, 750021 China; 3 College of Life Sciences, Xinyang Normal University, Xinyang, 464000 Henan China

**Keywords:** Genetics, Animal breeding

## Abstract

The Wnt family features conserved glycoproteins that play roles in tissue regeneration, animal development and cell proliferation and differentiation. For its functional diversity and importance, this family has been studied in several species, but not in the Bovinae. Herein we identified 19 Wnt genes in cattle, and seven other species of Bovinae, and described their corresponding protein properties. Phylogenetic analysis clustered the 149 Wnt proteins in Bovinae, and 38 Wnt proteins from the human and mouse into 12 major clades. Wnt genes from the same subfamilies shared similar protein motif compositions and exon–intron patterns. Chromosomal distribution and collinearity analysis revealed that they were conservative in cattle and five species of Bovinae. RNA-seq data analysis indicated that Wnt genes exhibited tissue-specific expression in cattle. qPCR analysis revealed a unique expression pattern of each gene during bovine adipocytes differentiation. Finally, the comprehensive analysis indicated that *Wnt2B* may regulate adipose differentiation by activating *FZD5*, which is worthy of further study. Our study presents the first genome-wide study of the Wnt gene family in Bovinae, and lays the foundation for further functional characterization of this family in bovine adipocytes differentiation.

## Introduction

Wnt proteins, the initiators of the Wnt signaling pathway, comprise a large family of secreted glycoproteins that are rich in cysteine^1^. The transduction of Wnt signaling mainly includes three pathways: the canonical Wnt (Wnt/β-catenin), non-canonical Wnt/Ca^2+^, and non-canonical planar cell polarity (PCP). To regulate intracellular responses, all three patterns require binding to transmembrane receptor Frizzled (Fzd).

In the canonical Wnt pathway, activation of Fzd leads to the stable accumulation of β-catenin in the cytoplasm and translocation to the nucleus. Then, β-catenin binds to the transcription factor T-cell factor/lymphoid enhancing factor (LEF1/TCF) family. This activates the transcription of target genes that regulate embryo development, tissue regeneration and cell proliferation and differentiation^2,3^.

In the non-canonical Wnt/Ca^2+^ pathway, the activation of Fzd (mainly Fzd2) results in intracellular Ca^2+^ release. This activates Ca^2+^–calmodulin-dependent protein kinase II (CamKII), calnexin (CaN) and protein kinase C (PKC), thus regulating cell adhesion and gene transcription^4^. In the non-canonical Wnt/PCP pathway, Wnt proteins (e.g., Wnt5a and Wnt11) activate Fzd and signals are transmitted from Disheveled (Dvl) to trimeric G proteins. This is followed by the activation of downstream target genes Rho-associated kinase (Rock) and Jun N-terminal serine/threonine kinase (JNK), thereby regulating cytoskeletal actin and cell polarity^5,6^.

Canonical Wnt signaling mainly plays an important role in maintaining precursor adipocytes in an undifferentiated state by inhibiting adipogenesis. In 3T3-L1 preadipocytes, *Wnt1* ectopic expression stabilizes β-catenin and activates TCF-dependent gene transcription, thus blocking adipogenesis^7,8^. Adipogenesis is promoted by Glucagon Like Peptide-1 (*GLP-1*) through the up-regulation of the *Wnt4* and β-catenin^9^. KDM5A interacts with C/EBPβ and cooperatively inhibits the transcription of *Wnt6*, thus leading to the inhibition of the canonical Wnt/β-catenin pathway and promotion of 3T3-L1 preadipocyte differentiation^10^. In human bone marrow stromal (mesenchymal) stem cells (hMSCs), treatment with *Wnt3A* activates the Wnt/β-catenin signaling pathway, thus reducing adipogenesis and increasing osteogenesis^11,12^. *Wnt10B* activates the Wnt signaling cascade and prevents preadipocytes differentiation by inhibiting the expression of *C/EBPα* and *PPARγ*^8,13,14^. In porcine adipose-derived mesenchymal stem cells (AMSCs), *Wnt3A* inhibits the potential of adipogenic differentiation and alters the cell fate from adipocytes to osteoblasts^15^. In murine embryonic mesenchymal cell line C3H10T1/2, the Wnt3-Fz1 chimera is an inhibitor of differentiation into the adipocyte lineage and a potent activator of differentiation into osteoblasts^16^. In addition, the Wnt gene family also functions via the non-canonical Wnt signaling pathway. In hMSCs, the inhibition of *Wnt3A* suppressed the non-canonical Wnt/JNK pathway and enhanced adipocyte differentiation whereas its activation enhanced osteoblast differentiation^11^. In 3T3-L1 preadipocytes, *Wnt4* and *Wnt5A* positively regulated adipogenesis at the initial stage of the differentiation process by activating PKC and calcium/calmodulin-dependent kinase II^17^.

So far, the Wnt family has been extensively studied in some species, e.g., *Drosophila melanogaster*, *Tribolium castaneum*, *Acyrthosiphon pisum*, *Anopheles* *gambiae*, and *Apis mellifera* ^18–22^. Spatiotemporal expression profile revealed that some Wnts might participate in early embryonic development as well as in adult organ/tissue morphogenesis and homeostasis, whereas others may be involved in coping with challenging intertidal environments^20^. Similarly, research on Wnts and Wnt signaling pathway has mainly focused on regulating embryonic development in cattle^23,24^. For instance, Wnt11 activate JNK to improve the competence of the embryo to develop to the blastocyst stage^25^. The expression of *Wnt6* was upregulated in bovine trophectoderm^25^, consistant with the previous study which found that it can promote differentiation of primitive endoderm^26^. Wnt7A inhibited the PCP pathway to improve blastocyst development, without affecting the amount of CTNNB1^27^. These findings stimulated our interest and guided us to explore the evolution of the Wnt gene family in Bovinae and function in adipocyte differentiation.

In the present study we have performed a genome-wide identification and evolutionary analysis of the Wnt gene family in eight species of Bovinae. And the expression profiles in different tissues and stages during adipocytes differentiation were also analyzed in cattle based on transcriptome data and qPCR. Our study provides a basis for understanding the distribution of Wnt genes and will contributes to further elucidate their potential function in adipocytes differentiation.

## Results

### The Wnt gene repertoire in Bovinae

To identify the Wnt family members, 45 verified Wnt amino acid sequences were used as the query terms. These verified sequences were derived from cattle (*Bos taurus,* 7), human (*Homo sapiens,* 19) and mouse (Mus *musculus,* 19). We used these query sequences for genome-wide detection of homologous sequences in *Bos taurus*, *Bos indicus*, *Hybrid-Bos taurus*, *Hybrid-Bos Indicus*, *Bos grunniens*, *Bos mutus*, *Bubalus bubalis* and *Bison bison bison*. In *Bos taurus*, 19 non-redundant Wnt protein sequences were identified (Table 1). Wnt family proteins were also recognized in *Bos indicus* (17), *Hybrid-Bos taurus* (19), *Hybrid-Bos Indicus* (19), *Bos grunniens* (19), *Bos mutus* (19), *Bubalus bubalis* (19) and *Bison bison bison* (18). (Supplementary Info File 1 and 2).

**Table 1:** Characteristics of genome-wide identified Wnt family members in *Bos taurus.*

Gene name	Gene ID	Transcript ID	pI	Mw/kDa	Amino acids	Description
WNT1	ENSBTAG00000015364	ENSBTAT00000020414	9.24	41.04	370	Wnt family member 1
WNT2	ENSBTAG00000008097	ENSBTAT00000010650	9.21	40.64	360	Wnt family member 2
WNT2B	ENSBTAG00000014291	ENSBTAT00000018985	9.32	44.17	394	Wnt family member 2B
WNT3	ENSBTAG00000016012	ENSBTAT00000021312	7.73	39.78	355	Wnt family member 3
WNT3A	ENSBTAG00000039397	ENSBTAT00000056888	8.37	38.42	346	Wnt family member 3A
WNT4	ENSBTAG00000051083	ENSBTAT00000085976	9.11	63.17	585	Wnt family member 4
WNT5A	ENSBTAG00000020221	ENSBTAT00000026930	8.88	42.42	380	Wnt family member 5A
WNT5B	ENSBTAG00000054367	ENSBTAT00000068978	8.99	40.05	358	Wnt family member 5B
WNT6	ENSBTAG00000013990	ENSBTAT00000018592	9.19	39.69	365	Wnt family member 6
WNT7A	ENSBTAG00000001668	ENSBTAT00000002188	9.05	38.90	349	Wnt family member 7A
WNT7B	ENSBTAG00000048365	ENSBTAT00000083229	9.87	52.40	472	Wnt family member 7B
WNT8A	ENSBTAG00000005570	ENSBTAT00000035270	8.25	39.06	351	Wnt family member 8A
WNT8B	ENSBTAG00000012914	ENSBTAT00000017163	8.88	36.61	333	Wnt family member 8B
WNT9A	ENSBTAG00000020267	ENSBTAT00000081055	8.88	39.85	362	Wnt family member 9A
WNT9B	ENSBTAG00000002664	ENSBTAT00000086580	9.19	38.74	353	Wnt family member 9B
WNT10A	ENSBTAG00000009217	ENSBTAT00000012148	9.36	46.34	417	Wnt family member 10A
WNT10B	ENSBTAG00000015347	ENSBTAT00000020402	9.22	42.96	391	Wnt family member 10B
WNT11	ENSBTAG00000010820	ENSBTAT00000014355	9.14	39.24	354	Wnt family member 11
WNT16	ENSBTAG00000002940	ENSBTAT00000003825	9.04	40.42	362	Wnt family member 16

Mw*:* molecular weight, pI: isoelectric point.

Two unannotated genes from the Wnt family, *ENSBMUG00000022627* and *ENSBMUG00000022624*, were also identified in *Bos mutus*. Further analysis revealed that they both possessed the WNT conserved domain; however, *ENSBMUG00000022627* had an incomplete N-terminus and *ENSBMUG00000022624* had an incomplete C-terminus. These two genes showed the highest sequence similarity and a collinear relationship with Wnt7B, and were temporarily named Wnt7B1 and Wnt7B2 (Supplementary Info File 3).

The amino acid sequences of the 19 bovine Wnt proteins ranged from 333 residues (Wnt8B) to 585 residues (Wnt4); their molecular weight (Mw) ranged from 36.61 to 63.17 kDa. With the exception of Wnt3, which had an isoelectric points (pI) of 7.73, the rest Wnt proteins had a pI higher than 8.0, consistent with their high content in basic amino acids. All 19 Wnt proteins possessed the WNT conserved domain (Supplementary Info File 4).

### Structural features of bovine Wnt family members

**Figure 1:**
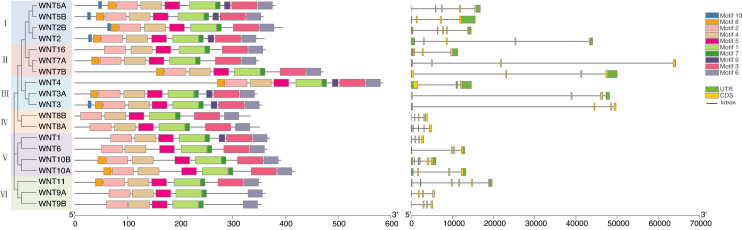
Characterization of the Wnt proteins and genes identified in *Bos taurus*. Left: phylogenetic tree. Middle: motifs of amino acid sequences (conserved motifs shown as colored rectangles). Right: gene structure map, with CDS (yellow rectangle), intron (black line) and UTR (green rectangle).

To investigate the structural characteristics of bovine Wnt proteins and genes, we predicted their phylogenetic relationships based on the conserved motifs and gene structures (Fig. 1). The 19 bovine Wnt family members clustered into six main subfamilies (I–VI). All the Wnt family proteins shared six conserved domains termed motifs 1, 2, 4, 5, 6, and 7 formed by 50, 49, 41, 29, 28 and 9 amino acids, respectively (Supplementary Info File 5). Wnt2, Wnt5A and Wnt5B of the first subfamily, and Wnt3 of the third subfamily had all ten motifs. Wnt2B, Wnt3A and Wnt4A had nine motifs; Wnt2B lacked motif 9, and Wnt3A and Wnt4A lacked motif 10. Wnt7A, Wnt7B, Wnt10A, Wnt10B and Wnt11 (lacking motifs 9 and 10) and Wnt1 (lacking motifs 8 and 10) had eight motifs. Wnt8A, Wnt8B, Wnt11 and Wnt16 possessed seven motifs (lacking motifs 8, 9 and 10). Wnt9A and Wnt9B possessed six motifs (lacking motifs 3, 8, 9 and 10).

Introns, coding sequences (CDS) and untranslated regions (UTR) were variable among the Wnt gene family. For instance, the length of Wnt genes ranged from 3,084 nt (*Wnt1*) to 64,231 nt (*Wnt7A*), mainly due to intron variation. The number of CDS varied from 3 to 6, and the length and layout of the noncoding regions (3’UTR and 5’UTR) were also variable. Despite this variability, the Wnt members in the same evolutionary subfamily tend to possess similar gene structures patterns and conserved motifs.

### Phylogenetic relationship of Wnt proteins in different organisms

**Figure 2:**
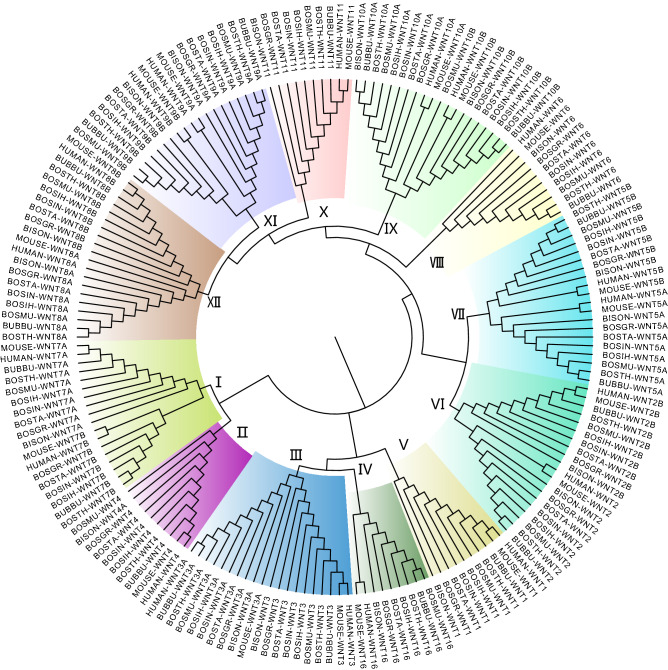
Phylogenetic Neighbor-Joining (NJ) tree of Wnt proteins from ten organisms. Identified Wnt proteins in *Bos taurus* (BOSTA), *Bos grunniens* (BOSGR), *Hybrid-Bos Indicus* (BOSIH), *Hybrid-Bos taurus* (BOSTH), *Bos mutus* (BOSMU), *Bison bison bison* (BISOM), *Bos indicus* (BOSIN) and *Bubalus bubalis* (BUBBU) together with verified Wnts from *Homo sapiens* (HUMAN) and *Mus musculus* (MOUSE). Wnt proteins are grouped into 12 clusters (I–XII) shown as different colors.

Phylogenetic analysis can provide a reference for understanding the functional diversification of the Wnt family in Bovinae. Our phylogenetic analysis included eight species of Bovinae. We also included the Wnt proteins from well-studied model organisms (human and mouse). Of these ten species, 186 amino acid sequences were aligned to generate a non-rooted Neighbor-Joining (NJ) tree (Fig. 2), which showed 12 proposed subfamilies, including Wnt1–11 and Wnt16. There were seven subfamilies containing two Wnt members: I (Wnt7A and Wnt7B), III (Wnt3 and Wnt3A), VI (Wnt2 and Wnt2B), VII (Wnt5A and Wnt5B), IX (Wnt10A and Wnt10B), XI (Wnt9A and Wnt9B), XII (Wnt8A and Wnt8B).

### Chromosomal distribution and collinearity analysis of Wnt genes

**Figure 3:**
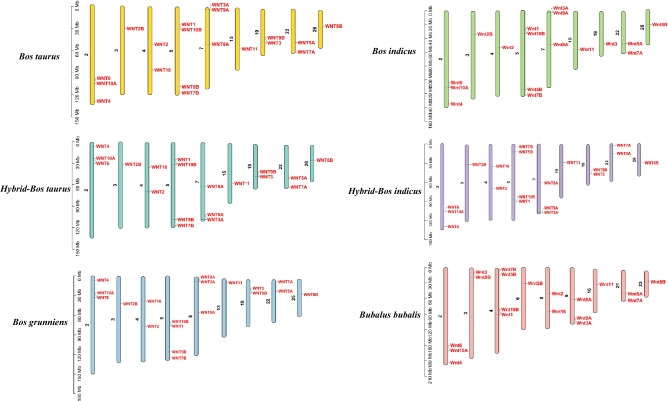
Chromosomal distribution of Wnt genes. The black font on the left represents chromosome numbers and the red font on the right represents Wnt genes.

Wnt genes were mapped on nine chromosomes of cattle (Fig. 3) and the distribution was found to be similar in the other five species. However, the order of *Wnt1* (30,832,513–30,835,596 bp) and *Wnt10B* (30,841,487–30,847,512 bp) in Chr 5, and *Wnt3A* (3,035,810–3,087,361 bp) and *Wnt9A* (3,155,441–3,163,239 Mb) in Chr 7 of *Bos taurus* was reversed from that in *Bos grunniens*. In addition, compared with *Bos taurus*, *Wnt9B* and *Wnt16* were lacking in *Bos Indicus*.

**Figure 4:**
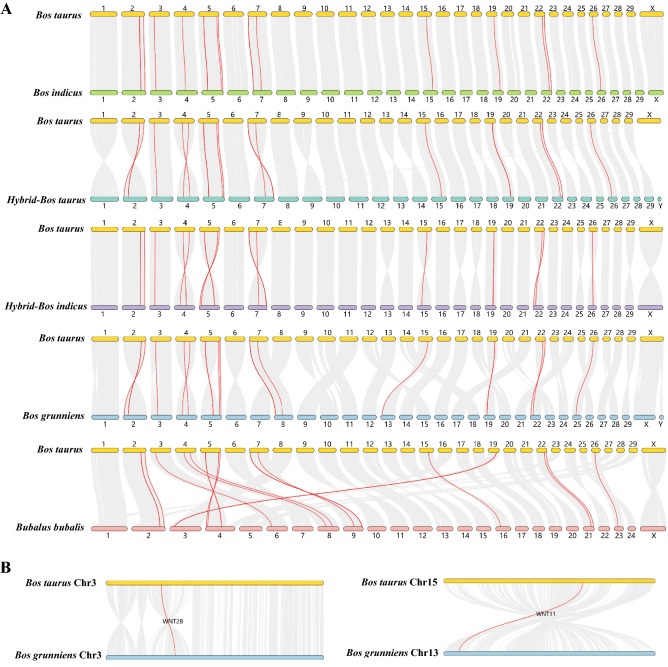
Collinearity analysis of Wnt genes between cattle and other organisms. Syntenic genes pairs are linked by grey lines whereas syntenic Wnt genes are shown as red lines*.*

Genome collinearity analysis revealed a satisfactory corresponding relationship between the chromosomes of *Bos taurus* and *Hybrid-Bos Indicus*, *Hybrid-Bos taurus*, *Bos indicus*, and *Bos grunniens* (Fig. 4A). Although the chromosome number differed between cattle (2 N = 60) and buffalo (2 N = 50), the level of chromosome homology was high between these two species. Also, collinearity modules explained the difference in the position of the Wnt gene family in cattle relative to the other five species in Bovinae. For instance, the position variation of *Wnt2B*, *Wnt11*, *Wnt1* and *Wnt10B* between *Bos taurus* and *Bos grunniens* might have been caused by complex intra-chromosomal translocation events (Fig. 4B). *Wnt3* and *Wnt9B* are distributed on different chromosomes between cattle and buffalo (bovine Chr 19 and buffalo Chr 3). This may be caused by inter-chromosomal rupture or fusion during the evolution (Supplementary Info File 6).

### Expression analysis of Wnt genes in different tissues

**Figure 5:**
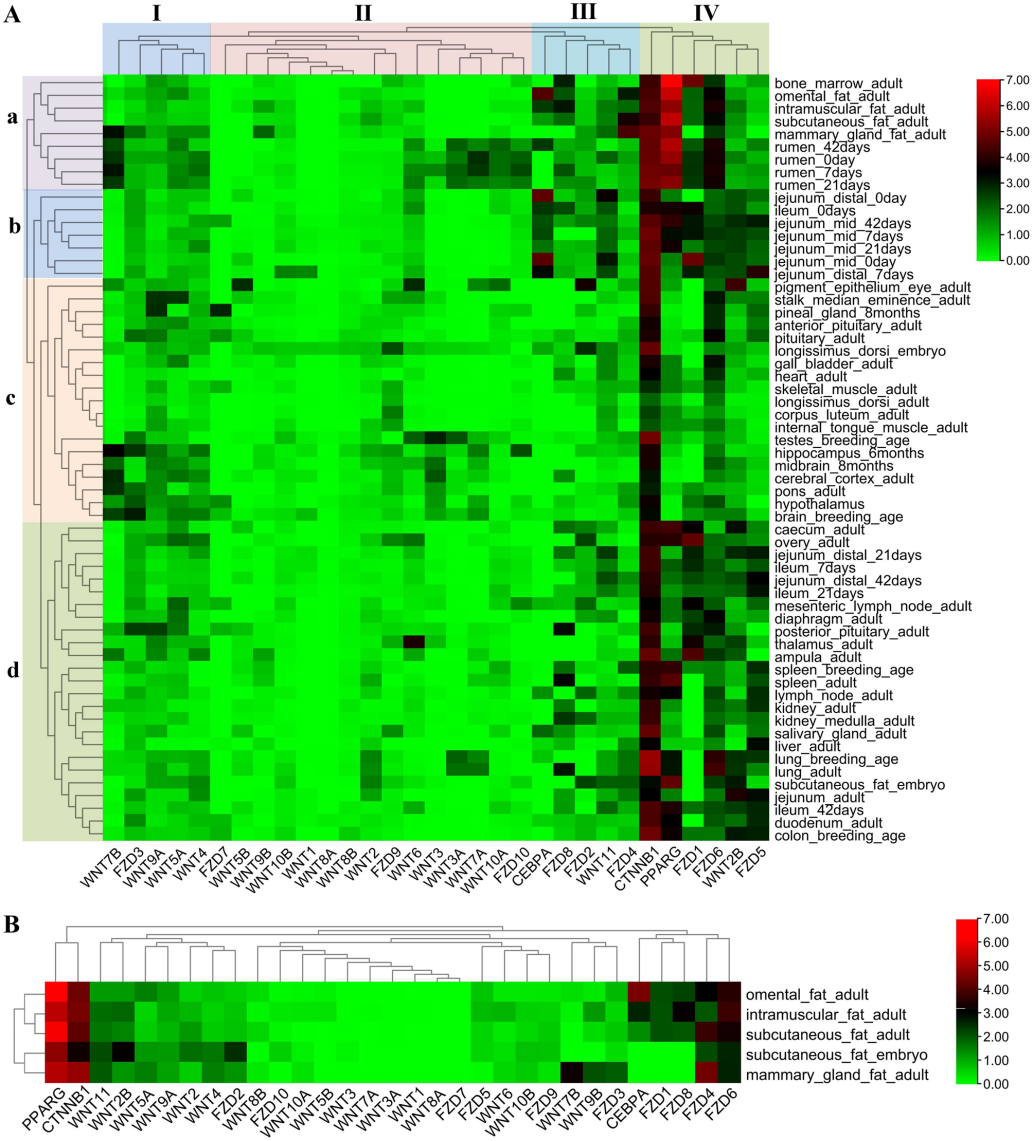
Expression analysis of the Wnt gene family in different bovine tissue types. (**A**) Expression analysis of the Wnt gene family in 60 bovine tissues. The tissues were classified into 4 groups (a to d) and the 31 genes were also classified into 4 groups (I–IV) according to their expression pattern. (**B**) Expression analysis of the Wnt gene family in 5 bovine fat tissues. The horizontal and vertical axis represent genes and bovine tissues, respectively.

Functionally related genes tend to show a co-expression patterns and often regulate biological processes collaboratively. To explore the expression patterns of the Wnt gene family, we investigated their expression levels in 163 samples of 60 tissue types. The Wnt genes along with other 13 closely related genes can be classified into four groups (I to IV) (Fig. 5A) according to their differential expression patterns in tissues. Accordingly, the 60 bovine tissue types also clustered into four main clades (a–d) based on the expression patterns of all the 31 genes including Wnt family. The members of the Wnt family and their receptors, the FZD gene family, displayed overlapping expression patterns, suggesting a coordinated regulatory role. *PPARγ*, a marker gene for adipocyte differentiation, showed high expression in Group a (omental, intramuscular, subcutaneous and mammary gland fats). The cluster formed by *CTNNB1*, *FZD1*, *FZD5*, *FZD6* and *Wnt2B* showed a similar pattern of *PPARγ *in expression.

Further analysis of the five different fat tissues revealed that *CTNNB1*, a core gene of Wnt signaling pathway, showed high expression and similar expression pattern as *PPARγ* (Fig. 5B). High expression was observed for *Wnt7B*, *Wnt9B* and *FZD3* (adult mammary gland fat) and *Wnt2B* and *FZD2* (embryonic subcutaneous fat). Expressions of *C/EBPα*, *FZD1* and *FZD8* were higher in some tissues (omental fat, intramuscular fat (IMF) and subcutaneous fat of adult cattle) than in others (mammary gland fat of adult cattle and subcutaneous fat of embryo). Knowledge of these patterns will provide useful information in bovine fat research. Meanwhile, the clustering analysis of tissue expression pattern revealed that intramuscular fat and subcutaneous fat of adult cattle got together firstly, indicating that they were the most similar among the five types of fat. This also suggests that primary adipocytes isolated from subcutaneous fat can be used for preliminary expression pattern validation of the Wnt gene family.

### Isolation and induced differentiation of bovine primary adipocytes

**Figure 6:**
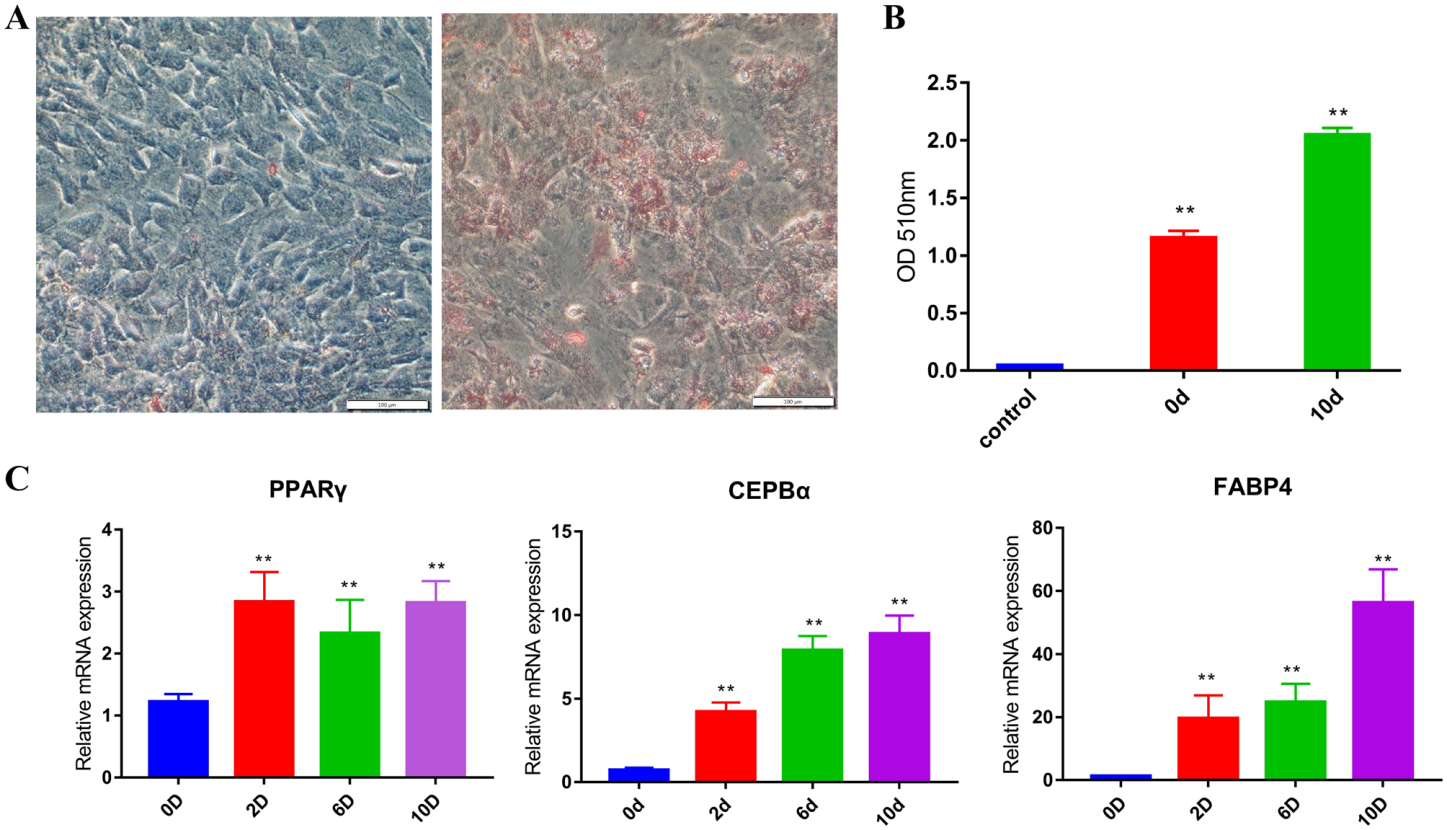
Induced differentiation of primary adipocytes. (**A**) Oil Red O staining of bovine adipocytes induced at day 0 (left) and day 10 (right) of adipogenic differentiation. (**B**) Absorbance at 510 nm. Control: isopropanol, 0d: substance extracted from adipocytes before induction (0 day), 10d: substance extracted from adipocytes 10 days after induction.. (**C**) The expression of adipogenic marker genes during adipogenic differentiation. Symbols * and ** above the bars indicate significant differences at a p value of 0.05 and 0.01 respectively.

Meat tenderness and juiciness are affected by IMF content, whereas it is too limited to be sampled. Subcutaneous fat is significantly associated with IMF^28^, which is consistent with our clustering results (Fig. 5B). To explore the expression patterns of the Wnt gene family during adipocyte differentiation, primary adipocytes collected from subcutaneous adipose tissue of cattle were induced. Ten days after induction, Oil red O staining showed a greater extent of lipid droplet accumulation in adipocytes than in preadipocytes (Fig. 6A). The absorbance at 510 nm was significantly higher in differentiated adipocytes than in preadipocytes (Fig. 6B). Furthermore, the adipogenic marker genes (*PPARγ*, *C/EBPα* and *FABP4*) were all up-regulated (Fig. 6C). These results indicate that the induced differentiation of primary adipocytes was successful and could be used in the subsequent gene expression analysis.

### Expression analysis of Wnt genes during adipocyte differentiation

**Figure 7:**
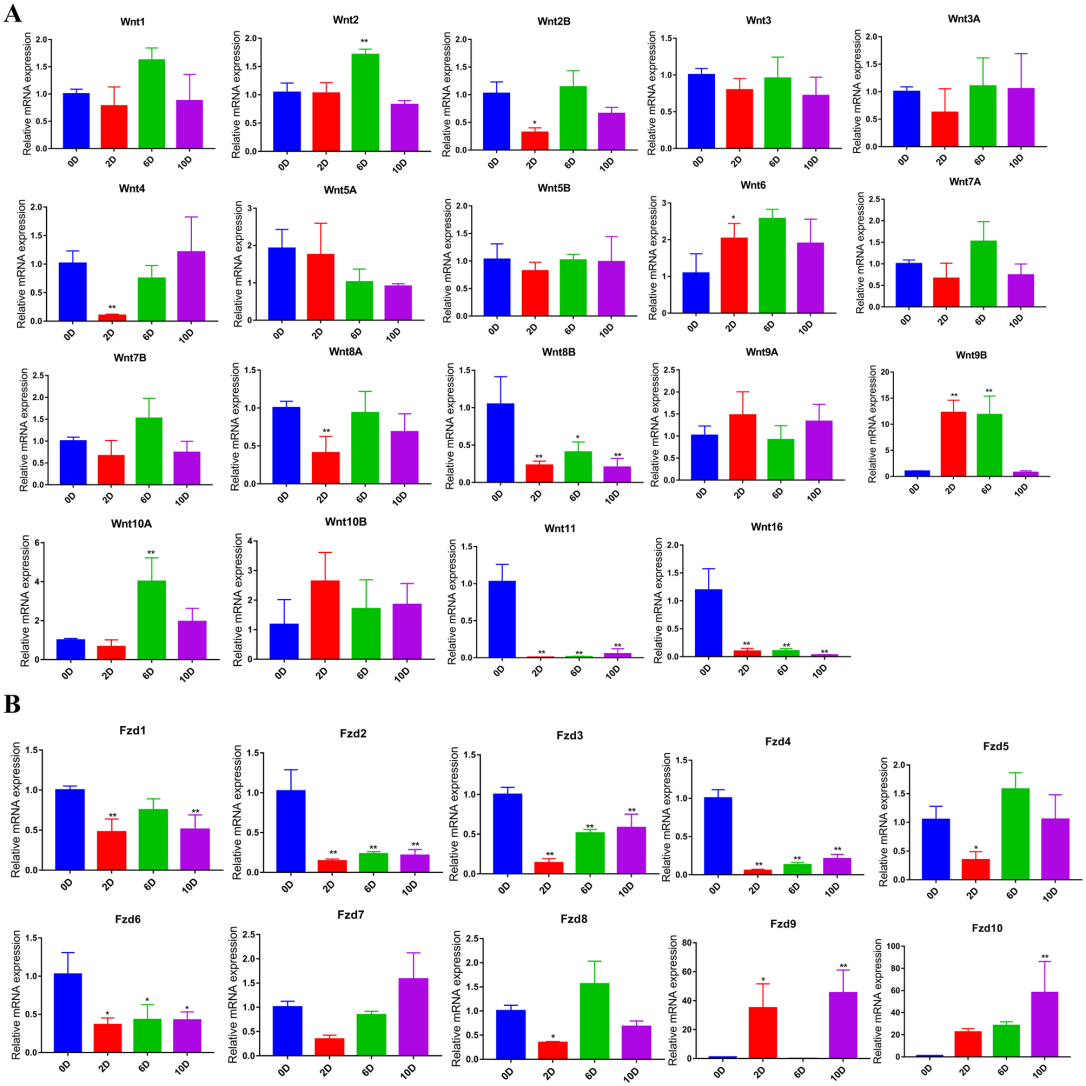
qPCR expression analysis of Wnt and Fzd family genes during adipocyte differentiation. (**A**) Expression of 19 Wnt genes. (**B**) Expression of 10 Fzd genes (n = 3). Symbols * and ** above the bars indicate significant differences at a p value of 0.05 and 0.01 respectively.

qPCR analysis was conducted to detect the expression of Wnt genes and their Fzd receptors at different time points (0, 2, 6, and 10 days) during adipocytes differentiation (Fig. 7). *Wnt8B*, *Wnt11*, *Wnt16* and their receptors (*Fzd1*, *Fzd2*, *Fzd3*, *Fzd4*, *Fzd6*) showed high levels of expression in preadipocytes. These levels were reduced after induction, suggesting a collective involvement in keeping adipocytes undifferentiated. *Wnt2*, *Wnt6*, *Wnt9B*, *Wnt10A* and their receptors (*Fzd9*, *Fzd10*) were significantly up-regulated, indicating a regulatory role during adipocyte differentiation. Furthermore, *Wnt2B*, *Wnt4*, *Wnt8A* and *Fzd5*, *Fzd8* reached the lowest expression at the second day and displayed a similar overall trend of expression*.*

## Discussion

### Structural features of bovine Wnt family proteins and genes

The core motifs and domains of a protein determine its function and activity ^29^. Gene families usually encode proteins that share similar motifs and act synergistically^30^. All the 19 bovine Wnt members have six conserved amino acid sequences (Motifs 1, 2, 4, 5, 6 and 7), pointing to a common functional site. Four members (Wnt2, Wnt3, Wnt5A, and Wnt5A) have ten motifs, whereas the other 15 members lack 1 to 4 of these motifs. Thus, it is likely that these four motifs (Motifs 3, 8, 9 or 10) are not located at the core of the Wnt protein domain.

Since the introns and UTRs vary in length and layout, the distribution of CDSs in the Wnt genes was also variable. Their sequences and conserved motifs were similar, and all possessed the WNT conserved domain. This might play a role in maintaining their three-dimensional structure and binding function.

### The phylogenetic relationships of Wnt family proteins

Phylogenetic analysis provides a credible way to explore the relationship between amino acid sequence similarity and function of proteins in the same family^31^. In multicellular eukaryotes, the Wnt family proteins is divided into 13 subfamilies. For instance, a total of 11 (*Zhikong scallop*), 12 (*Yesso scallop*, *Pacific oyster*) and 13 (*Lingula anatine*, *Plathynereis dumerlii*, *Lottia gigantean*, *Crassostrea gigas*, etc.) subfamilies have been identified previously^20^. In the Bovinae, Wnt proteins were classified into 12 subfamilies but lacked WntA. This was consistent with previous studies reporting that vertebrates all have reserved subfamilies except for WntA^32,33^.

Although the function of the Wnt family is highly conserved, several members have been lost in many species after the complete set of Wnt genes emerged in cnidarians^34,35^. Among the eight species of Bovinae, Wnt7B was missing in *Bison*, while Wnt9B and Wnt16 were missing in *Bos indicus*. The Wnt9 subfamily specific to *Bilateria* was also found to be absent from *Chlamys farreri* ^20^. It remains unclear whether these genes were not identified due to limitations in genome assembly or whether they were lost during evolution.

In the phylogenetic relationship, two genes from distinct species that are located in the same clade are defined as orthologs^36^. The orthologous gene pairs among cattle and the other five bovine species were identified based on homologous relationships. Orthologous Wnt members first clustered in a single clade, indicating that they were conserved among different species.

### Collinearity analysis of Wnts in Bovidae

Genome-wide collinearity analysis of Wnt genes provided key information on the function and evolution in the Bovidae. Gene duplication events can cause gene family expansion during genome evolution^37^. Indeed, both tandem and segmental duplications are responsible for the expansion of the Wnt family in Bovinae (Figs. 3, 4). The members of the Wnt gene family were distributed across nine chromosomes in the six selected species. *WNT9B* and *WNT3* were tandem repeats on chromosome 19 of *Bos Taurus*, while *WNT9B* was missing on chromosome 19 of *Bos Indicus* (Fig. 3). This deletion may be due to the absence of a tandem duplication event or the loss of *WNT9B* after the tandem repeats occurred in *Bos indicus* during evolution. However, the causes, processes, and outcomes of this evolutionary event are still unclear and need more research; such work may further help to clarify the function of *WNT9B*.

Due to the different starting points of chromosome annotation among species, the arrangement of genes might be totally reversed. For instance, in *Bos taurus* and *Hybrid-Bos taurus*, the order of *Wnt6*, *Wnt10A* and *Wnt4* in Chr2, *Wnt2* and *Wnt16* in Chr4, and *Wnt3A*, *Wnt9A* and *Wnt8A* in Chr 7 was opposite.

Intra-chromosomal translocation and rearrangement during species evolution also lead to the changes in gene arrangement^38,39^. For example, the position of *Wnt2B* was altered in *Bos taurus* and *Bos grunniens* due to the inversion of large segments within the chromosome (Fig. 4B). Furthermore, the locations of Wnt genes change from collinear (conserved in the same order) to syntenic (not necessarily in the same order) ^40^. In addition, buffalo Chr 1–5 are collinear with bovine Chr 27 and Chr 1, Chr 23 and Chr 2, Chr 19 and Chr 8, Chr 5 and Chr 28, and Chr 16 and Chr 29, respectively (Supplementary Info File 6). This may be caused by inter-chromosomal rupture or fusion during evolution.

### Wnt genes affecting adipocyte differentiation

The Wnt proteins can activate their Fzd receptors and regulate adipocyte differentiation through the canonical and/or non-canonical Wnt signaling pathway. It is commonly known that functionally related genes usually exhibit similar expression patterns. Furthermore, gene expression clustering analysis can group genes of similar function^41^. Wnt is selective in recognizing its Fzd receptors^42^. For instance, Wnt3 formed a chimera with FZD1 to regulate the canonical Wnt signaling pathway^16^.

The observed overlaps in the expression of 19 Wnt and 10 Fzd members in 60 tissue types suggest a coordinated and selective regulatory role (Fig. 5). Group IV (*PPARG*, *CTNNB1*, *FZD1*, *FZD5*, *FZD6* and *WNT2B*) was highly expressed in four fat tissues (omental, intramuscular, subcutaneous and mammary gland). Meanwhile, previous studies, carried out in humans, showed that Wnt2B and FZD5 exhibited physical interactions and co-expression relationships (Table 2) and displayed similar expression patterns during the differentiation of bovine adipocytes (Fig. 7). Since *PPARG* is a marker gene for adipocyte differentiation and *CTNNB1* is a core gene in the canonical Wnt signaling pathway, WNT2B might bind to its receptor FZD5 to regulate adipogenic differentiation through the canonical Wnt signaling pathway.

**Table 2:** Interaction relationships between *Wnt* and *Fzd* family genes in Human.

Gene symbol	Interaction types with Fzd family genes				
	Physical interation	Co-expression	Predicted	Co-localization	Pathway
WNT1	Fzd8	–	Fzd1, Fzd3, Fzd5, Fzd8	–	Fzd1, Fzd3, Fzd5, Fzd8
WNT2	Fzd1, Fzd9	Fzd1, Fzd2	–	–	Fzd1, Fzd2, Fzd9
WNT2B	Fzd5	Fzd5	–	–	Fzd2, Fzd5
WNT3	Fzd1, Fzd7	–	–	–	Fzd1, Fzd2, Fzd7, Fzd8
WNT3A	Fzd1, Fzd2, Fzd8	–	–	–	Fzd1, Fzd2, Fzd5, Fzd8
WNT4	–	–	Fzd1, Fzd5, Fzd8	–	Fzd1, Fzd2, Fzd5, Fzd8
WNT5A	Fzd1, Fzd2, Fzd5	Fzd1, Fzd2, Fzd10	–	Fzd5	Fzd1, Fzd2, Fzd4, Fzd5, Fzd7, Fzd10
WNT5B	–	Fzd2	–	–	Fzd2
WNT6	–	Fzd2	–	–	Fzd1, Fzd2
WNT7A	Fzd5, Fzd9	Fzd9	–	–	Fzd2, Fzd5, Fzd9
WNT7B	–	Fzd3	Fzd1, Fzd5, Fzd8	–	Fzd1, Fzd2, Fzd3, Fzd5, Fzd8, Fzd10
WNT8A	–	–	–	–	Fzd2
WNT8B	–	Fzd3	–	–	Fzd2, Fzd3, Fzd8
WNT9A	–	–	–	–	Fzd2, Fzd8
WNT9B	–	–	–	–	Fzd2, Fzd8
WNT10A	–	Fzd9	–	–	Fzd2, Fzd8, Fzd9
WNT10B	–	Fzd10	–	–	Fzd2, Fzd8, Fzd10
WNT11	–	Fzd4	–	–	Fzd2, Fzd4
WNT16	–	–	–	–	Fzd2
WNT11	–	Fzd4	–	–	Fzd2, Fzd4
WNT16	–	–	–	–	Fzd2

‘–’ represents no interacting *Fzd.*

Adipogenic differentiation is a well-organized and complicated process regulated by various genes. Analysis of the interactions between the Wnt and the Fzd family is essential to explore their roles. An integrated network for the Wnt and Fzd gene family and their interacting genes were constructed by STRING (https://string-db.org/)^46^ and visualized by Cytoscape (Supplementary Info File 7)^44^. To ensure the accuracy of this interaction network, only sources from literature mining and experimental verification were selected. Analysis showed there were extensive and complex direct or indirect relationships between the Wnt and Fzd gene family. Since such studies have not been carried out in cattle, we used GeneMANIA (https://genemania.org/) to mine their relationship in human. We observed clear bias in the Wnt family members in terms of their ability to recognize their Fzd receptors (Table 2) ^16,42^. Collectively, these results revealed that the Wnt and Fzd genes interact and activate the canonical and/or non-canonical Wnt signaling pathway, thus regulating adipocyte differentiation. The results provide a foundation for further study Wnt genes and the regulation of adipocyte differentiation in cattle.

## Methods

### Ethics statement

Animal experiments were conducted in accordance with the Regulations for the Administration of Affairs Concerning Experimental Animals (Ministry of Science and Technology, China, 2004). It is authorized by the Animal Ethics Committee of Ningxia University (permit number NXUC20200521). The cattle used in the experiments was electric shocked before being released. Primary adipocytes were isolated immediately, making all efforts to minimize its suffering. This work also conformed to the requirements of American Veterinary Medical Association (AVMA) Guidelines. This study is reported in accordance with the recommendations put forward by the ARRIVE guidelines^45^.

### Genome-wide identification of Wnt genes

The genome and annotation of *Bos taurus* (ARS-UCD1.2.101 assembly), *Hybrid-Bos taurus* (*Bos indicus* × *Bos taurus*, UOA_Angus_1.101 assembly), *Hybrid-Bos Indicus* (*Bos indicus* × *Bos taurus*, UOA_Brahman_1.101 assembly), *Bos grunniens* (LU_Bosgru_v3.0.101 assembly), *Bos mutus* (BosGru_v2.0.101 assembly), *Bison bison bison* (Bison_UMD1.0.101 assembly), *Homo sapiens* (GRCh38.101 assembly) and *Mus musculus* (GRCm38.101 assembly) were downloaded from Ensembl database (http://asia.ensembl.org/index.html). The genome and annotation of *Bos indicus* (GCF_000247795.1 assembly) and *Bubalus bubalis* (ASM312139v1 assembly) were downloaded from NCBI database (https://www.ncbi.nlm.nih.gov/). To identify all the possible Wnt genes in the Bovinae, we used the Basic Local Alignment Search Tool (BLAST) and Hidden Markov Model (HMM) searches^46^. A total of 45 reviewed Wnt sequences of bovine (7)*,* human (19) and mouse (19) were obtained from the UniProt database (https://www.uniprot.org/) and used to query potential Wnt genes via BLASTP with a threshold e-value of 10^**–**5^. The HMM of Wnt (PF00110) was downloaded from Pfam (https://pfam.xfam.org/)^50^ and HMMER 3.3.1 (http://hmmer.org/)^51^ was used to construct HMM profiles for the Bovidae to detect Wnt genes with the default settings. Candidate sequences were manually checked to confirm Wnt homology.

### Analysis of Wnt protein characteristics

The properties of the proteins encoded by the identified Wnt genes were obtained from ExPASy (https://web.expasy.org/protparam/)^49^. Conserved motifs were detected in MEME 5.0^50^ with minimum and maximum lengths of 6 and 50 amino acids, respectively. Exon–intron structures and motif patterns of the Wnt family were visualized using TBtools (v1.0971) software^51^which can be downloaded from the GitHub website (https://github.com/CJ-Chen/TBtools/releases). NCBI-CDD^52^ was used to identify the conservative domains in the 19 Wnt proteins, which were visualized by the TBtools software^51^. Multiple sequence alignments for Wnt proteins and a phylogenetic Neighbor-Joining tree were constructed using the MEGA 7.0 software^53^, which can be downloaded from the website at https://mega.software.informer.com/7.0/.

### Phylogenetic tree construction, chromosomal distribution, and collinearity analysis

Protein sequences were aligned using ClustalW to investigate the phylogenetic relationship of Wnt genes. A Neighbor-Joining tree was constructed in MEGA 7.0^53^ using the bootstrap method (1000 replicates), the Poisson model, and complete deletion. FigTree software (version 1.4.3) was used to adjust and enhance the evolutionary tree and it can be downlord from the GitHub website (https://github.com/rambaut/figtree/releases). The chromosomal locations of Wnt genes in cattle and the five other species of Bovinae were obtained from general feature format (GFF3) files. Gene Location Visualize from GFF^51^ was used to map the distribution of Wnt genes. Collinearity analysis for orthologous genes between *Bos taurus* and the five other bovine species was performed using the MCScanX toolkit^54^. Subsequently, genome collinearity results and orthologous Wnt genes were visualized by Dual Systeny Plot for MCscanX^51^.

### The tissue expression profiles analysis of Wnt genes

RNA-Seq data of 163 tissue samples were collected from the Ruminant Genome Database (http://animal.nwsuaf.edu.cn/code/index.php/Ruminantia)^55^ and all the raw data was deposited in the National Center for Biotechnology Information (NCBI) Sequence Read Archive (Supplementary Info File 8). The sequencing quality was checked using FastQC^56^. Quality control of the raw sequence data was performed using the trimmomatic-0.36 ^57^. Clean reads were then mapped to the *Bos taurus* genome reference using STAR^58^ and Hisat2^59^. FPKMs (Fragments Per Kilobase per Million mapped reads) of the genes in each sample were computed by Ballgown (Version 2.2.0)^60,61^. The heatmap was constructed using the TBtools software^51^.

### Isolation, culture, and induction differentiation of bovine primary adipocytes

Primary adipocytes were isolated and cultured from subcutaneous adipose tissue of the cattle in the Zerui Ecological Breeding Farm using the Type I collagenase digestion method. Induction of primary adipocytes differentiation^62^ and Oil red O staining^63^ were performed as previous described. The absorbance of the substance extracted from adipocytes at 0 day and 10 day after induction was also measured at 510 nm with isopropanol as a control.

### RNA extraction and quantitative RT-PCR (qPCR)

Primers for the Wnt genes were designed using Primer Premier 5.0 software (Supplementary Info File 9). Total RNA was extracted by the phenol–chloroform method using TRIzol (9109, Takara) and samples with an OD260/OD280 absorbance ratio between 1.8 and 2.0 were used in the subsequent experiments. Then, a total of 1000 ng RNA was reverse transcribed using random primers with Moloney murine leukemia virus reverse transcriptase (Takara Bio, Kyoto, Japan). Realtime PCR was carried out in a CFX96 Touch Real-Time PCR Detection System (Bio-Rad, Hercules, CA, USA) with SYBR Green Master Mix (Takara Bio, Kyoto, Japan). The qPCR reaction procedure involved 40 cycles of pre-denaturation for 3 min (95 °C), denaturation for 10 s (95 °C), annealing for 20 s (60 °C), and extension for 30 s (72 °C). Relative expression was calculated using the 2^−∆∆Ct^ method^64,65^ with β-actin as the reference. Three replicates were performed for each test. One-way analysis of variance (ANOVA) was applied to test the statistical significance among groups at 0.05 and 0.01 significance levels using GraphPad Prism 7.0 software.

### Ethics approval and consent to participate

The Animal Ethics Committees of Ningxia University approved the experimental design and animal sample collection (permit number NXUC20200521). We also obtained informed consent from the owners and the Ethics Committees of Zerui Ecological Breeding Farm. All the animal experiments were conducted according to the guidelines of the Regulations for the Administration of Affairs Concerning Experimental Animals (Ministry of Science and Technology, China, 2004). This study is reported according to the recommendations put forward in the ARRIVE guidelines.

## Supplementary Information

Supplementary Information 1.

Supplementary Information 2.

Supplementary Information 3.

Supplementary Information 4.

Supplementary Information 5.

Supplementary Information 6.

Supplementary Information 7.

Supplementary Information 8.

Supplementary Information 9.

## Data Availability

All of the data generated or analyzed during this study are included in this published article and its supplementary information files.
