# Supplementary material for: Genome-wide identification and expression profiling analysis of Wnt family genes affecting adipocyte differentiation in cattle
Source: Sci Rep. 2022 Jan 11;12:489. doi: 10.1038/s41598-021-04468-1 (PMC8752766; doi:10.1038/s41598-021-04468-1)
Supplement: Supplementary file 2 — Supplementary Information 2. [file 41598_2021_4468_MOESM2_ESM.doc]

**Supplementary Info File 2. Protein sequences of Wnt family members in ten species**

>BOSTA-WNT5A

MKKSIGILSPGVAWGTAGRAMSSKFFLMALAIFLSFAQVVIEANSWWSLGMNNPVQMSEV

YIIGAQPLCSQLAGLSQGQKKLCHLYQDHMQYIGEGAKTGIKECQYQFRHRRWNCSTVDN

TSVFGRVMQIGSRETAFTYAVSAAGVVNAMSRACREGELSTCGCSRAARPKDLPRDWLWG

GCGDNIDYGYRFAKEFVDARERERIHAKGSYESARILMNLHNNEAGRRTVYSLADVACKC

HGVSGSCSLKTCWLQLADFRKVGDALKEKYDSAAAMRLNSRGKLVQVNSRFNSPTTQDLV

YIDPSPDYCVRNESTGSLGTQGRLCNKTSEGMDGCELMCCGRGYDQFKTVQTERCHCKFH

WCCYVKCKKCTEIVDQFVCK

>BOSTA-WNT11

MRARPQVCQALLFALALQTGVCYGIKWLALSKTPAALALNQTQHCKQLEGLVSAQVQLCR

SNLELMHTIVHAAREVMKACRRAFADMRWNCSSIELAPNYLLDLERGTRESAFVYALSAA

AISHAIARACTSGDLPGCSCGPVPGEPPGPGNRWGGCADNLSYGLLMGAKFSDAPMKVKK

TGSQANKLMRLHNSEVGRQALRASLEMKCKCHGVSGSCSIRTCWKGLQELRDVAADLKTR

YLSATKVVHRPMGTRKHLVPKDLDIRPVKDSELIYLQSSPDFCMKNEKVGSHGTQDRQCN

KTSHGSDSCDLMCCGRGYNPYTDRVVERCHCKYHWCCYVTCRRCERTVERYVCK

>BOSTA-WNT1

MGHWALLPCWVSAALLLALAALPAALAANSSGRWWGIVNVASSTNLLTDSKSLQLVLEPS

LQLLSRKQRRLIRQNPGILHSVSGGLQSAVRECKWQFRNRRWNCPTASGPHLFGKIVNRG

CRETAFIFAITSAGVTHSVARSCSEGSIESCTCDYRRRGPGGPDWHWGGCSDNIDFGRLF

GREFVDSGEKGRDLRFLMNLHNNEAGRTTVFSEMRQECKCHGMSGSCTVRTCWMRLPTLR

AVGDVLRDRFDGASRVLYGNRGNNRASRAELLRLEPEDPAHKPPSPHDLVYFEKSPNFCT

YSGRLGTAGTAGRACNSSSPALDGCELLCCGRGHRTRTQRVTERCNCTFHWCCHVSCRNC

THTRVLHECL

>BOSTA-WNT10B

MREEPRPRPPPSGLAGLLFLALCSRALGNEIQGLKLPGGGEPPLTANTVCLTLSGLSKQQ

LGLCLRSPDVTASALQGLHIAVHECQHQLRDQRWNCSALEGGGRLPHHSAILKRGFRESA

FSFSMLAAGVMHAVATACSLGKLVSCGCGWKGSGEQDRLRAKLLQLQALSRGKSFSHSLP

SSGPGSGPSPGPQDTWEWGGCNHDMDFGEKFSRDFLDSREAPRDIQARMRIHNNRVGRQV

VTENLKRKCKCHGTSGSCQLKTCWRAPPEFRAVGAALRERLDRAIFIDTHNRNSGAFQPR

LRPRRLSGELVYFENSPDFCERDPTVGSPGTQGRACNKTSHQLGSCGSLCCGRGHNVLRQ

TRVERCNCRFHWCCYVLCDECKVTEWVNVCK

>BOSTA-WNT4

MGAMKPPLTCTWSQSALTESCVIWPGIPFQPCEVGQVLMPHPRAVKNPEAEQGAWHTLFS

KLTRPHCRPQPPVLTAHIVLSATAVILVSAWGPGKWLAMRIAAFVSQKVGLGHGGPCLGM

REGDTADQTAAPRLPDRGHLDMQGLEWTRSGVTSGLRCRWPRGPPGGEGSHGGSVSGSPR

ASFNSSRDRGLVQDSPSPLLPSTAQAGGLSLDPERSQGLIAGTPGSQSPGHGGGSPPGRG

RRRGGALPGIPPPPNPALPRYLAKLSSVGSISEEETCEKLKGLIQRQVQMCKRNLEVMDS

VRRGAQLAIEECQYQFRNRRWNCSTLDSLPVFGKVVTQGTREAAFVYAISSAGVAFAVTR

ACSSGELEKCGCDRTVHGVSPQGFQWSGCSDNIAYGVAFSQSFVDVRERSKGASSSRALM

NLHNNEAGRKAILTHMRVECKCHGVSGSCEVKTCWRAVPPFRQVGHALKEKFDGATEVEP

RRVGSSRALVPRNAQFKPHTDEDLVYLEPSPDFCEQDVRSGVLGTRGRTCNKTSKAIDGC

ELLCCGRGFHTAQVELAERCSCKFHWCCFVKCRQCQRLVELHTCR

>BOSTA-WNT8B

FLSSRSVNNFLMTGPKAYLIYSSSVAAGAQSGIEECKYQFAWDRWNCPERALQLSSHGGL

RSANRETAFVHAISSAGVMYTLTRNCSLGDFDNCGCDDSRNGQLGGQGWLWGGCSDNVGF

GEAISKQFVDALETGQDARAAMNLHNNEAGRKAVKGTMKRTCKCHGVSGSCTTQTCWLQL

PEFREVGAHLKEKYHAALKVDLLQGAGNSAAGRGAIADTFRSISTRELVHLEDSPDYCLE

NKTLGLLGTEGRECLRRGRALGRWERRSCRRLCGDCGLAVEERRAETVSSCNCKFHWCCA

VRCEQCRRRVTKYFCSRADRPRGGAAHEPGRKP

>BOSTA-WNT5B

MPSLPALLALLFACWAPLRATASSWWSLAMSPVQRPEMFIIGAQPVCSQLPGLSAGQRKL

CQLYQEHMAYIGEGARTGIRECQHQFRQRRWNCSTVDDASVFGRVLQIGSRETAFTYAVS

AAGVVNAISRACREGELSTCGCSRAARPKDLPRDWLWGGCGDNVDYGYRFAKEFVDARER

EKNFAKGSEEQGRVLMNLQNNEAGRRAVYKTADVACKCHGVSGSCSLKTCWLQLAEFRKV

GDQLKEKYDSAAAMRITRRGKLELVNSRFKPPTPEDLVYVDPSPDYCLRDESTGSLGTRG

RLCNKTSEGLDGCALMCCGRGYNQFKSVRTERCHCKFHWCCFVRCKKCTQVVDQFVCK

>BOSTA-WNT8A

MGDLLILRVAVGICYVTFSASAWSVNNFLITGPKAYLTYTTSVALGAQSGIEECKFQFAW

ERWNCPENALQLSTHNRLRSATRETSFIHAISSAGVMYTITKNCSMGDFENCGCDESKNG

KTGGHGWIWGGCSDNVEFGERISKLFVDSLEKGKDARALMNLHNNRAGRLAVRATMKRTC

KCHGISGSCSIQTCWLQLANFRELGNYLKAKYERALKIEMDKQQLRAGNSAEGHWIPTEA

FLPSAEAELIFLEESPDYCTRNSSLGIYGTEGRECLQNSRNTSRWEQCSCGRLCTECGLQ

VEERRTEAISSCNCKFQWCCTVKCEQCRHVVNKYYCTSSPGSAQSWGKGSA

>BOSTA-WNT3A

MGHKGTSPTNQASLSSPRSLAVGPQYSSLGTQPILCASIPGLVPKQLRFCRNYVEIMPSV

AEGIKISIQECQHQFRGRRWNCTTINNSLAIFGPVLDKATRESAFVHAIASAGVAFAVTR

SCAEGSAAICGCSSRHQGSPGEGWKWGGCSEDIEFGGMVSREFADARENRPDARSAMNRH

NNEAGRQAIASHMHLKCKCHGLSGSCEVKTCWWSQPDFRAIGDFLKDKYDSASEMVVEKH

RESRGWVETLRPRYTYFKVPTERDLVYYEASPNFCEPNPETGSFGTRDRTCNVSSHGIDG

CDLLCCGRGHNARTEQRREKCHCVFHWCCYVSCQECARVYDVHTCK

>BOSTA-WNT9A

VCSGDLVASLPSCCLGLSTLLSCSQGADQLTGSEPLTILPLTLEPEAVAQAHYKACDRLK

LERKQRRMCRRDPGVAETLVEAVSMSALECQYQFRFERWNCTLEGRYRASLLKRGFKETA

FLYAISSAGLTHALAKACSAGRMERCTCDEAPDLENREAWQWGGCGDNLKYSSKFVKEFL

GRRSSKDLRARVDFHNNLVGVKVIKAGVETTCKCHGVSGSCTVRTCWRQLAPFHEVGKRL

KHKYETALKVGSTTNEATGEAGAISPPRGRAAGAGGGDPLPRTPELVHLDDSPSFCVAGR

FSPGTAGRRCHREKNCESICCGRGHNTQSRVVTRPCQCQVRWCCYVECRQCTQREEVYTC

KG

>BOSTA-WNT2B

MLRPGGAEEAAQLPPRRVSAPVPESAPRSTAPDGSRASARLSLACLLLLLLLLTLPARVD

TSWWYIGALGARVICDNIPGLVSRQRQLCQRYPDIMRSVGEGAREWIRECQHQFRHHRWN

CTTLDRDHTVFGRVMLRSSREAAFVYAISSAGVVHAITRACSQGELSVCSCDPYTRGRHH

DQRGDFDWGGCSDNIHYGVRFAKAFVDAKEKRLKDARALMNLHNNRCGRTAVRRFLKLEC

KCHGVSGSCTLRTCWRALSDFRRTGDYLRRRYDGAVQVTATQDGANFTAARQGYRRATRT

DLVYFDNSPDYCVLDKAAGSLGTAGRVCSKTSKGTDGCEIMCCGRGYDTTRVTRVTQCEC

KFHWCCAVRCKECRNTVDVHTCKAPKKAEWLDQT

>BOSTA-WNT9B

FSFPFLLLLLSLVTGLPSLAPFSLTGREVLTPFPGLGTAAPAQGGAHLKQCDLLKLSRRQ

KQLCRREPGLAETLQDAAHLSLLECQFQFRHERWNCSLEGRTGLLKRGFKETAFLYAVSA

AALTHTLARACSAGRMERCTCDDSPGLESRQAWQWGVCGDNLKYSTKFLNNFLGPKRGSK

DLRARADAHNTHVGIKAVKSGLRTTCKCHGVSGSCAVRTCWKQLSPFRDTGQVLKLRYDS

AVKVSSASNEALGRLELWAPARPGSPSKGPAPRPGDLVYMEDSPSFCRPSKYSPGTGGRV

CSREASCSSLCCGRGYDTQSRLTAFSCHCQVQWCCYVECQQCVREELVYTCKH

>BOSTA-WNT3

MEPHLLRLLLGLLLCGTRVLAGYPIWWSLALGQQYTSLGSQPLLCGSIPGLVPKQLRFCR

NYIEIMPSVAEGVKLGIQECQHQFRGRRWNCTTIDDSLAIFGPVLDKATRESAFVHAIAS

AGVAFAVTRSCAEGTSTICGCDSHHKGPPGEGWKWGGCSEDADFGVLVSREFADARENRP

DARSAMNKHNNEAGRTTILDHMHLKCKCHGLSGSCEVKTCWWAQPDFRAIGDFLKDKYDS

ASEMVVEKHRESRGWVETLRAKYALFKPPTERDLVYYENSPNFCEPNPETGSFGTRDRTC

NVTSHGIDGCDLLCCGRGHNTRTEKRKEKCHCIFHWCCYVSCQECIRIYDVHTCK

>BOSTA-WNT7A

MNRKARRCLGHLFLSLGMVYLRIGGFSSVVALGASIICNKIPGLAPRQRAICQSRPDAII

VIGEGSQMGLDECQFQFRNGRWNCSALGERTVFGKELKVGSREAAFTYAIIAAGVAHAIT

AACTQGNLSDCGCDKEKQGQYHRDEGWKWGGCSADIRYGIGFAKVFVDAREIKQNARTLM

NLHNNEAGRKILEENMKLECKCHGVSGSCTTKTCWTTLPQFRELGYVLKDKYNEAVHVEP

VRASRNKRPAFLKIKKPLSYRKPMDTELVYIEKSPSYCEEDPATGSVGTQGRACNKTAPQ

ASGCDLMCCGRGYNTHQYARVWQCNCKFHWCCYVKCNTCSERTEVYTCK

>BOSTA-WNT7B

MAAGRLRRLEDVRLSERQSWFRPIDGRRAGRRARRTQRPRPAPPPGPPASARAAAPAPAR

CEALEAGHGRPLRRGPRARIPSAPFRRPALGRVAETPAPAGPRALPAGPARPPRERRPTM

LLLSPRSALLSVYCPQLFLILSSGSYLALSSVVALGANIICNKIPGLAPRQRAICQSRPD

AIIVIGEGAQMGINECQYQFRFGRWNCSALGEKTVFGQELRVGSREAAFTYAITAAGVAH

AVTAACSQGNLSNCGCDREKQGYYNQAEGWKWGGCSADVRYGIDFSRRFVDAREIKKNAR

RLMNLHNNEAGRKVLEERMKLECKCHGVSGSCTTRTCWTTLPKFREVGHLLKEKYNVAVQ

VEVVRASRLRQPTFLRIKQLRSYQKPMETDLVYIEKSPNYCEEDAATGSVGTQGRLCNRT

SPGADGCDTMCCGRGYNTHQYTKVWQCNCKFHWCCFVKCNTCSERTEVFTCK

>BOSTA-WNT2

MNACLVGIWLWLPLLFTWLSPEVSSSWWYMRATSGSSRVMCDNVPGLVSHQRQLCHRHPD

VMRAIGLGVTEWTMECQHQFRQHRWNCNTLDRDHSLFGRVLLRSSRESAFVYAISSAGVV

FAITRACSQGELKSCSCDPKKKGTAKDNKGTFDWGGCSDNIDYGIKFARAFVDAKERKGK

DARALMNLHNNRAGRKAVKRFLKQECKCHGVSGSCTLRTCWLAMADFRKTGNYLWRKYNG

AIQVVMNQDGTGFTVANKRFKKPTKNDLVYFENSPDYCIRDRDAGSLGTAGRVCNLTSRG

MDSCEVMCCGRGYDTSHITRKTKCECKFHWCCAVRCQDCVEALDVHTCKAPKSPDWAAPT

>BOSTA-WNT6

MQPPAPSRLGLLLLLLLSPAHVGGLWWAVGSPLVMDPTSICRKARRLAGRQAELCQAEPE

VVAELARGARLGVRECQFQFRFRRWNCSSHSKAFGRILQQDIRETAFVFAITAAGASHAV

TQACSMGELLQCGCQAPRGRAPPRPPGLPGTPGPPGPAGSPDGSAAWEWGGCGDDVDFGD

EKSRLFMDAQHKRGRGDIRMLVQLHNNEAGRLAVRSHTRTECKCHGLSGSCALRTCWQKL

PPFREVGARLLERFHGASRVMGTNDGKALLPAVRTLKPPGRADLLYAADSPDFCAPNRRT

GSPGTRGRACNSSAPDLSGCDLLCCGRGHRQESVLLEENCLCRFHWCCVVQCHRCRVRKE

LSLCL

>BOSTA-WNT10A

MGSTHPCPWLRLRPRPQPRPALCALLFFLLLLAASVPRSAPNDILGLRLPQEPVLNANTV

CLTLPGLSKRQMEVCVRHPDVAASAIQGIQIAIHECQHQFRDQRWNCSSLETRNKIPYES

PIFSRGFRESAFAYAISAAGVVHAVSNACALGKLRACGCDASRRGDEEAFRRKLHRLQLE

ALQRGKGLSHGVPEHPALPPASPGLQDSWEWGGCSPDVGFGERFSKDFLDSREPHRDIHA

RMRLHNNRVGRQAVMENMRRKCKCHGTSGSCQLKTCWQVTPEFRAVGALLRSRFHRATLI

RPHNRNSGQLEPGPAGAPSPAPGLPGPRRRASPADLVYFEKSPDFCEREPRLDSAGTVGR

LCNKSSAGPDGCGSMCCGRGHNILRQTRSERCHCRFHWCCFVVCEECRITEWVSVCK

>BOSTA-WNT16

MDRAALLGLSRLCALWAAVLALFPCGAQGNWMWLGIASFGVPEKLGCANLPLNSRQKELC

KRKPYLLPSIREGARLGIQECRSQFRHERWNCLVAAASAPGTSPLFGYELSSGTKETAFI

YAVMAAGLVHSVTRSCSAGNMTECSCDTTLQNGGSASEGWHWGGCSDDVQYGMWFSRKFL

DFPIKNTTAKESKVLLAMNLHNNEAGRQAVAKLMSLDCRCHGVSGSCAVKTCWKTMSSFE

KIGHLLKDKYENSVQISDKIKRKMHRREKDQRKIPIRKDDLLYVNKSPNYCVEDKKLGIP

GTQGRECNRTSEGADGCNLLCCGRGYNTHVVRHVERCECKFIWCCYVRCRRCESMTDVHT

CK

>BOSIN-WNT2B

MLRPGGAEEAAQLPPRRVSAPVPESAPRSTAPDGSRASARLSLACLLLLLLLLTLPARVDTSWWYIGALGARVICDNIPG

LVSRQRQLCQRYPDIMRSVGEGAREWIRECQHQFRHHRWNCTTLDRDHTVFGRVMLRSSREAAFVYAISSAGVVHAITRA

CSQGELSVCSCDPYTRGRHHDQRGDFDWGGCSDNIHYGVRFAKAFVDAKEKRLKDARALMNLHNNRCGRTAVRRFLKLEC

KCHGVSGSCTLRTCWRALSDFRRTGDYLRRRYDGAVQVTATQDGANFTAARQGYRRATRTDLVYFDNSPDYCVLDKAAGS

LGTAGRVCSKTSKGTDGCEIMCCGRGYDTTRVTRVTQCECKFHWCCAVRCKECRNTVDVHTCKAPKKAEWLDQT

>BOSIN-WNT2

MNACLVGIWLWLPLLFTWLSPEVSSSWWYMRATSGSSRVMCDNVPGLVSHQRQLCHRHPDVMRAIGLGVTEWTMECQHQF

RQHRWNCNTLDRDHSLFGRVLLRSSRESAFVYAISSAGVVFAITRACSQGELKSCSCDPKKKGTAKDNKGTFDWGGCSDN

IDYGIKFARAFVDAKERKGKDARALMNLHNNRAGRKAVKRFLKQECKCHGVSGSCTLRTCWLAMADFRKTGNYLWRKYNG

AIQVVMNQDGTGFTVANKRFKKPTKNDLVYFENSPDYCIRDRDAGSLGTAGRVCNLTSRGMDSCEVMCCGRGYDTSHITR

KTKCECKFHWCCAVRCQDCVEALDVHTCKAPKSPDWAAPT

>BOSIN-WNT7B

MAGEGLGLDRGLSAAALGGGCPSGPGPAPLGGGQLAGQLQSPMFCDSCTPPGSSVIALSSVVALGANIICNKIPGLAPRQ

RAICQSRPDAIIVIGEGAQMGINECQYQFRFGRWNCSALGEKTVFGQELRVGSREAAFTYAITAAGVAHAVTAACSQGNL

SNCGCDREKQGYYNQAEGWKWGGCSADVRYGIDFSRRFVDAREIKKNARRLMNLHNNEAGRKVLEERMKLECKCHGVSGS

CTTRTCWTTLPKFREVGHLLKEKYNVAVQVEVVRASRLRQPTFLRIKQLRSYQKPMETDLVYIEKSPNYCEEDAATGSVG

TQGRLCNRTSPGADGCDTMCCGRGYNTHQYTKVWQCNCKFHWCCFVKCNTCSERTEVFTCK

>BOSIN-WNT1

MGHWALLPCWVSAALLLALAALPAALAANSSGRWWGIVNVASSTNLLTDSKSLQLVLEPSLQLLSRKQRRLIRQNPGILH

SVSGGLQSAVRECKWQFRNRRWNCPTASGPHLFGKIVNRGCRETAFIFAITSAGVTHSVARSCSEGSIESCTCDYRRRGP

GGPDWHWGGCSDNIDFGRLFGREFVDSGEKGRDLRFLMNLHNNEAGRTTVFSEMRQECKCHGMSGSCTVRTCWMRLPTLR

AVGDVLRDRFDGASRVLYGNRGNNRASRAELLRLEPEDPAHKPPSPHDLVYFEKSPNFCTYSGRLGTAGTAGRACNSSSP

ALDGCELLCCGRGHRTRTQRVTERCNCTFHWCCHVSCRNCTHTRVLHECL

>BOSIN-WNT10B

MREEPRXXPPPLGLAGLLFLALCSRALGNEIQGLKLPGGGEPPLTANTVCLTLSGLSKQQLGLCLRSPDVTASALQGLHI

AVHECQHQLRDQRWNCSALEGGGRLPHHSAILKRGFRESAFSFSMLAAGVMHAVATACSLGKLVSCGCGWKGSGEQDRLR

AKLLQLQALSRGKSFSHSLPSSGPGSGPSPGPQDTWEWGGCNHDMDFGEKFSRDFLDSREAPRDIQARMRIHNNRVGRQV

VTENLKRKCKCHGTSGSCQLKTCWRAPPEFRAVGAALRERLDRAIFIDTHNRNSGAFQPRLRPRRLSGELVYFENSPDFC

ERDPTVGSPGTQGRACNKTSHQLGSCGSLCCGRGHNVLRQTRVERCNCRFHWCCYVLCDECKVTEWVNVCK

MPSLPALLALLFACWAPLRATASSWWSLAMSPVQRPEMFIIGAQPVCSQLPGLSAGQRKLCQLYQEHMAYIGEGARTGIR

ECQHQFRQRRWNCSTVDDASVFGRVLQIGSRETAFTYAVSAAGVVNAISRACREGELSTXXXXRAARPKDLPRDWLWGGC

GDNVDYGYRFAKEFVDAREREKNFAKGSEEQGRVLMNLQNNEAGRRAVYKTADVACKCHGVSGSCSLKTCWLQLAEFRKV

GDQLKEKYDSAAAMRITRRGKLELVNSRFKPPTPEDLVYVDPSPDYCLRDESTGSLGTRGRLCNKTSEGLDGCALMCCGR

GYDQFKSVRTERCHCKFHWCCFVRCKKCTQVVDQFVCK

>BOSIN-WNT5B

MPSLPALLALLFACWAPLRATASSWWSLAMSPVQRPEMFIIGAQPVCSQLPGLSAGQRKLCQLYQEHMAYIGEGARTGIR

ECQHQFRQRRWNCSTVDDASVFGRVLQIGSRETAFTYAVSAAGVVNAISRACREGELSTXXXXRAARPKDLPRDWLWGGC

GDNVDYGYRFAKEFVDAREREKNFAKGSEEQGRVLMNLQNNEAGRRAVYKTADVACKCHGVSGSCSLKTCWLQLAEFRKV

GDQLKEKYDSAAAMRITRRGKLELVNSRFKPPTPEDLVYVDPSPDYCLRDESTGSLGTRGRLCNKTSEGLDGCALMCCGR

GYDQFKSVRTERCHCKFHWCCFVRCKKCTQVVDQFVCK

>BOSIN-WNT9A

MQRRCLVGRGPRGSPPALLPSGSAAAPASAAAHPVPASLLWSRAHFGLCVPRLTGSEPLTILPLTLEPEAVAQAHYKACD

RLKLERKQRRMCRRDPGVAETLVEAVSMSALECQYQFRFERWNCTLEGRYRASLLKRGFKETAFLYAISSAGLTHALAKA

CSAGRMERCTCDEAPDLENREAWQWGGCGDNLKYSSKFVKEFLGRRSSKDLRARVDFHNNLVGVKVIKAGVETTCKCHGV

SGSCTVRTCWRQLAPFHEVGKRLKHKYETALKVGSTTNEATGEAGAISPPRGRAAGAGGGDPLPRTPXXVHLDDSPSFCV

AGRFSPGTAGRRCHREKNCESICCGRGHNTQSRVVTRPCQCQVRWCCYVECRQCTQREEVYTCKG

>BOSIN-WNT8A

MGDLLILRVAVGICYVTFSASAWSVNNFLITGPKAYLTYTTSVALGAQSGIEECKFQFAWERWNCPENALQLSTHNRLRS

ATRETSFIHAISSAGVMYTITKNCSMGDFENCGCDESKNGKTGGHGWIWGGCSDNVEFGERISKLFVDSLEKGKDARALM

NLHNNRAGRLAVRATMKRTCKCHGISGSCSIQTCWLQLANFRELGNYLKAKYERALKIEMDKQQLRAGNSAEGHWIPTEA

FLPSAEAELIFLEESPDYCXRNSSLGIYGTEGRECLQNSRNTSRWEQCSCGRLCTECGLQVEERRTEAISSCNCKFQWCC

TVKCEQCRHVVNKYYCTSSPGSAQSRGKGSA

>BOSIN-WNT3A

MPSVAEGIKISIQECQHQFRGRRWNCTTINNSLAIFGPVLDKATRESAFVHAIASAGVAFAVTRSCAEGSAAICGCSSRH

QGSPGEGWKWGGCSEDIEFGGMVSREFADARENRPDARSAMNRHNNEAGRQAIASHMHLKCKCHGLSGSCEVKTCWWSQP

DFRAIGDFLKDKYDSASEMVVEKHRESRGWVETLRPRYTYFKVPTERDLVYYEASPNFCEPNPETGSFGTRDRTCNVSSH

GIDGCDLLCCGRGXNARTEQRREKCHCVFHWCCYVSCQECARVYDVHTCK

>BOSIN-WNT11

MRARPQVCQALLFALALQTGVCYGIKWLALSKTPAALALNQTQHCKQLEGLVSAQVQLCRSNLELMHTIVHAAREVMKAC

RRAFADMRWNCSSIELAPNYLLDLERGTRESAFVYALSAAAISHAIARACTSGDLPGCSCGPVPGEPPGPGNRWGGCADN

LSYGLLMGAKFSDAPMKVKKTGSQANKLMRLHNSEVGRQALRASLEMKCKCHGVSGSCSIRTCWKGLQELRDVAADLKTR

YLSATKVVHRPMGTRKHLVPKDLDIRPVKDSELIYLQSSPDFCMKNEKVGSHGTQDRQCNKTSHGSDSCDLMCCGRGYNP

YTDRVVERCHCKYHWCCYVTCRRCERTVERYVCK

>BOSIN-WNT6

MQPPAPSRLGLLLLLLLSPAHVGGLWWAVGSPLVMDPTSICRKARRLAGRQAELCQAEPEVVAELARGARLGVRECQFQF

RFRRWNCSSHSKAFGRILQQDIRETAFVFAITAAGASHAVTQACSMGELLQCGCQAPRGRAPPRPPGLPGTPGPPGPAGX

XXXXANREWGGCGDDVDFGDEKSRLFMDAQHKRGRGDIRMLVQLHNNEAGRLAVRSHTRTECKCHGLSGSCALRTCWQKL

PPFREVGARLLERFHGASRVMGTNDGKALLPAVRTLKPPGRADLLYAADSPDFCAPNRRTGSPGTRGRACNSSAPDLSGC

DLLCCGRGHRQESVLLEENCLCRFHWCCVVQCHRCRVRKELSLCL

>BOSIN-WNT10A

MGSTHPCPWLRLRPRPQPRPALCALLFFLLLLAASVPRSAPNDILGLRLPQEPVLNANTVCLTLPGLSKRQMEVCVRHPD

VAASAIQGIQIAIHECQHQFRDQRWNCSSLETRNKIPYESPIFSRGFRESAFAYAISAAGVVHAVSNACALGKLRACGCD

ASRRGDEEAFRRKLHRLQLEALQRGKGLSHGVPEHPALPPASPGLQDSWEWGGCSPDVGFGERFSKDFLDSREPHRDIHA

RMRLHNNRVGRQAVMENMRRKCKCHGTSGSCQLKTCWQVTPEFRAVGALLRSRFHRATLIRPHNRNSGQLEPGPAGAPSP

APGLPGPRRRASPADLVYFEKSPDFCEREPRLDSAGTVGRLCNKSSAGPDGCGSMCCGRGHNILRQTRSERCHCRFHWCC

FVVCEECRITEWVSVCK

>BOSIN-WNT3

MEPHLLRLLLGLLLCGTRVLAGYPIWWSLALGQQYTSLGSQPLLCGSIPGLVPKQLRFCRNYIEIMPSVAEGVKLGIQEC

QHQFRGRRWNCTTIDDSLAIFGPVLDKATRESAFVHAIASAGVAFAVTRSCAEGTSTICGCDSHHKGPPGEGWKWGGCSE

DADFGVLVSREFADARENRPDARSAMNKHNNEAGRTTILDHMHLKCKCHGLSGSCEVKTCWWAQPDFRAIGDFLKDKYDS

ASEMVVEKHRESRGWVETLRAKYALFKPPTERDLVYYENSPNFCEPNPETGSFGTRDRTCNVTSHGIDGCDLLCCGRGHN

TRTEKRKEKCHCIFHWCCYVSCQECIRIYDVHTCK

>BOSIN-WNT4

MGLLGEEYRASKALSSMDTQTLYISSTLHVVYLHCDQIERTLWIPDLFLLAVPSLAPQVPHLEETQRPTASEQRLPPRRR

ASASALHPPPRALGSEPGPGHPLLLPEVPCVCTLISAPTMPGNPSGAAELPDLVASQKRGFRFRQSPATVPSCPKHGEAP

SSSRKAGGRYLAKLSSVGSISEEETCEKLKGLIQRQVQMCKRNLEVMDSVRQGAQLAIEECQYQFRNRRWNCSTLDSLPV

FGKVVTQGTREAAFVYAISSAGVAFAVTRACSSGELEKCGCDRTVHGVSPQGFQWSGCSDNIAYGVAFSQSFVDVRERSK

GASSSRALMNLHNNEAGRKAILTHMRVECKCHGVSGSCEVKTCWRAVPPFRQVGHALKEKFXXXXXXXXXXXXXXXXXVP

RNAQFKPHTDEDLVYLEPSPDFCEQDVRSGVLGTRGRTCNKTSKAIDGCELLCCGRGFHTAQVELAERCSCKFHWCCFVK

CRQCQRLVELHTCR

>BOSIN-WNT5A

MKKSIGILSPGVAWGTAGRAMSSKFFLMALAIFLSFAQVVIEANSWWSLGMNNPVQMSEVYIIGAQPLCSQLAGLSQGQK

KLCHLYQDHMQYIGEGAKTGIKECQYQFRHRRWNCSTVDNTSVFGRVMQIGSRETAFTYAVSAAGVVNAMSRACREGELS

TCGCSRAARPKDLPRDWLWGGCGDNIDYGYRFAKEFVDARERERIHAKGSYESARILMNLHNNEAGRRTVYSLADVACKC

HGVSGSCSLKTCWLQLADFRKVGDALKEKYDSAAAMRLNSRGKLVQVNSRFNSPTTQDLVYIDPSPDYCVRNESTGSLGT

QGRLCNKTSEGMDGCELMCCGRGYDQFKTVQTERCHCKFHWCCYVKCKKCTEIVDQFVCK

>BOSIN-WNT7A

MNRKARRCLGHLFLSLGMVYLRIGGFSSVVALGASIICNKIPGLAPRQRAICQSRPDAIIVIGEGSQMGLDECQFQFRNG

RWNCSALGERTVFGKELKVGSREAAFTYAIIAAGVAHAITAACTQGNLSDCGCDKEKQGQYHRDEGWKWGGCSADIRYGI

GFAKVFVDAREIKQNARTLMNLHNNEAGRKILEENMKLECKCHGVSGSCTTKTCWTTLPQFRELGYVLKDKYNEAVHVEP

VRASRNKRPAFLKIKKPLSYRKPMDTELVYIEKSPSYCEEDPATGSVGTQGRACNKTAPQASGCDLMCCGRGYNTHQYAR

VWQCNCKFHWCCYVKCNTCSERTEVYTCK

>BOSIN-WNT8B

MSLSLTXVSLQAYLIYSSSVAAGAQSGIEECKYQFAWDRWNCPERALQLSSHGGLRSANRETAFVHAISSAGVMYTLTRN

CSLGDFDNCGCDDSRNGQLGGQGWLWGGCSDNVGFGEAISKQFVDALETGQDARAAMNLHNNEAGRKAVKGTMKRTCKCH

GVSGSCTTQTCWLQLPEFREVGAHLKEKYHAALKVDLLQGAGNSAAGRGAIADTFRSISTRELVHLEDSPDYCLENKTLG

LLGTEGRECLRRGRALGRWERRSCRRLCGDCGLAVEERRAETVSSCNCKFHWCCAVRCEQCRRRVTKYFCSRADRPRGGA

AHEPGRKP

>BOSIH-WNT10B

MTTGDQRLGSLVWVATPSLQPWRSQSRPCSICPSGPSGPGFDMREEPRPRPPPSGLAGLL

FLALCSRALGNEIQGLKLPGGGEPPLTANTVCLTLSGLSKQQLGLCLRSPDVTASALQGL

HIAVHECQHQLRDQRWNCSALEGGGRLPHHSAILKRGFRESAFSFSMLAAGVMHAVATAC

SLGKLVSCGCGWKGSGEQDRLRAKLLQLQALSRGKSFSHSLPSSGPGSGPSPGPQDTWEW

GGCNHDMDFGEKFSRDFLDSREAPRDIQARMRIHNNRVGRQVVTENLKRKCKCHGTSGSC

QLKTCWRAPPEFRAVGAALRERLDRAIFIDTHNRNSGAFQPRLRPRRLSGELVYFENSPD

FCERDPTVGSPGTQGRACNKTSHQLGSCGSLCCGRGHNVLRQTRVERCNCRFHWCCYVLC

DECKVTEWVNVCK

>BOSIH-WNT7A

MNRKARRCLGHLFLSLGMVYLRIGGFSSVVALGASIICNKIPGLAPRQRAICQSRPDAII

VIGEGSQMGLDECQFQFRNGRWNCSALGERTVFGKELKVGSREAAFTYAIIAAGVAHAIT

AACTQGNLSDCGCDKEKQGQYHRDEGWKWGGCSADIRYGIGFAKVFVDAREIKQNARTLM

NLHNNEAGRKILEENMKLECKCHGVSGSCTTKTCWTTLPQFRELGYVLKDKYNEAVHVEP

VRASRNKRPAFLKIKKPLSYRKPMDTELVYIEKSPSYCEEDPATGSVGTQGRACNKTAPQ

ASGCDLMCCGRGYNTHQYARVWQCNCKFHWCCYVKCNTCSERTEVYTCKTLPPPRDPFSA

INVDRWLSLWDVLLLGRGVE

>BOSIH-WNT9A

MLDGPLLARWLAAAFALTLLLAALRPSAAYFGLTGSEPLTILPLTLEPEAVAQAHYKACD

RLKLERKQRRMCRRDPGVAETLVEAVSMSALECQYQFRFERWNCTLEGRYRASLLKRGFK

ETAFLYAISSAGLTHALAKACSAGRMERCTCDEAPDLENREAWQWGGCGDNLKYSSKFVK

EFLGRRSSKDLRARVDFHNNLVGVKVIKAGVETTCKCHGVSGSCTVRTCWRQLAPFHEVG

KRLKHKYETALKVGSTTNEATGEAGAISPPRGRAAGAGGGDPLPRTPELVHLDDSPSFCV

AGRFSPGTAGRRCHREKNCESICCGRGHNTQSRVVTRPCQCQVRWCCYVECRQCTQREEV

YTCKG

>BOSIH-WNT5B

MPSLPALLALLFACWAPLRATASSWWSLAMSPVQRPEMFIIGAQPVCSQLPGLSAGQRKL

CQLYQEHMAYIGEGARTGIRECQHQFRQRRWNCSTVDDASVFGRVLQIGSRETAFTYAVS

AAGVVNAISRACREGELSTCGCSRAARPKDLPRDWLWGGCGDNVDYGYRFAKEFVDARER

EKNFAKGSEEQGRVLMNLQNNEAGRRAVYKTADVACKCHGVSGSCSLKTCWLQLAEFRKV

GDQLKEKYDSAAAMRITRRGKLELVNSRFKPPTPEDLVYVDPSPDYCLRDESTGSLGTRG

RLCNKTSEGLDGCALMCCGRGYDQFKSVRTERCHCKFHWCCFVRCKKCTQVVDQFVCK

>BOSIH-WNT7B

MAAGRLRRLEDVRLSERQSWFRPIDGRRAGRRARRTQRPRPAPPPGPPASARAAAPAPAR

CEALEAGHGRPLRRGPRARIPSAPFRRPALGRVAETPAPAGPRALPAGPARPPRERRPTM

LLLSPRSALLSVYCPQLFLILSSGSYLALSSVVALGANIICNKIPGLAPRQRAICQSRPD

AIIVIGEGAQMGINECQYQFRFGRWNCSALGEKTVFGQELRVGSREAAFTYAITAAGVAH

AVTAACSQGNLSNCGCDREKQGYYNQAEGWKWGGCSADVRYGIDFSRRFVDAREIKKNAR

RLMNLHNNEAGRKVLEERMKLECKCHGVSGSCTTRTCWTTLPKFREVGHLLKEKYNVAVQ

VEVVRASRLRQPTFLRIKQLRSYQKPMETDLVYIEKSPNYCEEDAATGSVGTQGRLCNRT

SPGADGCDTMCCGRGYNTHQYTKVWQCNCKFHWCCFVKCNTCSERTEVFTCK

>BOSIH-WNT2

MNACLVGIWLWLPLLFTWLSPEVSSSWWYMRATSGSSRVMCDNVPGLVSHQRQLCHRHPD

VMRAIGLGVTEWTMECQHQFRQHRWNCNTLDRDHSLFGRVLLRSSRESAFVYAISSAGVV

FAITRACSQGELKSCSCDPKKKGTAKDNKGTFDWGGCSDNIDYGIKFARAFVDAKERKGK

DARALMNLHNNRAGRKAVKRFLKQECKCHGVSGSCTLRTCWLAMADFRKTGNYLWRKYNG

AIQVVMNQDGTGFTVANKRFKKPTKNDLVYFENSPDYCIRDRDAGSLGTAGRVCNLTSRG

MDSCEVMCCGRGYDTSHITRKTKCECKFHWCCAVRCQDCVEALDVHTCKAPKSPDWAAPT

>BOSIH-WNT11

MRARPQVCQALLFALALQTGVCYGIKWLALSKTPAALALNQTQHCKQLEGLVSAQVQLCR

SNLELMHTIVHAAREVMKACRRAFADMRWNCSSIELAPNYLLDLERGTRESAFVYALSAA

AISHAIARACTSGDLPGCSCGPVPGEPPGPGNRWGGCADNLSYGLLMGAKFSDAPMKVKK

TGSQANKLMRLHNSEVGRQALRASLEMKCKCHGVSGSCSIRTCWKGLQELRDVAADLKTR

YLSATKVVHRPMGTRKHLVPKDLDIRPVKDSELIYLQSSPDFCMKNEKVGSHGTQDRQCN

KTSHGSDSCDLMCCGRGYNPYTDRVVERCHCKYHWCCYVTCRRCERTVERYVCK

>BOSIH-WNT3A

MAPLGYFIFLYGLKQALGNYPIWWSLAVGPQYSSLGTQPILCASIPGLVPKQLRFCRNYV

EIMPSVAEGIKISIQECQHQFRGRRWNCTTINNSLAIFGPVLDKATRESAFVHAIASAGV

AFAVTRSCAEGSAAICGCSSRHQGSPGEGWKWGGCSEDIEFGGMVSREFADARENRPDAR

SAMNRHNNEAGRQAIASHMHLKCKCHGLSGSCEVKTCWWSQPDFRAIGDFLKDKYDSASE

MVVEKHRESRGWVETLRPRYTYFKVPTERDLVYYEASPNFCEPNPETGSFGTRDRTCNVS

SHGIDGCDLLCCGRGHNARTEQRREKCHCVFHWCCYVSCQECARVYDVHTCKR

>BOSIH-WNT8B

RMLVKPSVCIFLFTCVFQLSHTWSVNNFLMTGPKAYLIYSSSVAAGAQSGIEECKYQFAW

DRWNCPERALQLSSHGGLRSANRETAFVHAISSAGVMYTLTRNCSLGDFDNCGCDDSRNG

QLGGQGWLWGGCSDNVGFGEAISKQFVDALETGQDARAAMNLHNNEAGRKAVKGTMKRTC

KCHGVSGSCTTQTCWLQLPEFREVGAHLKEKYHAALKVDLLQGAGNSAAGRGAIADTFRS

ISTRELVHLEDSPDYCLENKTLGLLGTEGRECLRRGRALGRWERRSCRRLCGDCGLAVEE

RRAETVSSCNCKFHWCCAVRCEQCRRRVTKYFCSRADRPRGGAAHEPGRKP

>BOSIH-WNT8A

MLCNLQCLCLVSPSLPLFTPHQGSFHYLIPIHHCLTFSLFGRSVNNFLITGPKAYLTYTT

SVALGAQSGIEECKFQFAWERWNCPENALQLSTHNRLRSATRETSFIHAISSAGVMYTIT

KNCSMGDFENCGCDESKNGKTGGHGWIWGGCSDNVEFGERISKLFVDSLEKGKDARALMN

LHNNRAGRLAVRATMKRTCKCHGISGSCSIQTCWLQLANFRELGNYLKAKYERALKIEMD

KQQLRAGNSAEGHWIPTEAFLPSAEAELIFLEESPDYCTRNSSLGIYGTEGRECLQNSRN

TSRWEQCSCGRLCTECGLQVEERRTEAISSCNCKFQWCCTVKCEQCRHVVNKYYCTSSPG

SAQPRGKGSA

>BOSIH-WNT2B

MLRPGGAEEAAQLPPRRVSAPVPESAPRSTAPDGSRASARLSLACLLLLLLLLTLPARVD

TSWWYIGALGARVICDNIPGLVSRQRQLCQRYPDIMRSVGEGAREWIRECQHQFRHHRWN

CTTLDRDHTVFGRVMLRSSREAAFVYAISSAGVVHAITRACSQGELSVCSCDPYTRGRHH

DQRGDFDWGGCSDNIHYGVRFAKAFVDAKEKRLKDARALMNLHNNRCGRTAVRRFLKLEC

KCHGVSGSCTLRTCWRALSDFRRTGDYLRRRYDGAVQVTATQDGANFTAARQGYRRATRT

DLVYFDNSPDYCVLDKAAGSLGTAGRVCSKTSKGTDGCEIMCCGRGYDTTRVTRVTQCEC

KFHWCCAVRCKECRNTVDVHTCKAPKKAEWLDQT

>BOSIH-WNT1

MGHWALLPCWVSAALLLALAALPAALAANSSGRWWGIVNVASSTNLLTDSKSLQLVLEPS

LQLLSRKQRRLIRQNPGILHSVSGGLQSAVRECKWQFRNRRWNCPTASGPHLFGKIVNRG

CRETAFIFAITSAGVTHSVARSCSEGSIESCTCDYRRRGPGGPDWHWGGCSDNIDFGRLF

GREFVDSGEKGRDLRFLMNLHNNEAGRTTVFSEMRQECKCHGMSGSCTVRTCWMRLPTLR

AVGDVLRDRFDGASRVLYGNRGNNRASRAELLRLEPEDPAHKPPSPHDLVYFEKSPNFCT

YSGRLGTAGTAGRACNSSSPALDGCELLCCGRGHRTRTQRVTERCNCTFHWCCHVSCRNC

THTRVLHECL

>BOSIH-WNT16

MDRAALLGLSRLCALWAAVLALFPCGAQGNWMWLGIASFGVPEKLGCANLPLNSRQKELC

KRKPYLLPSIREGARLGIQECRSQFRHERWNCLVAAASAPGTSPLFGYELSSGTKETAFI

YAVMAAGLVHSVTRSCSAGNMTECSCDTTLQNGGSASEGWHWGGCSDDVQYGMWFSRKFL

DFPIKNTTAKESKVLLAMNLHNNEAGRQAVAKLMSLDCRCHGVSGSCAVKTCWKTMSSFE

KIGHLLKDKYENSVQISDKIKRKMHRREKDQRKIPIRKDDLLYVNKSPNYCVEDKKLGIP

GTQGRECNRTSEGADGCNLLCCGRGYNTHVVRHVERCECKFIWCCYVRCRRCESMTDVHT

CK

>BOSIH-WNT10A

MGSTHPCPWLRLRPRPQPRPALCALLFFLLLLAASVPRSAPNDILGLRLPQEPVLNANTV

CLTLPGLSKRQMEVCVRHPDVAASAIQGIQIAIHECQHQFRDQRWNCSSLETRNKIPYES

PIFSRGFRESAFAYAISAAGVVHAVSNACALGKLRACGCDASRRGDEEAFRRKLHRLQLE

ALQRGKGLSHGVPEHPALPPASPGLQDSWEWGGCSPDVGFGERFSKDFLDSREPHRDIHA

RMRLHNNRVGRQAVMENMRRKCKCHGTSGSCQLKTCWQVTPEFRAVGALLRSRFHRATLI

RPHNRNSGQLEPGPAGAPSPAPGLPGPRRRASPADLVYFEKSPDFCEREPRLDSAGTVGR

LCNKSSAGPDGCGSMCCGRGHNILRQTRSERCHCRFHWCCFVVCEECRITEWVSVCK

>BOSIH-WNT6

MQPPAPSRLGLLLLLLLSPAHVGGLWWAVGSPLVMDPTSICRKARRLAGRQAELCQAEPE

VVAELARGARLGVRECQFQFRFRRWNCSSHSKAFGRILQQDIRETAFVFAITAAGASHAV

TQACSMGELLQCGCQAPRGRAPPRPPGLPGTPGPPGPAGSPDGSAAWEWGGCGDDVDFGD

EKSRLFMDAQHKRGRGDIRMLVQLHNNEAGRLAVRSHTRTECKCHGLSGSCALRTCWQKL

PPFREVGARLLERFHGASRVMGTNDGKALLPAVRTLKPPGRADLLYAADSPDFCAPNWRT

GSPGTRGRACNSSAPDLSGCDLLCCGRGHRQESVLLEENCLCRFHWCCVVQCHRCRVRKE

LSLCL

>BOSIH-WNT3

MEPHLLRLLLGLLLCGTRVLAGYPIWWSLALGQQYTSLGSQPLLCGSIPGLVPKQLRFCR

NYIEIMPSVAEGVKLGIQECQHQFRGRRWNCTTIDDSLAIFGPVLDKATRESAFVHAIAS

AGVAFAVTRSCAEGTSTICGCDSHHKGPPGEGWKWGGCSEDADFGVLVSREFADARENRP

DARSAMNKHNNEAGRTTILDHMHLKCKCHGLSGSCEVKTCWWAQPDFRAIGDFLKDKYDS

ASEMVVEKHRESRGWVETLRAKYALFKPPTERDLVYYENSPNFCEPNPETGSFGTRDRTC

NVTSHGIDGCDLLCCGRGHNTRTEKRKEKCHCIFHWCCYVSCQECIRIYDVHTCK

>BOSIH-WNT9B

MRPPPALALAALCLLALPAAAAAAYFGLTGREVLTPFPGLGTAAPAQGGAHLKQCDLLKL

SRRQKQLCRREPGLAETLQDAAHLSLLECQFQFRHERWNCSLEGRTGLLKRGFKETAFLY

AVSAAALTHTLARACSAGRMERCTCDDSPGLESRQAWQWGVCGDNLKYSTKFLNNFLGPK

RGSKDLRARADAHNTHVGIKAVKSGLRTTCKCHGVSGSCAVRTCWKQLSPFRDTGQVLKL

RYDSAVKVSSASNEALGRLELWAPARPGSPSKGPAPRPGDLVYMEDSPSFCRPSKYSPGT

GGRVCSREASCSSLCCGRGYDTQSRLTAFSCHCQVQWCCYVECQQCVREELVYTCKH

>BOSIH-WNT5A

MKKSIGILSPGVAWGTAGRAMSSKFFLMALAIFLSFAQVVIEANSWWSLGMNNPVQMSEV

YIIGAQPLCSQLAGLSQGQKKLCHLYQDHMQYIGEGAKTGIKECQYQFRHRRWNCSTVDN

TSVFGRVMQIGSRETAFTYAVSAAGVVNAMSRACREGELSTCGCSRAARPKDLPRDWLWG

GCGDNIDYGYRFAKEFVDARERERIHAKGSYESARILMNLHNNEAGRRTVYSLADVACKC

HGVSGSCSLKTCWLQLADFRKVGDALKEKYDSAAAMRLNSRGKLVQVNSRFNSPTTQDLV

YIDPSPDYCVRNESTGSLGTQGRLCNKTSEGMDGCELMCCGRGYDQFKTVQTERCHCKFH

WCCYVKCKKCTEIVDQFVCK

>BOSIH-WNT4

MSPRWCLRSLRLLVFAVFSAAASNWLYLAKLSSVGSISEEETCEKLKGLIQRQVQMCKRN

LEVMDSVRRGAQLAIEECQYQFRNRRWNCSTLDSLPVFGKVVTQGTREAAFVYAISSAGV

AFAVTRACSSGELEKCGCDRTVHGVSPQGFQWSGCSDNIAYGVAFSQSFVDVRERSKGAS

SSRALMNLHNNEAGRKAILTHMRVECKCHGVSGSCEVKTCWRAVPPFRQVGHALKEKFDG

ATEVEPRRVGSSRALVPRNAQFKPHTDEDLVYLEPSPDFCEQDVRSGVLGTRGRTCNKTS

KAIDGCELLCCGRGFHTAQVELAERCSCKFHWCCFVKCRQCQRLVELHTCR

>BISON-WNT11

MRARPQVCQALLFALALQTGVCYGIKWLALSKTPAALALNQTQHCKQLEGLVSAQVQLCR

SNLELMHTIVHAAREVMKACRRAFADMRWNCSSIELAPNYLLDLERGTRESAFVYALSAA

AISHAIARACTSGDLPGCSCGPVPGEPPGPGNRWGGCADNLSYGLLMGAKFSDAPMKVKK

TGSQANKLMRLHNSEVGRQALRASLEMKCKCHGVSGSCSIRTCWKGLQELRDVAADLKTR

YLSATKVVHRPMGTRKHLVPKDLDIRPVKDSELIYLQSSPDFCMKNEKVGSHGTQDRQCN

KTSHGSDSCDLMCCGRGYNPYTDRVVERCHCKYHWCCYVTCRRCERTVERYVCK

>BISON-WNT3

MEPHLLRLLLGLLLCGTRVLAGYPIWWSLALGQQYTSLGSQPLLCGSIPGLVPKQLRFCR

NYIEIMPSVAEGVKLGIQECQHQFRGRRWNCTTIDDSLAIFGPVLDKATRESAFVHAIAS

AGVAFAVTRSCAEGTSTICGCDSHHKGPPGEGWKWGGCSEDADFGVLVSREFADARENRP

DARSAMNKHNNEAGRTTILDHMHLKCKCHGLSGSCEVKTCWWAQPDFRAIGDFLKDKYDS

ASEMVVEKHRESRGWVETLRAKYALFKPPTERDLVYYENSPNFCEPNPETGSFGTRDRTC

NVTSHGIDGCDLLCCGRGHNTRTEKRKEKCHCIFHWCCYVSCQECIRIYDVHTCK

>BISON-WNT5B

MPSLPALLALLLACWAPLRATASSWWSLAMSPVQRPEMFIIGAQPVCSQLPGLSAGQRKL

CQLYQEHMAYIGEGARTGIRECQHQFRQRRWNCSTVDDASVFGRVLQIGSRETAFTYAVS

AAGVVNAISRACREGELSTCGCSRAARPKDLPRDWLWGGCGDNVDYGYRFAKEFVDARER

EKNFAKGSEEQGRVLMNLQNNEAGRRAVYKTADVACKCHGVSGSCSLKTCWLQLAEFRKV

GDQLKEKYDSAAAMRITRRGKLELVNSRFKPPTPEDLVYVDPSPDYCLRDESTGSLGTRG

RLCNKTSEGLDGCALMCCGRGYDQFKSVRTERCHCKFHWCCFVRCKKCTRVVDQFVCK

>BISON-WNT10A

MGSTHPCPWLRLRPRPQPRPALCALLFFLLLLAASVPRSAPNDILGLRLPQEPVLNANTV

CLTLPGLSKRQMEVCVRHPDVAASAIQGIQIAIHECQHQFRDQRWNCSSLETRNKIPYES

PIFSRGFRESAFAYAISAAGVVHAVSNACALGKLRACGCDASRRGDEEAFRRKLHRLQLE

ALQRGKGLSHGVPEHPALPPASPGLQDSWEWGGCSPDVGFGERFSKDFLDSREPHRDIHA

RMRLHNNRVGRQAVMENMRRKCKCHGTSGSCQLKTCWQVTPEFRAGGALLRSRFHRATLI

RPHNRNSGXXXXXXXXXXXXXXXXXXXXXXXXXXXXVYFEKSPDFCEREPRLDSAGTVGR

LCNKSSAGPDGCGSMCCGRGHNILRQTRSERCHCRFHWGCFGVCEECRITEWVSVCK

>BISON-WNT6

MLSALRPPPLRVTTMVCCSLLPRAVGSPLVMDPTSICRKARRLAGRQAELCQAEPEVVAE

LARGARLGVRECQFQFRFRRWNCSSHSKAFGRILQQDIRETAFVFAITAAGASHAVTQAC

SMGELLQCGCQAPRGRAPPRPPGLPGTPGPPGPAGSPDGSAAWEWGGCGDDVDFGDEKSR

LFMDAQHKRGRGDIRMLVQLHNNEAGRLAVRSHTRTECKCHGLSGSCALRTCWQKLPPFR

EVGARLLERFHGASRVMGTNDGKALLPAVRTLKPPGRADLLYAADSPDFCAPNRRTGSPG

TRGRACNSSAPDLSGCDLLCCGRGHRQESVLLEENCLCRFHWCCVVQCHRCRVRKELSLC

L

>BISON-WNT2B

MLRPGGAEEAAQLPPRRVSAPVPESAPRPTVPDGSRASARLSLACLLLLLLLLTLPARVD

TSWWYIGALGARVICDNIPGLVSRQRQLCQRYPDIMRSVGEGAREWIRECQHQFRHHRWN

CTTLDRDHTVFGRVMLRSSREAAFVYAISSAGVVHAITRACSQGELSVCSCDPYTRGRHH

DQRGDFDWGGCSDNIHYGVRFAKAFVDAKEKRLKDARALMNLHNNRCGRTAVRRFLKLEC

KCHGVSGSCTLRTCWRALSDFRRTGDYLRRRYDGAVQVTATQDGANFTAARQGYRRATRT

DLVYFDNSPDYCVLDKAAGSLGTAGRVCSKTSKGTDGCEIMCCGRGYDTTRVTRVTQCEC

KFHWCCAVRCKECRNTVDVHTCKAPKKAEWLDQT

>BISON-WNT7A

XXXXXXXXLFLSLGMVYLRIGGFSSVVALGASIICNKIPGLAPRQRAICQSRPDAIIVIG

EGSQMGLDECQFQFRNGRWNCSALGERTVFGKELKVGSREAAFTYAIIAAGVAHAITAAC

TQGNLSDCGCDKEKQGQYHRDEGWKWGGCSADIRYGIGFAKVFVDAREIKQNARTLMNLH

NNEAGRKILEENMKLECKCHGVSGSCTTKTCWTTLPQFRELGYVLKDKYNEAVHVEPVRA

SRNKRPAFLKIKKPLSYRKPMDTELVYIEKSPSYCEEDPATGSVGTQGRACNKTAPQXXX

XXXXXXXXXXXXXXXXXXXXXXXXXXXXXXXXXXXXXXXXXXXXXXX

>BISON-WNT4A

ESPLSSPSTIRLLLAYLAKLSSVGSISEEETCEKLKGLIQRQVQMCKRNLEVMDSVRRGA

QLAIEECQYQFRNRRWNCSTLDSLPVFGKVVTQGTREAAFVYAISSAGVAFAVTRACSSG

ELEKCGCDRTVHGVSPQGFQWSGCSDNIAYGVAFSQSFVDVRERSKGASSSRALMNLHNN

EAGRKAILTHMRVECKCHGVSGSCEVKTCWRAVPPFRQVGHALKEKFDGATEVEPRRVGS

SRALVPRNAQFKPHTDEDLVYLEPSPDFCEQDVRSGVLGTRGRTCNKTSKAIDGCELLCC

GRGFHTAQVELAERCSCKFHWCCFVKCRQCQRLVELHTCR

>BISON-WNT9A

XXXXXXXXXXXXXXXXXXXXXXXLRPSAAYFGLTGSEPLTILPLTLEPEAVAQAHYKACD

RLKLERKQRRMCRRDPGVAETLVEAVSMSALECQYQFRFERWNCTLEGRYRASLLKRGFK

ETAFLYAISSAGLTHALAKACSAGRMERCTCDEAPDLENREAWQWGGCGDNLKYSSKFVK

EFLGRRSSKDLRARVDFHNNLVGVKVIKAGVETTCKCHGVSGSCTVRTCWRQLAPFHEVG

KRLKHKYETALKVGSTTNEATGEAGAISPPRGRAAGAGGGDPLPRTPELVHLDDSPSFCV

AGRFSPGTAGRRCHREKNCESICCGRGHNTQSRVVTRPCQCQVRWCCYVECRQCTQREEV

YTCKG

>BISON-WNT3A

MELPPRGEGSIETQEPSRALLTRAHHPTNTDGTSPTNQASLSSSPRSLAVGPQYSSLGTQ

PILCASIPGLVPKQLRFCRNYVEIMPSVGGHPQSRGRRWNCTTINNSLAIFGPVLDKATR

ESAFVHAIASAGVAFAVTRSCAEGSAAICGCSSRHQGSPGEGWKWGGCSEDIEFGGMVSR

EFADARENRPDARSAMNRHNNEAGRQAIASHMHLKCKCHGLSGSCEVKTCWWSQPDFRAI

GDFLKDKYDSASEMVVEKHRESRGWVETLRPRYTYFKVPTERDLVYYEASPNFCEPNPET

GSFGTRDRTCNVSSHGIDGCDLLCCGRGHNARTEQRREKCHCVFHWCCYVSCQECARVYD

VHTCK

>BISON-WNT5A

MKKSIGILSPGVAWGTAGRAMSSKFFLMALAIFLSFAQVVIEANSWWSLGMNNPVQMSEV

YIIGAQPLCSQLAGLSQGQKKLCHLYQDHMQYIGEGAKTGIKECQYQFRHRRWNCSTVDN

TSVFGRVMQIGSRETAFTYAVSAAGVVNAMSRACREGELSTCGCSRAARPKDLPRDWLWG

GCGDNIDYGYRFAKEFVDARXXXXXXXXXXXXXXXXXXXXHNNEAGRRTVYSLADVACKC

HGVSGSCSLKTCWLQLADFRKVGDALKEKYDSAAAMRLNSRGKLVQVNSRFNSPTTQDLV

YIDPSPDYCVRNESTGSLGTQGRLCNKTSEGMDGCELMCCGRGYDQFKTVQTERCHCKFH

WCCYVKCKKCTEIVDQFVCK

>BISON-WNT16

MDRAALLGLSRLCALWAAVLALFPCGAQGNWMWLGIASFGVPEKLGCANLPLNSRQKELC

KRKPYLLPSIREGARLGIQECRSQFRHERWNCLVAAASAPGTSPLFGYELSSGTKETAFI

YAVMAAGLVHSVTRSCSAGNMTECSCDTTLQNGGSASEGWHWGGCSDDVQYGMWFSRKFL

DFPIKNTTAKESKVLLAMNLHNNEAGRQAVAKLMSLDCRCHGVSGSCAVKTCWKTMSSFE

KIGHLLKDKYENSVQISDKINRKMHRREKDQRKIPIRKDDLLYVNKSPNYCVEDKKLGIP

GTQGRECNRTSEGADGCNLLCCGRGYNTHVVRHVERCECKFIWCCYVRCRRCESMTDVHT

CK

>BISON-WNT8A

MLCNLQCLCLVSPSLPLFTPHQGSFHYLIPIHHCLTFSLFGRSVNNFLITGPKAYLTYTT

SVALGAQSGIEECKFQFAWERWNCPENALQLSTHNRLRSATRETSFIHAISSAGVMYTIT

KNCSMGDFENCGCDESKNGKTGGHGWIWGGCSDNVEFGERISKLFVDSLEKGKDARALMN

LHNNRAGRLAVRATMKRTCKCHGISGSCSIQTCWLQLANFRELGNYLKAKYERALKIEMD

KQQLRAGNSAEGHWIPTEAFLPSAEAELIFLEESPDYCTRNSSLGIYGTEGRECLQNSRN

TSRWEQCSCGRLCTECGLQVEERRTEAISSCNCKFQWCCTVKCEQCRHVVNKYYCTRSPG

SAQSWGKGSA

>BISON-WNT9B

FSLPFLLLLLSLVTGLPSLAPFSLTGREVLTPFPGLGTAAPAQGGAHLKQCDLLKLSRRQ

KQLCRREPGLAETLQDAAHLSLLECQFQFRHERWNCSLEGRTGLLKRGFKETAFLYAVSA

AALTHTLARACSAGRMERCTCDDSPGLESRQAWQWGVCGDNLKYSTKFLNNFLGPKRGSK

DLRARADAHNTHVGIKAVKSGLRTTCKCHGVSGSCAVRTCWKQLSPFRDTGQVLKLRYDS

AVKVSSASNEALGRLELWAPARPGSPSKGPAPRPGDLVYMEDSPSFCRPSKYSPGTGGRV

CSRESRLTAFSCHCQVQWCCYVECQQCVREELVYTCKH

>BISON-WNT8B

RMLVKPSVCIFLFTCVFQLSHTWSVNNFLMTGPKAYLIYSSSVAAGAQSGIEECKYQFAW

DRWNCPERALQLSSHGGLRSANRETAFVHAISSAGVMYTLTRNCSLGDFDNCGCDDSRNG

QLGGQGWLWGGCRDNVGFGEAISKQFVDALETGQDARAAMNLHNNEAGRKAVKGTMKRTC

KCHGVSGSCTTQTCWLQLPEFREVGAHLKEKYHAALKVDLLQGAGNSAAGRGAIADTFRS

ISTRELVHLEDSPDYCLENKTLGLLGTEGRECLRRGRALGRWERRSCRRLCGDCGLAVEE

RRAETVSSCNCKFHWCCAVRCEQCRRRVTKYFCSRADRPRGGAAHEPGRKP

>BISON-WNT1

ALLPCWVSAALLLALAALPAALAANSSGRWWGIVNVASSTNLLTDSKSLQLVLEPSLQLL

SRKQRRLIRQNPGILHSVSGGLQSAVRECKWQFRNRRWNCPTASGPHLFGKIVNRGCRET

AFIFAITSAGVTHSVARSCSEGSIESCTCDYRRRGPGGPDWHWGGCSDNIDFGRLFGREF

VDSGEKGRDLRFLMNLHNNEAGRTTVFSEMRQECKCHGMSGSCTVRTCWMRLPTLRAVGD

VLRDRFDGASRVLYGNRGNNRASQAELLRLEPEDPAHKPPSPHDLVYFEKSPNFCTYSGR

LGTAGTAGRACNSSSPALDGCELLCCGRGHRTRTQRVTERCNCTFHWCCHVSCRNCTHTR

VLHECL

>BISON-WNT10B

MREEPRPRPPPSGLAGLLFLALCSRALGNEIQGLKLPGGGEPPLTAHTGXXXXXXXXXXX

XXXXXXXXXXXXXXXXXXXXXXXXXXXXXXXXXXXXPGGGAPLPHHSAILKRGFRESAFS

FSMLAAGVMHAVATACSLGKLVSCGCGWKGSGEQDRLRAKLLQLQALSRGKSFSHSLPSS

GPGSGPSPGPQDTWEWGGCNHDMDFGEKFSRDFLDSREAPRDIQARMRIHNNRVGRQVVT

ENLKRKCKCHGTSGSCQLKTCWRAPPEFRAVGAALRERLDRAIFIDTHNRNSGAFQPRLR

PRRLSGELVYFENSPDFCERDPTVGSPGTQGRACNKTSHQLGSCGSLCCGRGHNVLRQTR

VERCNCRFHWCCYVLCDECKVTEWVNVCK

>BISON-WNT2

MNACLVGIWLWLPLLFTWLSPEVSSSWWYMRATSGSSRVMCDNVPGLVSHQRQLCHRHPD

VMRAIGLGVTEWTMECQHQFRQHRWNCNTLDRDHSLFGRVLLRSSRESAFVYAISSAGVV

FAITRACSQGELKSCSCDPKKKGTAKDNKGTFDWGGCSDNIDYGIKFARAFVDAKERKGK

DARALMNLHNNRAGRKAVKRFLKQECKCHGVSGSCTLRTCWLAMADFRKTGNYLWRKYNG

AIQVVMNQDGTGFTVANKRFKKPTKNDLVYFENSPDYCIRDRDAGSLGTAGRVCNLTSRG

MDSCEVMCCGRGYDTSHITRKTKCECKFHWCCAVRCQDCVEALDVHTCKAPKSPEWAAPT

>BOSGR-WNT10A

MGSTHPCPWLRLRPRPQPRPALCALLFFLLLLAASVPRSAPNDILGLRLPQEPVLNANTV

CLTLPGLSKRQMEVCVRHPDVAASAIQGIQIAIHECQHQFRDQRWNCSSLETRNKIPYES

PIFSRGFRESAFAYAISAAGVVHAVSNACALGKLRACGCDASRRGDEEAFRRKLHRLQLE

ALQRGKGLSHGVPEHPALPPASPGLQDSWEWGGCSPDVGFGERFSKDFLDSREPHRDIHA

RMRLHNNRVGRQAVMENMRRKCKCHGTSGSCQLKTCWQVTPEFRAVGALLRSRFHRATLI

RPHNRNSGQLEPGPAGAPSPAPGLPGPRRRASPADLVYFEKSPDFCEREPRLDSAGTVGR

LCNKSSAGPDGCGSMCCGRGHNILRQTRSERCHCRFHWCCFVVCEECRITEWVSVCK

>BOSGR-WNT6

MQPPAPSRLGLLLLLLLSPAHVGGLWWAVGSPLVMDPTSICRKARRLAGRQAELCQAEPE

VVAELARGARLGVRECQFQFRFRRWNCSSHSKAFGRILQQDIRETAFVFAITAAGASHAV

TQACSMGELLQCGCQAPRGRAPPRPPGLPGTPGPPGPAGSPDGSAAWEWGGCGDDVDFGD

EKSRLFMDAQHKRGRGDIRMLVQLHNNEAGRLAVRSHTRTECKCHGLSGSCALRTCWQKL

PPFREVGARLLERFHGASRVMGTNDGKALLPAVRTLKPPGRADLLYAADSPDFCAPNRRT

GSPGTRGRACNSSAPDLSGCDLLCCGRGHRQESVLLEENCLCRFHWCCVVQCHRCRVRKE

LSLCL

>BOSGR-WNT10B

MREEPRPRPPPLGLAGLLFLALCSRALGNEIQGLKLPGGGEPPLTANTVCLTLSGLSKQQ

LGLCLRSPDVTASALQGLHIAVHECQHQLRDQRWNCSALEGGGRLPHHSAILKRGFRESA

FSFSMLAAGVMHAVATACSLGKLVSCGCGWKGSGEQDRLRAKLLQLQALSRGKSFSHSLP

SSGPGSGPSPGPQDTWEWGGCNHDMDFGEKFSRDFLDSREAPRDIQARMRIHNNRVGRQV

VTENLKRKCKCHGTSGSCQLKTCWRAPPEFRAVGAALRERLDRAIFIDTHNRNSGAFQPR

LRPRRLSGELVYFENSPDFCERDPTVGSPGTQGRACNKTSHQLGSCGSLCCGRGHNVLRQ

TRVERCNCRFHWCCYVLCDECKVTEWVNVCK

>BOSGR-WNT1

MGHWALLPCWVSAALLLALAALPAALAANSSGRWWGIVNVASSTNLLTDSKSLQLVLEPS

LQLLSRKQRRLIRQNPGILHSVSGGLQSAVRECKWQFRNRRWNCPTASGPHLFGKIVNRG

CRETAFIFAITSAGVTHSVARSCSEGSIESCTCDYRRRGPGGPDWHWGGCSDNIDFGRLF

GREFVDSGEKGRDLRFLMNLHNNEAGRTTVFSEMRQECKCHGMSGSCTVRTCWMRLPTLR

AVGDVLRDRFDGASRVLYGNRGNNRASRAELLRLEPEDPAHKPPSPHDLVYFEKSPNFCT

YSGRLGTAGTAGRACNSSSPALDGCELLCCGRGHRTRTQRVTERCNCTFHWCCHVSCRNC

THTRVLHECL

>BOSGR-WNT7B

MAAGRLRRLEDVRLSERQSWFRPIDGRRAGRRPRRTHRPRPAPPPGPPASARAAAPAPAR

CEALEAGHGRPLRRGPRARVPSAPFRRPALGRVAETPAPAGPRALPAGPARPPRERRPTM

LLLSPRSALLSVYCPQLFLILSSGSYLALSSVVALGANIICNKIPGLAPRQRAICQSRPD

AIIVIGEGAQMGINECQYQFRFGRWNCSALGEKTVFGQELRVGSREAAFTYAITAAGVAH

AVTAACSQGNLSNCGCDREKQGYYNQAEGWKWGGCSADVRYGIDFSRRFVDAREIKKNAR

RLMNLHNNEAGRKVLEERMKLECKCHGVSGSCTTKTCWTTLPKFREVGHLLKEKYNVAVQ

VEVVRASRLRQPTFLRIKQLRSYQKPMETDLVYIEKSPNYCEEDAATGSVGTQGRLCNRT

SPGADGCDTMCCGRGYNTHQYTKVWQCNCKFHWCCFVKCNTCSERTEVFTCK

>BOSGR-WNT8A

MLCNLQCLCLVSPSLPLFTPHQGSFHYLIPIHHCLTFSLFGRSVNNFLITGPKAYLTYTT

SVALGAQSGIEECKFQFAWERWNCPENALQLSTHNRLRSATRETSFIHAISSAGIMYTIT

KNCSMGDFENCGCDESKNGKTGGHGWIWGGCSDNVEFGERISKLFVDSLEKGKDARALMN

LHNNRAGRLAVRATMKRTCKCHGISGSCSIQTCWLQLANFRELGNYLKAKYERALKIEMD

KQQLRAGNSAEGHWIPTEAFLPSAEAELIFLEESPDYCTRNSSLGIYGTEGRECLQNSRN

TSRWEQCSCGRLCTECGLQVEERRTEAISSCNCKFQWCCTVKCEQCRHVVNKYYCTRSPG

SAQSRGKGSA

>BOSGR-WNT9A

MLDGPLLARWLAAAFALTLLLAALRPSAAYFGLTGSEPLTILPLTLEPEAVAQAHYKACD

RLKLERKQRRMCRRDPGVAETLVEAVSMSALECQYQFRFERWNCTLEGRYRASLLKRGFK

ETAFLYAISSAGLTHALAKACSAGRMERCTCDEAPDLENREAWQWGGCGDNLKYSSKFVK

EFLGRRSSKDLRARVDFHNNLVGVKVIKAGVETTCKCHGVSGSCTVRTCWRQLAPFHEVG

KRLKHKYETALKVGSTTNEATGEGAISPPRGRAAGAGGGDPLPRTPELVHLDDSPSFCVA

GRFSPGTAGRRCHREKNCESICCGRGHNTQSRVVTRPCQCQVRWCCYVECRQCTQREEVY

TCKG

>BOSGR-WNT3A

MAPLGYFIFLYGLKQALGNYPIWWSLAVGPQYSSLGTQPILCASIPGLVPKQLRFCRNYV

EIMPSVAEGIKISIQECQHQFRGRRWNCTTINNSLAIFGPVLDKATRESAFVHAIASAGV

AFAVTRSCAEGSAAICGCSSRHQGSPGEGWKWGGCSEDIEFGGMVSREFADARENRPDAR

SAMNRHNNEAGRQAIASHMHLKCKCHGLSGSCEVKTCWWSQPDFRAIGDFLKDKYDSASE

MVVEKHRESRGWVETLRPRYTYFKVPTERDLVYYEASPNFCEPNPETGSFGTRDRTCNVS

SHGIDGCDLLCCGRGHNARTEQRREKCHCVFHWCCYVSCQECARVYDVHTCK

>BOSGR-WNT4

MSPRWCLRSLRLLVFAVFSAAASNWLYLAKLSSVGSISEEETCEKLKGLIQRQVQMCKRN

LEVMDSVRRGAQLAIEECQYQFRNRRWNCSTLDSLPVFGKVVTQGTREAAFVYAISSAGV

AFAVTRACSSGELEKCGCDRTVHGVSPQGFQWSGCSDNIAYGVAFSQSFVDVRERSKGAS

SSRALMNLHNNEAGRKAILTHMRVECKCHGVSGSCEVKTCWRAVPPFRQVGHALKEKFDG

ATEVEPRRVGSSRALVPRNAQFKPHTDEDLVYLEPSPDFCEQDVRSGVLGTRGRTCNKTS

KAIDGCELLCCGRGFHTAQVELAERCSCKFHWCCFVKCRQCQRLVELHTCR

>BOSGR-WNT2

MRATSGSSRVMCDNVPGLVSHQRQLCHRHPDVMRAIGLGVTEWTMECQHQFRQHRWNCNT

LDRDHSLFGRVLLRSSRESAFVYAISSAGVVFAITRACSQGELKSCSCDPKKKGTAKDNK

GTFDWGGCSDNIDYGIKFARAFVDAKERKGKDARALMNLHNNRAGRKAVKRFLKQECKCH

GVSGSCTLRTCWLAMADFRKTGNYLWRKYNGAIQVVMNQDGTGFTVANKRFKKPTKNDLV

YFENSPDYCIRDRDAGKFKTLYSKYLILPRISTPQFKCSTVFSTCQSSKCSLLSSGSKLR

FFSIHILSAQKAFLSNWRNSLIMCRFWRNGTQQECTCQKVKMCRLAKVLSLGRTLTGGFM

STTYRSPASSL

>BOSGR-WNT2B

MLRPGGAEEAAQLPPRRVSAPVPESAPRPTAPDGSRASARLSLACLLLLLLLLTLPARVD

TSWWYIGALGARVICDNIPGLVSRQRQLCQRYPDIMRSVGEGAREWIRECQHQFRHHRWN

CTTLDRDHTVFGRVMLRSSREAAFVYAISSAGVVHAITRACSQGELSVCSCDPYTRGRHH

DQRGDFDWGGCSDNIHYGVRFAKAFVDAKEKRLKDARALMNLHNNRCGRTAVRRFLKLEC

KCHGVSGSCTLRTCWRALSDFRRTGDYLRRRYDGAVQVTATQDGANFTAARQGYRRATRT

DLVYFDNSPDYCVLDKAAGSLGTAGRVCSKTSKGTDGCEIMCCGRGYDTTRVTRVTQCEC

KFHWCCAVQCKECRNTVDVHTCKAPRRQSGWIRPEHKNTSLVPPLQTS

>BOSGR-WNT11

MNEGLQPRKGQGCAQGHTRVGGRAIPLLGTAVLGLLLSQLLPVKAPEPLRKAGAMQWVEA

AGSLLGLTFLFTGCFLALCSRPDFLFTGCFLATCRALSKTPAALALNQTQHCKQLEGLVS

AQVQLCRSNLELMHTIVHAAREVMKACRRAFADMRWNCSSIELAPNYLLDLERGTRESAF

VYALSAAAISHAIARACTSGDLPGCSCGPVPGEPPGPGNRWGGCADNLSYGLLMGAKFSD

APMKVKKTGSQANKLMRLHNSEVGRQALRASLEMKCKCHGVSGSCSIRTCWKGLQELRDV

AADLKTRYLSATKVVHRPMGTRKHLVPKDLDIRPVKDSELIYLQSSPDFCMKNEKVGSHG

TQDRQCNKTSHGSDSCDLMCCGRGYNPYTDRVVERCHCKYHWCCYVTCRRCRAWTEACAL

PPLGSRQEQKAGPSGKESQDIKGDRQD

>BOSGR-WNT7A

MNRKARRCLGHLFLSLGMVYLRIGGFSSVVALGASIICNKIPGLAPRQRAICQSRPDAII

VIGEGSQMGLDECQFQFRNGRWNCSALGERTVFGKELKVGSREAAFTYAIIAAGVAHAIT

AACTQGNLSDCGCDKEKQGQYHRDEGWKWGGCSADIRYGIGFAKVFVDAREIKQNARTLM

NLHNNEAGRKILEENMKLECKCHGVSGSCTTKTCWTTLPQFRELGYVLKDKYNEAVHVEP

VRASRNKRPAFLKIKKPLSYRKPMDTELVYIEKSPSYCEEDPATGSVGTQGRACNKTAPQ

ASGCDLMCCGRGYNTHQYARVWQCNCKFHWCCYVKCNTCSERTEVYTCK

>BOSGR-WNT5B

MLVWAVSLRTVSCPILSTLPWKPVQLVLPQLHPQCLAGGGGCPVSHIPIPALWIQAKRSE

EPRLGSRRSPGSSPALGGAGRRRTMPSLPALLALLFACWAPLRATASSWWSLAMSPVQRP

EMFIIGAQPVCSQLPGLSAGQRKLCQLYQEHMAYIGEGARTGIRECQHQFRQRRWNCSTV

DDASVFGRVLQIGSRETAFTYAVSAAGVVNAISRACREGELSTCGCSRAARPKDLPRDWL

WGGCGDNVDYGYRFAKEFVDAREREKNFAKGSEEQGRVLMNLQNNEAGRRAVYKTADVAC

KCHGVSGSCSLKTCWLQLAEFRKVGDQLKEKYDSAAAMRITRRGKLELVNSRFKPPTPED

LVYVDPSPDYCLRDESTGSLGTRGRLCNKTSEGLDGCALMCCGRGYDQFKSVRTERCHCK

FHWCCFVRCKKCTQVVDQFVCK

>BOSGR-WNT3

MPFPEPDSWAGREGRVIDRLNYRLISYLRPRALIGCSLTSSNPAAPRWALEGGSCGWRCA

SDKPESHFQSQVDFVPTTGGRRSPSSWSRANFLLGAASNGAPPAPAAPRPPALWHQGPRR

LPNLVVPGTGPAVHIPGLTAPTLRLHPRPGPQAAALLPQLHEIMPSVAGREAGHPGVPAP

VSGPPLELHHHRRQPGHLGPVLDKATRESAFVHAIASAGVAFAVTRSCAEGTSTICGCDS

HHKGPPGEGWKWGGCSEDADFGVLVSREFADARENRPDARSAMNKHNNEAGRTTILDHMH

LKCKCHGLSGSCEVKTCWWAQPDFRAIGDFLKDKYDSASEMVVEKHRESRGWVETLRAKY

ALFKPPTERDLVYYENSPNFCEPNPETGSFGTRDRTCNVTSHGIDGCDLLCCGRGHNTRT

EKRKEKCHCIFHWCCYVSCQECIRIYDVHTCK

>BOSGR-WNT9B

MRPPPALALAALCLLALPAAAAAAYFGLTGREVLTPFPGLGTAAPAQGGAHLKQCDLLKL

SRRQKQLCRREPGLAETLQDAAHLSLLECQFQFRHERWNCSLEGRTGLLKRGFKETAFLY

AVSAAALTHTLARACSAGRMERCTCDDSPGLESRQAWQWGVCGDNLKYSTKFLNNFLGPK

RGSKDLRARADAHNTHVGIKAVKSGLRTTCKCHGVSGSCAVRTCWKQLSPFRDTGQVLKL

RYDSAVKVSSASNEALGRLELWAPARPGSPSKGPAPRPGDLVYMEDSPSFCRPSKYSPGT

GGRVCSREASCSSLCCGRGYDTQSRLTAFSCHCQVQWCCYVECQQCVREELVYTCKH

>BOSGR-WNT5A

MKKSIGILSPGVAWGTAGRAMSSKFFLMALAIFLSFAQVVIEANSWWSLGMNNPVQMSEV

YIIGAQPLCSQLAGLSQGQKKLCHLYQDHMQYIGEGAKTGIKECQYQFRHRRWNCSTVDN

TSVFGRVMQIGSRETAFTYAVSAAGVVNAMSRACREGELSTCGCSRAARPKDLPRDWLWG

GCGDNIDYGYRFAKEFVDARERERIHAKGSYESARILMNLHNNEAGRRTVYSLADVACKC

HGVSGSCSLKTCWLQLADFRKVGDALKEKYDSAAAMRLNSRGKLVQVNSRFNSPTTQDLV

YIDPSPDYCVRNESTGSLGTQGRLCNKTSEGMDGCELMCCGRGYDQFKTVQTERCHCKFH

WCCYVKCKKCTEIVDQFVCK

>BOSGR-WNT8B

RMLVKPSVCIFLFTCVFQLSHTWSVNNFLMTGPKAYLIYSSSVAAGAQSGIEECKYQFAW

DRWNCPERALQLSSHGGLRSANRETAFVHAISSAGVMYTLTRNCSLGDFDNCGCDDSRNG

QLGGQGWLWGGCSDNVGFGEAISKQFVDALETGQDARAAMNLHNNEAGRKAVKGTMKRTC

KCHGVSGSCTTQTCWLQLPEFREVGAHLKEKYHAALKVDLLQGAGNSAAGRGAIADTFRS

ISTRELVHLEDSPDYCLENKTLGLLGTEGRECLRRGRALGRWERRSCRRLCGDCGLAVEE

RRAETVSSCNCKFHWCCAVRCEQCRRRVTKYFCSRADRPRGGAAHEPGRKP

>BOSGR-WNT16

MDRAALLGLSRLCALWAAVLALFPCGAQGNWMWLGIASFGVPEKLGCANLPLNSRQKELC

KRKPYLLPSIREGARLGIQECRSQFRHERWNCLVAAASAPGTSPLFGYELSSGTKETAFI

YAVMAAGLVHSVTRSCSAGNMTECSCDTTLQNGGSASEGWHWGGCSDDVQYGMWFSRKFL

DFPIKNTTAKESKVLLAMNLHNNEAGRQAVAKLMSLDCRCHGVSGSCAVKTCWKTMSSFE

KIGHLLKDKYENSVQISDKIKRKMHRREKDQRKIPIRKDDLLYVNKSPNYCVEDKKLGIP

GTQGRECNRTSEGADGCNLLCCGRGYNTHVVRHVERCECKFIWCCYVRCRRCESMTDVHT

CK

>BOSMU-WNT3

MEPHLLRLLLGLLLCGTRVLAGYPIWWSLALGQQYTSLGSQPLLCGSIPGLVPKQLRFCR

NYIEIMPSVAEGVKLGIQECQHQFRGRRWNCTTIDDSLAIFGPVLDKATRESAFVHAIAS

AGVAFAVTRSCAEGTSTICGCDSHHKGPPGEGWKWGGCSEDADFGVLVSREFADARENRP

DARSAMNKHNNEAGRTTILDHMHLKCKCHGLSGSCEVKTCWWAQPDFRAIGDFLKDKYDS

ASEMVVEKHRESRGWVETLRAKYALFKPPTERDLVYYENSPNFCEPNPETGSFGTRDRTC

NVTSHGIDGCDLLCCGRGHNTRTEKRKEKCHCIFHWCCYVSCQECIRIYDVHTCK

>BOSMU-WNT9B

FSFPFLLLLLSLVTGLPSLAPFSLTGREVLTPFPGLGTAAPAQGGAHLKQCDLLKLSRRQ

KQLCRREPGLAETLQDAAHLSLLECQFQFRHERWNCSLEGRTGLRLYAVSAAALTHTLAR

ACSAGRMERCTCDDSPGLESRQAWQWGVCGDNLKYSTKFLNNFLGPKRGSKDLRARADAH

NTHVGIKAVKSGLRTTCKCHGVSGSCAVRTCWKQLSPFRDTGQVLKLRYDSAVKVSSASN

EALGRLELWAPARPGSPSKGPAPRPGDLVYMEDSPSFCRPSKYSPGTGGRVCSREASCSS

LCCGRLTAFSCHCQVQWCCYVECQQCVREELVYTCKH

>BOSMU-WNT11

MRARPQVCQALLFALALQTGVCYGIKWLALSKTPAALALNQTQHCKQLEGLVSAQVQLCR

SNLELMHTIVHAAREVMKACRRAFADMRWNCSSIELAPNYLLDLERGTRESAFVYALSAA

AISHAIARACTSGDLPGCSCGPVPGEPPGPGNRWGGCADNLSYGLLMGAKFSDAPMKVKK

TGSQANKLMRLHNSEVGRQALRASLEMKCKCHGVSGSCSIRTCWKGLQELRDVAADLKTR

YLSATKVVHRPMGTRKHLVPKDLDIRPVKDSELIYLQSSPDFCMKNEKVGSHGTQDRQCN

KTSHGSDSCDLMCCGRGYNPYTDRVVERCHCKYHWCCYVTCRRCERTVERYVCK

>BOSMU-WNT2B

MLRPGGAEEAAQLPPRRVSAPVPESAPRPTAPDGSRASARLSLACLLLLLLLLTLPARVD

TSWWYIGALGARVICDNIPGLVSRQRQLCQRYPDIMRSVGEGAREWIRECQHQFRHHRWN

CTTLDRDHTVFGRVMLRSSREAAFVYAISSAGVVHAITRACSQGELSVCSCDPYTRGRHH

DQRGDFDWGGCSDNIHYGVRFAKAFVDAKEKRLKDARALMNLHNNRCGRTAVRRFLKLEC

KCHGVSGSCTLRTCWRALSDFRRTGDYLRRRYDGAVQVTATQDGANFTAARQGYRRATRT

DLVYFDNSPDYCVLDKAAGSLGTAGRVCSKTSKGTDGCEIMCCGRGYDTTRVTRVTQCEC

KFHWCCAVRCKECRNTVDVHTCKAPKKAEWLDQT

>BOSMU-WNT8B

RMLVKPSVCIFLFTCVFQLSHTWSVNNFLMTGPKAYLIYSSSVAAGAQSGIEECKYQFAW

DRWNCPERALQLSSHGGLRSANRETAFVHAISSAGVMYTLTRNCSLGDFDNCGCDDSRNG

QLGGQGWLWGGCSDNVGFGEAISKQFVDALETGQDARAAMNLHNNEAGRKAVKGTMKRTC

KCHGVSGSCTTQTCWLQLPEFREVGAHLKEKYHAALKVDLLQGAGNSAAGRGAIADTFRS

ISTRELVHLEDSPDYCLENKTLGLLGTEGRECLRRGRALGRWERRSCRRLCGDCGLAVEE

RRAETVSSCNCKFHWCCAVRCEQCRRRVTKYFCSRADRPRGGAAHEPGRKP

>BOSMU-WNT5B

LPSLPALLFACWAPLRATASSWWSLAMSPVQRPEMFIIGAQPVCSQLPGLSAGQRKLCQL

YQEHMAYIGEGARTGIRECQHQFRQRRWNCSTVDDASVFGRVLQIGSRETAFTYAVSAAG

VXXXXXXXXXXXXXXXXXXXXXXXXXXXXXXXXXXXXXXXGYRFAKEFVDAREREKNFAK

GSEEQGRVLMNLQNNEAGRRAVYKTADVACKCHGVSGSCSLKTCWLQLAEFRKVGDQLKE

KYDSAAAMRITRRGKLELVNSRFKPPTPEDLVYVDPSPDYCLRDESTGSLGTRGRLCNKT

SEGLDGCALMCCGRGYDQFKSVRTERCHCKFHWCCFVRCKKCTQVVDQFVCK

>BOSMU-WNT9A

XXXXXXXXXXXXXXXXXXXXXXXXXXXXXXXXLTGSEPLTILPLTLEPEAVAQAHYKACD

RLKLERKQRRMCRRDPGVAETLVEAVSMSALECQYQFRFERWNCTLEGRYRASLLKRGFK

ETAFLYAISSAGLTHALAKACSAGRMERCTCDEAPDLENREAWQWGGCGDNLKYSSKFVK

EFLGRRSSKDLRARVDFHNNLVGVKVIKAGVETTCKCHGVSGSCTVRTCWRQLAPFHEVG

KRLKHKYETALKVGSTTNEXXXXXXXXXXXXXXXXXXXXXXXXXXXXXXXXXXXXXXXXX

XXXXXXXXXXXXXXXXXXXXXXXXXXXXXXXXXXVTRPCQCQVRWCCYVECRQCTQREEV

YTCKG

>BOSMU-WNT3A

MAPLGYFIFLYGLKQALGNYPIWWSLAVGPQYSSLGTQPILCASIPGLVPKQLRFCRNYV

EIMPSVAEGIKISIQECQHQFRGRRWNCTTINNSLAIFGPVLDKATRESAFVHAIASAGV

AFAVTRSCAEGSAAICGCSSPGEGWKWGGCSEDIEFGGMVSREFADARENRPDARSAMNR

HNNEAGRQAIASHMHLKCKCHGLSGSCEVKTCWWSQPDFRAIGDFLKDKYDSASEMVVEK

HRESRGWVETLRPRYTYFKVPTERDLVYYEASPNFCEPNPETGSFGTRDRTCNVSSHGID

GCDLLCCGRGHNARTEQRREKCHCVFHWCCYVSCQECARVYDVHTCKR

>BOSMU-WNT5A

LQKSIGILSPGVAWGTAGRAMSSKFFLMALAIFLSFAQVVIEANSWWSLGMNNPVQMSEV

YIIGAQPLCSQLAGLSQGQKKLCHLYQDHMQYIGEGAKTGIKECQYQFRHRRWNCSTVDN

TSVFGRVMQIERSAASAYRGELSTCGCSRAARPKDLPRDWLWGGCGDNVDYGYRFAKEFV

DARERERIHAKGSYESARILMNLHNNEAGRRTVYSLADVACKCHGVSGSCSLKTCWLQLA

DFRKVGDALKEKYDSAAAMRLNSRGKLVQVNSRFNSPTTQDLVYIDPSPDYCVRNESTGS

LGTQGRLCNKTSEGMDGCELMCCGRGYDQFKTVQTERCHCKFHWCCYVKCKKCTEIVDQF

VCK

>BOSMU-WNT2

MNACLVGIWLWLPLLFTWLSPEVSSSWWYMRATSGSSRVMCDNVPGLVSHQRQLCHRHPD

VMRAIGLGVTEWTMECQHQFRQHRWNCNTLDRDHSLFGRVLLRSSRESAFVYAISSAGVV

FAITRACSQGELKSCSCDPKKKGTAKDNKGTFDWGGCSDNIDYGIKFARAFVDAKERKGK

DARALMNLHNNRAGRKAVKRFLKQECKCHGVSGSCTLRTCWLAMADFRKTGNYLWRKYNG

AIQVVMNQDGTGFTVANKRFKKPTKNDLVYFENSPDYCIRDRDAGSLGTAGRVCNLTSRG

MDSCEVMCCGRGYDTSHITRKTKCECKFHWCCAVRCQDCVEALDVHTCKAPKSPEWAAPT

>BOSMU-WNT4

ESPLSSPSTIRLLLVYLAKLSSVGSISEEETCEKLKGLIQRQVQMCKRNLEVMDSVRRGA

QLAIEECQYQFRNRRWNCSTLDSLPVFGKVVTQGTREAAFVYAISSAGVAFAVTRACSSG

ELEKCGCDRTVHGVSPQGFQWSGCSDNIAYGVAFSQSFVDVRERSKGASSSRALMNLHNN

EAGRKVAWAPIPAGKGSSCGIQKGLKTEEVGHALKEKFDGATEVEPRRVGSSRALVPRNA

QFKPHTDEDLVYLEPSPDFCEQDVRSGVLGTRGRTCNKTSKAIDGCELLCCGRGFHTAQV

ELAERCSCKFHWCCFVKCRQCQRLVELHTCR

>BOSMU-WNT7A

XXRKARRCLGHLFLSLGMVYLRIGGFSSVVALGASIICNKIPGLAPRQRAICQSRPDAII

VIGEGSQMGLDECQFQFRNGRWNCSALGERTVFGKELKVGSREAAFTYAIIAAGVAHAIT

AACTQGNLSDCGCDKEKQGQYHRDEGWKWGGCSADIRYGIGFAKVFVDAREIKQNARTLM

NLHNNEAGRKILEENMKLECKCHGVSGSCTTKTCWTTLPQFRELGYVLKDKYNEAVHVEP

VRASRNKRPAFLKIKKPLSYRKPMDTELVYIEKSPSYCEEDPATGSVGTQXXXXXXXXXX

XXXXXXXXXXXXXXXXXXXXXXXXXXXXXXXXXXXXXXXXXXXXXXXXXXXXXXX

>BOSMU-WNT10B

MREEPRPRPPPLGLAGLLFLALCSRCRAAAAPPLPANTVCLTLSGLSKQQLGLCLRSPDV

TASALQGLHIAVHECVHWRCSPQEVTVGPGFRESAFSFSMLAAGVMHAVATACSLGKLVS

CGCGWKGSGEQDRLRAKLLQLQALSRGKSFSHSLPSSGPGSGPSPGPQDTWEWGGCNHDM

DFGEKFSRDFLDSREAPRDIQARMRIHNNRVGRQVVTENLKRKCKCHGTSGSCQLKTCWR

APPEFRAVGAALRERLDRAIFIDTHNRNSGAFQPRLRPRRLSGELVYFENSPDFCERDPT

VGSPGTQGRACNKTSHQLGSCGSLCCGRGHNVLRQTRVERCNCRFHWCCYVLCDECKVTE

WVNVCK

>BOSMU-WNT1

MGHWALLPCWVSAALLLALAALPAALAANSSGRWWGIVNVASSTNLLTDSKSLQLVLEPS

LQLLSRKQRRLIRQNPGILHSVSGGLQSAVRECKWQFRNRRWNCPTASGPHLFGKIVNRG

CRETAFIFAITSAGVTHSVARSCSEGSIESCTCDYRRRGPGGPDWHWGGCSDNIDFGRLF

GREFVDSGEKGRDLRFLMNLHNNEAGRTTVFSEMRQECKCHGMSGSCTVRTCWMRLPTLR

AVGDVLRDRFDGASRVLYGNRGNNRASRAELLRLEPEDPAHKPPSPHDLVYFEKSPNFCT

YSGRLGTAGTAGRACNSSSPALDGCELLCCGRGHRTRMQRRVTERCNCTFHWCCHVSCRN

CTHTRVLHECL

>BOSMU-WNT16

MDRAALLGLSRLCALWAAVLALFPCGAQGNWMWLGIASFGVPEKLGCANLPLNSRQKELC

KRKPYLLPSIREGARLGIQECRSQFRHERWNCLVAAASAPGTSPLFGYELSSGTKETAFI

YAVMAAGLVHSVTRSCSAGNMTECSCDTTLQNGGSASEGWHWGGCSDDVQYGMWVSRKFL

DFPIKNTTAKESKVLLAMNLHNNEAGRQAVAKLMSLDCRCHGVSGSCAVKTCWKTMSSFE

KIGHLLKDKYENSVQISDKIKRKMHRREKDQRKIPIRKDDLLYVNKSPNYCVEDKKLGIP

GTQGRECNRTSEGADGCNLLCCGRGYNTHVVRHVERCECKFIWCCYVRCRRCESMTDVHT

CK

>BOSMU-WNT8A

MLCNLQCLCLVSPSLPLFTPHQGSFHYLIPIHHCLTFSLFGRSVNNFLITGPKAYLTYTT

SVALGAQSGIEECKFQFAWERWNCPENALQLSTHNRLRSATRETSFIHAISSAGIMYTIT

KNCSMGDFENCGCDESKNGKTGGHGWIWGGCSDNVEFGERISKLFVDSLEKGKDARALMN

LHNNRAGRLAVRATMKRTCKCHGISGSCSIQTCWLQLANFRELGNYLKAKYERALKIEMD

KQQLRAGNSAEGHWIPTEAFLPSAEAELIFLEESPDYCTRNSSLGIYGTEGRECLQNSRN

TSRWEQCSCGRLCTECGLQVEERRTEAISSCNCKFQWCCTVKCEQCRHVVNKYYCTRSPG

SAQSRGKGSA

>BOSMU-WNT6

XXPPAPSRLGLLLLLLLSPAHVGGLWWAVGSPLVMDPTSICRKARRLAGRQAELCQAEPE

VVAELARGARLGVRECQFQFRFRRWNCSSHSKAFGRILQQDIRETAFVFAITAAGASXXX

XXXXXXXXXXXXXXXXXXXXXXXXXPGLPGTPGPPGPAGSPDGSAAWEWGGCGDDVDFGD

EKSRLFMDAQHKRGRGDIRMLVQLHNNEAGRLAVRSHTRTECKCHGLSGSCALRTCWQKL

PPFREVGARLLERFHGASRVMGTNDGKALLPAVRTLKPPGRADLLYAADSPDFCAPNRRT

GSPGTRGRACNSSAPDLSGCDLLCCGRGHRQESVLLEENCLCRFHWCCVVQCHRCRVRKE

LSLCL

>BOSMU-WNT10A

MGSTHPCPWLRLRPRPQPRPALCALLFFLLLLAASVPRSAPNDILGLRLPQEPVLNANTV

CLTLPGLSKRQMEVCVRHPDVAASAIQGIQIAIHECQHQFRDQRWNCSSLETRNKIPYES

PIFSRGFRESAFAYAISAAGVVHAVSNACALGKLRACGCDASRRGDEEAFRRKLHRLQLE

ALQRGKGLSHGVPEHPALPPASPGLQDSWEWGGCSPDVGFGERFSKDFLDSREPHRDIHA

RMRLHNNRVGRQAVMENMRRKCKCHGTSGSCQLKTCWQVTPEFRAVGALLRSXXXXXPGL

PGPRRRASPADLVYFEKSPDFCEREPRLDSAGTVGRLCNKSSAGPDGCGSMCCGRGHNIL

RQTRSERCHCRFHWCCFVVCEECRITEWVSVCK

>BOSTH-WNT9A

MLDGPLLARWLAAAFALTLLLAALRPSAAYFGLTGSEPLTILPLTLEPEAVAQAHYKACD

RLKLERKQRRMCRRDPGVAETLVEAVSMSALECQYQFRFERWNCTLEGRYRASLLKRGFK

ETAFLYAISSAGLTHALAKACSAGRMERCTCDEAPDLENREAWQWGGCGDNLKYSSKFVK

EFLGRRSSKDLRARVDFHNNLVGVKVIKAGVETTCKCHGVSGSCTVRTCWRQLAPFHEVG

KRLKHKYETALKVGSTTNEATGEAGAISPPRGRAAGAGGGDPLPRTPELVHLDDSPSFCV

AGRFSPGTAGRRCHREKNCESICCGRGHNTQSRVVTRPCQCQVRWCCYVECRQCTQREEV

YTCKG

>BOSTH-WNT3A

MAPLGYFIFLYGLKQALGNYPIWWSLAVGPQYSSLGTQPILCASIPGLVPKQLRFCRNYV

EIMPSVAEGIKISIQECQHQFRGRRWNCTTINNSLAIFGPVLDKATRESAFVHAIASAGV

AFAVTRSCAEGSAAICGCSSRHQGSPGEGWKWGGCSEDIEFGGMVSREFADARENRPDAR

SAMNRHNNEAGRQAIASHMHLKCKCHGLSGSCEVKTCWWSQPDFRAIGDFLKDKYDSASE

MVVEKHRESRGWVETLRPRYTYFKVPTERDLVYYEASPNFCEPNPETGSFGTRDRTCNVS

SHGIDGCDLLCCGRGHNARTEQRREKCHCVFHWCCYVSCQECARVYDVHTCKR

>BOSTH-WNT10B

MTTGDQRLGSLVWVATPSLQPWRSQSRPCSICPSGPSGPGFDMREEPRPRPPPSGLAGLL

FLALCSRALGNEIQGLKLPGGGEPPLTANTVCLTLSGLSKQQLGLCLRSPDVTASALQGL

HIAVHECQHQLRDQRWNCSALEGGGRLPHHSAILKRGFRESAFSFSMLAAGVMHAVATAC

SLGKLVSCGCGWKGSGEQDRLRAKLLQLQALSRGKSFSHSLPSSGPGSGPSPGPQDTWEW

GGCNHDMDFGEKFSRDFLDSREAPRDIQARMRIHNNRVGRQVVTENLKRKCKCHGTSGSC

QLKTCWRAPPEFRAVGAALRERLDRAIFIDTHNRNSGAFQPRLRPRRLSGELVYFENSPD

FCERDPTVGSPGTQGRACNKTSHQLGSCGSLCCGRGHNVLRQTRVERCNCRFHWCCYVLC

DECKVTEWVNVCK

>BOSTH-WNT2B

MLRPGGAEEAAQLPPRRVSAPVPESAPRSTAPDGSRASARLSLACLLLLLLLLTLPARVD

TSWWYIGALGARVICDNIPGLVSRQRQLCQRYPDIMRSVGEGAREWIRECQHQFRHHRWN

CTTLDRDHTVFGRVMLRSSREAAFVYAISSAGVVHAITRACSQGELSVCSCDPYTRGRHH

DQRGDFDWGGCSDNIHYGVRFAKAFVDAKEKRLKDARALMNLHNNRCGRTAVRRFLKLEC

KCHGVSGSCTLRTCWRALSDFRRTGDYLRRRYDGAVQVTATQDGANFTAARQGYRRATRT

DLVYFDNSPDYCVLDKAAGSLGTAGRVCSKTSKGTDGCEIMCCGRGYDTTRVTRVTQCEC

KFHWCCAVRCKECRNTVDVHTCKAPKKAEWLDQT

>BOSTH-WNT7A

MNRKARRCLGHLFLSLGMVYLRIGGFSSVVALGASIICNKIPGLAPRQRAICQSRPDAII

VIGEGSQMGLDECQFQFRNGRWNCSALGERTVFGKELKVGSREAAFTYAIIAAGVAHAIT

AACTQGNLSDCGCDKEKQGQYHRDEGWKWGGCSADIRYGIGFAKVFVDAREIKQNARTLM

NLHNNEAGRKILEENMKLECKCHGVSGSCTTKTCWTTLPQFRELGYVLKDKYNEAVHVEP

VRASRNKRPAFLKIKKPLSYRKPMDTELVYIEKSPSYCEEDPATGSVGTQGRACNKTAPQ

ASGCDLMCCGRGYNTHQYARVWQCNCKFHWCCYVKCNTCSERTEVYTCK

>BOSTH-WNT7B

MAAGRLRRLEDVRLSERQSWFRPIDGRRAGRRARRTQRPRPAPPPGPPASARAAAPAPAR

CEALEAGHGRPLRRGPRARIPSAPFRRPALGRVAETPAPAGPRALPAGPARPPRERRPTM

LLLSPRSALLSVYCPQLFLILSSGSYLALSSVVALGANIICNKIPGLAPRQRAICQSRPD

AIIVIGEGAQMGINECQYQFRFGRWNCSALGEKTVFGQELRVGSREAAFTYAITAAGVAH

AVTAACSQGNLSNCGCDREKQGYYNQAEGWKWGGCSADVRYGIDFSRRFVDAREIKKNAR

RLMNLHNNEAGRKVLEERMKLECKCHGVSGSCTTRTCWTTLPKFREVGHLLKEKYNVAVQ

VEVVRASRLRQPTFLRIKQLRSYQKPMETDLVYIEKSPNYCEEDAATGSVGTQGRLCNRT

SPGADGCDTMCCGRGYNTHQYTKVWQCNCKFHWCCFVKCNTCSERTEVFTCK

>BOSTH-WNT8B

RMLVKPSVCIFLFTCVFQLSHTWSVNNFLMTGPKAYLIYSSSVAAGAQSGIEECKYQFAW

DRWNCPERALQLSSHGGLRSANRETAFVHAISSAGVMYTLTRNCSLGDFDNCGCDDSRNG

QLGGQGWLWGGCSDNVGFGEAISKQFVDALETGQDARAAMNLHNNEAGRKAVKGTMKRTC

KCHGVSGSCTTQTCWLQLPEFREVGAHLKEKYHAALKVDLLQGAGNSAAGRGAIADTFRS

ISTRELVHLEDSPDYCLENKTLGLLGTEGRECLRRGRALGRWERRSCRRLCGDCGLAVEE

RRAETVSSCNCKFHWCCAVRCEQCRRRVTKYFCSRADRPRGGAAHEPGRKP

>BOSTH-WNT5B

MPSLPALLALLFACWAPLRATASSWWSLAMSPVQRPEMFIIGAQPVCSQLPGLSAGQRKL

CQLYQEHMAYIGEGARTGIRECQHQFRQRRWNCSTVDDASVFGRVLQIGSRETAFTYAVS

AAGVVNAISRACREGELSTCGCSRAARPKDLPRDWLWGGCGDNVDYGYRFAKEFVDARER

EKNFAKGSEEQGRVLMNLQNNEAGRRAVYKTADVACKCHGVSGSCSLKTCWLQLAEFRKV

GDQLKEKYDSAAAMRITRRGKLELVNSRFKPPTPEDLVYVDPSPDYCLRDESTGSLGTRG

RLCNKTSEGLDGCALMCCGRGYNQFKSVRTERCHCKFHWCCFVRCKKCTQVVDQFVCK

>BOSTH-WNT3

MEPHLLRLLLGLLLCGTRVLAGYPIWWSLALGQQYTSLGSQPLLCGSIPGLVPKQLRFCR

NYIEIMPSVAEGVKLGIQECQHQFRGRRWNCTTIDDSLAIFGPVLDKATRESAFVHAIAS

AGVAFAVTRSCAEGTSTICGCDSHHKGPPGEGWKWGGCSEDADFGVLVSREFADARENRP

DARSAMNKHNNEAGRTTILDHMHLKCKCHGLSGSCEVKTCWWAQPDFRAIGDFLKDKYDS

ASEMVVEKHRESRGWVETLRAKYALFKPPTERDLVYYENSPNFCEPNPETGSFGTRDRTC

NVTSHGIDGCDLLCCGRGHNTRTEKRKEKCHCIFHWCCYVSCQECIRIYDVHTCK

>BOSTH-WNT9B

MRPPPALALAALCLLALPAAAAAAYFGLTGREVLTPFPGLGTAAPAQGGAHLKQCDLLKL

SRRQKQLCRREPGLAETLQDAAHLSLLECQFQFRHERWNCSLEGRTGLLKRGFKETAFLY

AVSAAALTHTLARACSAGRMERCTCDDSPGLESRQAWQWGVCGDNLKYSTKFLNNFLGPK

RGSKDLRARADAHNTHVGIKAVKSGLRTTCKCHGVSGSCAVRTCWKQLSPFRDTGQVLKL

RYDSAVKVSSASNEALGRLELWAPARPGSPSKGPAPRPGDLVYMEDSPSFCRPSKYSPGT

GGRVCSREASCSSLCCGRGYDTQSRLTAFSCHCQVQWCCYVECQQCVREELVYTCKH

>BOSTH-WNT11

MRARPQVCQALLFALALQTGVCYGIKWLALSKTPAALALNQTQHCKQLEGLVSAQVQLCR

SNLELMHTIVHAAREVMKACRRAFADMRWNCSSIELAPNYLLDLERGTRESAFVYALSAA

AISHAIARACTSGDLPGCSCGPVPGEPPGPGNRWGGCADNLSYGLLMGAKFSDAPMKVKK

TGSQANKLMRLHNSEVGRQALRASLEMKCKCHGVSGSCSIRTCWKGLQELRDVAADLKTR

YLSATKVVHRPMGTRKHLVPKDLDIRPVKDSELIYLQSSPDFCMKNEKVGSHGTQDRQCN

KTSHGSDSCDLMCCGRGYNPYTDRVVERCHCKYHWCCYVTCRRCERTVERYVCK

>BOSTH-WNT2

MNACLVGIWLWLPLLFTWLSPEVSSSWWYMRATSGSSRVMCDNVPGLVSHQRQLCHRHPD

VMRAIGLGVTEWTMECQHQFRQHRWNCNTLDRDHSLFGRVLLRSSRESAFVYAISSAGVV

FAITRACSQGELKSCSCDPKKKGTAKDNKGTFDWGGCSDNIDYGIKFARAFVDAKERKGK

DARALMNLHNNRAGRKAVKRFLKQECKCHGVSGSCTLRTCWLAMADFRKTGNYLWRKYNG

AIQVVMNQDGTGFTVANKRFKKPTKNDLVYFENSPDYCIRDRDAGSLGTAGRVCNLTSRG

MDSCEVMCCGRGYDTSHITRKTKCECKFHWCCAVRCQDCVEALDVHTCKAPKSPDWAAPT

>BOSTH-WNT6

MQPPAPSRLGLLLLLLLSPAHVGGLWWAVGSPLVMDPTSICRKARRLAGRQAELCQAEPE

VVAELARGARLGVRECQFQFRFRRWNCSSHSKAFGRILQQDIRETAFVFAITAAGASHAV

TQACSMGELLQCGCQAPRGRAPPRPPGLPGTPGPPGPAGSPDGSAAWEWGGCGDDVDFGD

EKSRLFMDAQHKRGRGDIRMLVQLHNNEAGRLAVRSHTRTECKCHGLSGSCALRTCWQKL

PPFREVGARLLERFHGASRVMGTNDGKALLPAVRTLKPPGRADLLYAADSPDFCAPNRRT

GSPGTRGRACNSSAPDLSGCDLLCCGRGHRQESVLLEENCLCRFHWCCVVQCHRCRVRKE

LSLCL

>BOSTH-WNT10A

MGSTHPCPWLRLRPRPQPRPALCALLFFLLLLAASVPRSAPNDILGLRLPQEPVLNANTV

CLTLPGLSKRQMEVCVRHPDVAASAIQGIQIAIHECQHQFRDQRWNCSSLETRNKIPYES

PIFSRGFRESAFAYAISAAGVVHAVSNACALGKLRACGCDASRRGDEEAFRRKLHRLQLE

ALQRGKGLSHGVPEHPALPPASPGLQDSWEWGGCSPDVGFGERFSKDFLDSREPHRDIHA

RMRLHNNRVGRQAVMENMRRKCKCHGTSGSCQLKTCWQVTPEFRAVGALLRSRFHRATLI

RPHNRNSGQLEPGPAGAPSPAPGLPGPRRRASPADLVYFEKSPDFCEREPRLDSAGTVGR

LCNKSSAGPDGCGSMCCGRGHNILRQTRSERCHCRFHWCCFVVCEECRITEWVSVCK

>BOSTH-WNT16

MDRAALLGLSRLCALWAAVLALFPCGAQGNWMWLGIASFGVPEKLGCANLPLNSRQKELC

KRKPYLLPSIREGARLGIQECRSQFRHERWNCLVAAASAPGTSPLFGYELSSGTKETAFI

YAVMAAGLVHSVTRSCSAGNMTECSCDTTLQNGGSASEGWHWGGCSDDVQYGMWFSRKFL

DFPIKNTTAKESKVLLAMNLHNNEAGRQAVAKLMSLDCRCHGVSGSCAVKTCWKTMSSFE

KIGHLLKDKYENSVQISDKIKRKMHRREKDQRKIPIRKDDLLYVNKSPNYCVEDKKLGIP

GTQGRECNRTSEGADGCNLLCCGRGYNTHVVRHVERCECKFIWCCYVRCRRCESMTDVHT

CK

>BOSTH-WNT1

MGHWALLPCWVSAALLLALAALPAALAANSSGRWWGIVNVASSTNLLTDSKSLQLVLEPS

LQLLSRKQRRLIRQNPGILHSVSGGLQSAVRECKWQFRNRRWNCPTASGPHLFGKIVNRG

CRETAFIFAITSAGVTHSVARSCSEGSIESCTCDYRRRGPGGPDWHWGGCSDNIDFGRLF

GREFVDSGEKGRDLRFLMNLHNNEAGRTTVFSEMRQECKCHGMSGSCTVRTCWMRLPTLR

AVGDVLRDRFDGASRVLYGNRGNNRASRAELLRLEPEDPAHKPPSPHDLVYFEKSPNFCT

YSGRLGTAGTAGRACNSSSPALDGCELLCCGRGHRTRTQRVTERCNCTFHWCCHVSCRNC

THTRVLHECL

>BOSTH-WNT4

MGAMKPPLTCTWSQSALTESCVIWPGIPFQPCEVGQVLMPHPRAVKNPEAEQGAWHTLFS

KLTRPHCRPQPPVLTAHIVLSATAVILVSAWGPGKWLAMRIAAFVSQKVGLGHGGPCLGM

REGDTADQTAAPRLPDRGHLDMQGLEWTRSGVTSGLRCRWPRGPPGGEGSHGGSVSGSPR

ASFNSSRDRGLVQDSPSPLLPSTAQAGGLSLDPERSQGLIAGTPGSQSPGHGGGSPPGRG

RRRGGALPGIPPPPNPALPRYLAKLSSVGSISEEETCEKLKGLIQRQVQMCKRNLEVMDS

VRRGAQLAIEECQYQFRNRRWNCSTLDSLPVFGKVVTQGTREAAFVYAISSAGVAFAVTR

ACSSGELEKCGCDRTVHGVSPQGFQWSGCSDNIAYGVAFSQSFVDVRERSKGASSSRALM

NLHNNEAGRKAILTHMRVECKCHGVSGSCEVKTCWRAVPPFRQVGHALKEKFDGATEVEP

RRVGSSRALVPRNAQFKPHTDEDLVYLEPSPDFCEQDVRSGVLGTRGRTCNKTSKAIDGC

ELLCCGRGFHTAQVELAERCSCKFHWCCFVKCRQCQRLVELHTCR

>BOSTH-WNT8A

MLCNLQCLCLVSPSLPLFTPHQGSFHYLIPIHHCLTFSLFGRSVNNFLITGPKAYLTYTT

SVALGAQSGIEECKFQFAWERWNCPENALQLSTHNRLRSATRETSFIHAISSAGVMYTIT

KNCSMGDFENCGCDESKNGKTGGHGWIWGGCSDNVEFGERISKLFVDSLEKGKDARALMN

LHNNRAGRLAVRATMKRTCKCHGISGSCSIQTCWLQLANFRELGNYLKAKYERALKIEMD

KQQLRAGNSAEGHWIPTEAFLPSAEAELIFLEESPDYCTRNSSLGIYGTEGRECLQNSRN

TSRWEQCSCGRLCTECGLQVEERRTEAISSCNCKFQWCCTVKCEQCRHVVNKYYCTSSPG

SAQPRGKGSA

>BOSTH-WNT5A

MKKSIGILSPGVAWGTAGRAMSSKFFLMALAIFLSFAQVVIEANSWWSLGMNNPVQMSEV

YIIGAQPLCSQLAGLSQGQKKLCHLYQDHMQYIGEGAKTGIKECQYQFRHRRWNCSTVDN

TSVFGRVMQIGSRETAFTYAVSAAGVVNAMSRACREGELSTCGCSRAARPKDLPRDWLWG

GCGDNIDYGYRFAKEFVDARERERIHAKGSYESARILMNLHNNEAGRRTVYSLADVACKC

HGVSGSCSLKTCWLQLADFRKVGDALKEKYDSAAAMRLNSRGKLVQVNSRFNSPTTQDLV

YIDPSPDYCVRNESTGSLGTQGRLCNKTSEGMDGCELMCCGRGYDQFKTVQTERCHCKFH

WCCYVKCKKCTEIVDQFVCK

>BUBBU-WNT3

MEPHLLRLLLGLLLCGTRVLAGYPIWWSLALGQQYTSLGSQPLLCGSIPGLVPKQLRFCRNYIEIMPSVAEGVKLGIQEC

QHQFRGRRWNCTTIDDSLAIFGPVLDKATRESAFVHAIASAGVAFAVTRSCAEGTSTICGCDSHHKGPPGEGWKWGGCSE

DADFGVLVSREFADARENRPDARSAMNKHNNEAGRTTILDHMHLKCKCHGLSGSCEVKTCWWAQPDFRAIGDFLKDKYDS

ASEMVVEKHRESRGWVETLRAKYALFKPPTERDLVYYENSPNFCEPNPETGSFGTRDRTCNVTSHGIDGCDLLCCGRGHN

TRTEKRKEKCHCIFHWCCYVSCQECIRIYDVHTCK

>BUBBU-WNT16

MDRAALLGLSRLCALWAAVLALFPCGAQGNWMWLGIASFGVPEKLGCANLPLNSRQKELCKRKPYLLPSIREGARLGIQE

CRSQFRHERWNCLVAASSPPGTSPLFGYELSSGTKETAFIYAVMAAGLVHSVTRSCSAGNMTECSCDTTLQNGGSASEGW

HWGGCSDDVQYGMWFSRKFLDFPIRNTTAKESKVLLAMNLHNNEAGRQAVAKLMSLDCRCHGVSGSCAVKTCWKTMSSFE

KIGHLLKDKYENSVQISDKIKRKMHRREKDQRKIPIRKDDLLYVNKSPNYCVEDKKLGIPGTQGRECNRTSEGADGCNLL

CCGRGYNTHVVRHVERCECKFIWCCYVRCRRCESMTDVHTCK

>BUBBU-WNT8A

MGDLLMLRVAVGICYVTFSASAWSVNNFLITGPKAYLTYTTSVALGAQSGIEECKFQFAWERWNCPENALQLSTHNRLRS

ATRETSFIHAISSAGVMYTITKNCSMGDFENCGCDESKNGKTGGHGWIWGGCSDNVEFGERISKLFVDSLEKGKDARALM

NLHNNRAGRLAVRATMKRTCKCHGISGSCSIQTCWLQLANFRELGNYLKAKYEQALKIEMDKQQLRAGNSAEGHWIPTEA

FLPSAEAELIFLEDSPDYCTRNSSLGIYGTEGRECLQNSRNTSRWEQCSCGRLCTECGLQVEERRTEAISSCHCKFQWCC

AVKCDQCRHVVSKYYCTRSPRSARSRGKGSA

>BUBBU-WNT5A

MKKSIGILSPGVAWGTAGRAMSSKFFLMALAIFLSFAQVVIEANSWWSLGMNNPVQMSEVYIIGAQPLCSQLAGLSQGQK

KLCHLYQDHMQYIGEGAKTGIKECQYQFRHRRWNCSTVDNTSVFGRVMQIGSRETAFTYAVSAAGVVNAMSRACREGELS

TCGCSRAARPKDLPRDWLWGGCGDNIDYGYRFAKEFVDARERERIHAKGSYESARILMNLHNNEAGRRTVYSLADVACKC

HGVSGSCSLKTCWLQLADFRKVGDALKEKYDSAAAMRLNSRGKLVQVNSRFNSPTTQDLVYIDPSPDYCVRNESTGSLGT

QGRLCNKTSEGMDGCELMCCGRGYDQFKTVQTERCHCKFHWCCYVKCKKCTEIVDQFVCK

>BUBBU-WNT1

MGHWALLPCWVSAALLLALAALPAALAANSSGRWWGIVNVASSTNLLTDSKSLQLVLEPSLQLLSRKQRRLIRQNPGILH

SVSGGLQSAVRECKWQFRNRRWNCPTASGPHLFGKIVNRGCRETAFIFAITSAGVTHSVARSCSEGSIESCTCDYRRRGP

GGPDWHWGGCSDNIDFGRLFGREFVDSGEKGRDLRFLMNLHNNEAGRTTVFSEMRQECKCHGMSGSCTVRTCWMRLPTLR

AVGDVLRDRFDGASRVLYGNRGNNRASRAELLRLEPEDPAHKPPSPHDLVYFEKSPNFCTYSGRLGTAGTAGRACNSSSP

ALDGCELLCCGRGHRTRTQRVTERCNCTFHWCCHVSCRNCTHTRVLHECL

>BUBBU-WNT10A

MGSTHPCPWLRLRPRPQPRPALCALLFFLLLLAASVPRSAPNDILGLRLPQEPVLNANTVCLTLPGLSKRQMEVCVRHPD

VAASAIQGIQIAIHECQHQFRDQRWNCSSLETRNKIPYESPIFSRGFRESAFAYAISAAGVVHAVSNACALGKLRACGCD

ASRRGDEEAFRRKLHRLQLEALQRGKGLSHGVPEHPALPPASPGLQDSWEWGGCSPDVGFGERFSKDFLDSREPHRDIHA

RMRLHNNRVGRQAVMENMRRKCKCHGTSGSCQLKTCWQVTPEFRAVGALLRSRFHRATLIRPHNRNSGQLEPGPAGAPSP

APGLPGPRRRASPADLVYFEKSPDFCEREPRLDSAGTVGRLCNKSSAGPDGCGSMCCGRGHNILRQTRSERCHCRFHWCC

FVVCEECRITEWVSVCK

>BUBBU-WNT6

MMQPPAPSRLGLLLLLLLCPAHVGGLWWAVGSPLVMDPTSICRKARRLAGRQAELCQAEPEVVAELARGARLGVRECQFQ

FRFRRWNCSSHSKAFGRILQQDIRETAFVFAITAAGASHAVTQACSMGELLQCGCQAPRGRAPPRPPGLPGTPGPPGPAG

SPDGSAAWEWGGCGDDVDFGDEKSRLFMDAQHKRGRGDIRMLVQLHNNEAGRLAVRSHTRTECKCHGLSGSCALRTCWQK

LPPFREVGARLLERFHGASRVMGTNDGKALLPAVRTLKPPGRADLLYAADSPDFCAPNRRTGSPGTRGRACNSSAPDLSG

CDLLCCGRGHRQESVLLEENCLCRFHWCCVVQCHRCRVRKELSLCL

>BUBBU-WNT9A

MSDAEASGCRDDAWWAGVLWARPPALLVLCGQPVPGLAALALQGGPPGLLFCCHCAQQPSGSAAAPASAAAHPVPASHLW

SRAHFGLCVPRLTGSEPLTILPLTLEPEAVAQAHYKACDRLKLERKQRRMCRRDPGVAETLVEAVSMSALECQYQFRFER

WNCTLEGRYRASLLKRGFKETAFLYAISSAGLTHALAKACSAGRMERCTCDEAPDLENREAWQWGGCGDNLKYSSKFVKE

FLGRRSSKDLRARVDFHNNLVGVKVIKAGVETTCKCHGVSGSCTVRTCWRQLAPFHEVGKRLKHKYETALKVGSTTNEAT

GEAGAISPPRGRAAGAGGGDPLPRTPELVHLDDSPSFCVAGRFSPGTAGRRCHREKNCESICCGRGHNTQSRVVTRPCQC

QVRWCCYVECRQCTQREEVYTCKG

>BUBBU-WNT2

MNACLVGIWLWLPLLFTWLSPEVSSSWWYMRATSGSSRVMCDNVPGLVSHQRQLCHRHPDVMRAIGLGVTEWTMECQHQF

RQHRWNCNTLDRDHSLFGRVLLRSSRESAFVYAISSAGVVFAITRACSQGELKSCSCDPKKKGTAKDNKGTFDWGGCSDN

IDYGIKFARAFVDAKERKGKDARALMNLHNNRAGRKAVKRFLKQECKCHGVSGSCTLRTCWLAMADFRKTGNYLWRKYNG

AIQVVMNQDGTGFTVANKRFKKPTKNDLVYFENSPDYCIRDRDAGSLGTAGRVCNLTSRGMDSCEVMCCGRGYDTSHITR

KTKCECKFHWCCAVRCQDCVEALDVHTCKAPKSPDWAAPT

>BUBBU-WNT2B

MLRPGGAEEAAQLPTRRVRAPVPESAPRPTAPDGSRASARLSLACLLLLLLLLTLPARVDTSWWYIGALGARVICDNIPG

LVSRQRQLCQRYPDIMRSVGEGAREWIRECQHQFRHHRWNCTTLDRDHTVFGRVMLRSSREAAFVYAISSAGVVHAITRA

CSQGELSVCSCDPYTRGRHHDQRGDFDWGGCSDNIHYGVRFAKAFVDAKEKRLKDARALMNLHNNRCGRTAVRRFLKLEC

KCHGVSGSCTLRTCWRALSDFRRTGDYLRRRYDGAVQVTATQDGANFTAARQGYRRATRTDLVYFDNSPDYCVLDKAAGS

LGTAGRVCSKTSKGTDGCEIMCCGRGYDTTRVTRVTQCECKFHWCCAVRCKECRNTVDVHTCKAPKKAEWLDQT

>BUBBU-WNT7A

MNRKARRCLGHLFLSLGMVYLRIGGFSSVVALGASIICNKIPGLAPRQRAICQSRPDAIIVIGEGSQMGLDECQFQFRNG

RWNCSALGERTVFGKELKVGSREAAFTYAIIAAGVAHAITAACTQGNLSDCGCDKEKQGQYHRDEGWKWGGCSADIRYGI

GFAKVFVDAREIKQNARTLMNLHNNEAGRKILEENMKLECKCHGVSGSCTTKTCWTTLPQFRELGYVLKDKYNEAVHVEP

VRASRNKRPAFLKIKKPLSYRKPMDTELVYIEKSPSYCEEDPATGSVGTQGRACNKTAPQASGCDLMCCGRGYNTHQYAR

VWQCNCKFHWCCYVKCNTCSERTEVYTCK

>BUBBU-WNT11

MRARPQVCQALLFALALQTGVCYGIKWLALSKTPAALALNQTQHCKQLEGLVSAQVQLCRSNLELMHTIVHAAREVMKAC

RRAFADMRWNCSSIELAPNYLLDLERGTRESAFVYALSAAAISHAIARACTSGDLPGCSCGPVPGEPPGPGNRWGGCADN

LSYGLLMGAKFSDAPMKVKKTGSQANKLMRLHNSEVGRQALRASLEMKCKCHGVSGSCSIRTCWKGLQELRDVAADLKTR

YLSATKVVHRPMGTRKHLVPKDLDIRPVKDSELIYLQSSPDFCMKNEKVGSHGTQDRQCNKTSHGSDSCDLMCCGRGYNP

YTDRVVERCHCKYHWCCYVTCRRCERTVERYVCK

>BUBBU-WNT8B

MSLRSVNNFLMTGPKAYLIYSSSVAAGAQSGIEECKYQFAWDRWNCPERALQLSSHGGLRSANRETAFVHAISSAGVMYT

LTRNCSLGDFDNCGCDDSRNGQLGGQGWLWGGCSDNVGFGEAISKQFVDALETGQDARAAMNLHNNEAGRKAVKGTMKRT

CKCHGVSGSCTTQTCWLQLPEFREVGAHLKEKYHAALKVDLLQGAGNSAAGRGAIADTFRSISTRELVHLEDSPDYCLEN

KTLGLLGTEGRECLRRGRALGRWERRSCRRLCGDCGLAVEERRAETVSSCNCKFHWCCAVRCEQCRRRVTKYFCSRADRP

PGGAAHEPGRKP

>BUBBU-WNT4

MSPRWCLRSLRLLVFAVFSAAASNWLYLAKLSSVGSISEEETCEKLKGLIQRQVQMCKRNLEVMDSVRRGAQLAIEECQY

QFRNRRWNCSTLDSLPVFGKVVTQGTREAAFVYAISSAGVAFAVTRACSSGELEKCGCDRTVHGVSPQGFQWSGCSDNIA

YGVAFSQSFVDVRERSKGASSSRALMNLHNNEAGRKAILTHMRVECKCHGVSGSCEVKTCWRAVPPFRQVGHALKEKFDG

ATEVEPRRVGSSRALVPRNAQFKPHTDEDLVYLEPSPDFCEQDVRSGVLGTRGRTCNKTSKAIDGCELLCCGRGFHTAQV

ELAERCSCKFHWCCFVKCRQCQRLVELHTCR

>BUBBU-WNT9B

MRPPPALALAALCLLALPAAAAAAYFGLTGREVLTPFPGLGTAAPAQGGAHLKQCDLLKLSRRQKQLCRREPGLAETLQD

AAHLSLLECQFQFRHERWNCSLEGRTGLLKRGFKETAFLYAVSAAALTHTLARACSAGRMERCTCDDSPGLESRQAWQWG

VCGDNLKYSTKFLNNFLGPKRGSKDLRARADAHNTHVGIKAVKSGLRTTCKCHGVSGSCAVRTCWKQLSPFRDTGQVLKL

RYDSAVKVSSASNEALGRLELWAPARPGSPSKGPAPRPGDLVYMEDSPSFCRPSKYSPGTGGRVCSREASCSSLCCGRGY

DTQSRLAAFSCHCQVQWCCYVECQQCVREELVYTCKH

>BUBBU-WNT7B

MLLLSPRSALLSVYCPQLFLILSSGSYLALSSVVALGANIICNKIPGLAPRQRAICQSRPDAIIVIGEGAQMGINECQYQ

FRFGRWNCSALGEKTVFGQELRVGSREAAFTYAITAAGVAHAVTAACSQGNLSNCGCDREKQGYYNQAEGWKWGGCSADV

RYGIDFSRRFVDAREIKKNARRLMNLHNNEAGRKVLEERMKLECKCHGVSGSCTTKTCWTTLPKFREVGHLLKEKYNVAV

QVEVVRASRLRQPTFLRIKQLRSYQKPMETDLVYIEKSPNYCEEDAATGSVGTQGRLCNRTSPGADGCDTMCCGRGYNTH

QYTKVWQCNCKFHWCCFVKCNTCSERTEVFTCK

>BUBBU-WNT5B

MPSLPALLALLFACWAPLRAAASSWWSLAMSPVQRPEMFIIGAQPVCSQLPGLSAGQRKLCQLYQEHMAYIGEGARTGIR

ECQHQFRQRRWNCSTVDDASVFGRVLQIGSRETAFTYAVSAAGVVNAISRACREGELSTCGCSRAARPKDLPRDWLWGGC

GDNVDYGYRFAKEFVDAREREKNFAKGSEEQGRVLMNLQNNEAGRRAVYKTADVACKCHGVSGSCSLKTCWLQLAEFRKV

GDQLKEKYDSAAAMRITRRGKLELVNSRFKPPTPEDLVYVDPSPDYCLRDESTGSLGTRGRLCNKTSEGLDGCALMCCGR

GYDQFKSVRTERCHCKFHWCCFVRCKKCTQVVDQFVCK

>BUBBU-WNT10B

MREEPRPRPPPSGLAGLLFLALCSRALGNEIQGLKLPGGGEPPLTANTVCLTLSGLSKQQLGLCLRSPDVTASALQGLHI

AVHECQHQLRDQRWNCSALEGGGRLPHHSAILKRGFRESAFSFSMLAAGVMHAVATACSLGKLVSCGCGWKGSGEQDRLR

AKLLQLQALSRGKSFPHSLPSSGPGSGPSPGPQDTWEWGGCNHDMDFGEKFSRDFLDSREAPRDIQARMRIHNNRVGRQV

VTENLKRKCKCHGTSGSCQLKTCWRAPPEFRAVGAALRERLDRAIFIDTHNRNSGAFQPRLRPRRLSGELVYFENSPDFC

ERDPTVGSPGTQGRACNKTSPLLGSCGSLCCGRGHNVLRQTRVERCNCRFHWCCYVLCDECKVTEWVNVCK

>BUBBU-WNT3A

MAPLGYFIFLYGLKQALGNYPIWWSLAVGPQYSSLGTQPILCASIPGLVPKQLRFCRNYVEIMPSVAEGIKISIQECQHQ

FRGRRWNCTTINNSLAIFGPVLDKATRESAFVHAIASAGVAFAVTRSCAEGSAAICGCSNRHQGSPGEGWKWGGCSEDIE

FGGMVSREFADARENRPDARSAMNRHNNEAGRQAIASHMHLKCKCHGLSGSCEVKTCWWSQPDFRAIGDFLKDKYDSASE

MVVEKHRESRGWVETLRPRYTYFKVPTERDLVYYEASPNFCEPNPETGSFGTRDRTCNVSSHGIDGCDLLCCGRGHNART

EQRREKCHCVFHWCCYVSCQECARVYDVHTCK

>HUMAN-WNT16

MDRAALLGLARLCALWAALLVLFPYGAQGNWMWLGIASFGVPEKLGCANLPLNSRQKELC

KRKPYLLPSIREGARLGIQECGSQFRHERWNCMITAAATTAPMGASPLFGYELSSGTKET

AFIYAVMAAGLVHSVTRSCSAGNMTECSCDTTLQNGGSASEGWHWGGCSDDVQYGMWFSR

KFLDFPIGNTTGKENKVLLAMNLHNNEAGRQAVAKLMSVDCRCHGVSGSCAVKTCWKTMS

SFEKIGHLLKDKYENSIQISDKTKRKMRRREKDQRKIPIHKDDLLYVNKSPNYCVEDKKL

GIPGTQGRECNRTSEGADGCNLLCCGRGYNTHVVRHVERCECKFIWCCYVRCRRCESMTD

VHTCK

>HUMAN-WNT8B

MFLSKPSVYICLFTCVLQLSHSWSVNNFLMTGPKAYLIYSSSVAAGAQSGIEECKYQFAW

DRWNCPERALQLSSHGGLRSANRETAFVHAISSAGVMYTLTRNCSLGDFDNCGCDDSRNG

QLGGQGWLWGGCSDNVGFGEAISKQFVDALETGQDARAAMNLHNNEAGRKAVKGTMKRTC

KCHGVSGSCTTQTCWLQLPEFREVGAHLKEKYHAALKVDLLQGAGNSAAGRGAIADTFRS

ISTRELVHLEDSPDYCLENKTLGLLGTEGRECLRRGRALGRWERRSCRRLCGDCGLAVEE

RRAETVSSCNCKFHWCCAVRCEQCRRRVTKYFCSRAERPRGGAAHKPGRKP

>HUMAN-WNT11

MRARPQVCEALLFALALQTGVCYGIKWLALSKTPSALALNQTQHCKQLEGLVSAQVQLCR

SNLELMHTVVHAAREVMKACRRAFADMRWNCSSIELAPNYLLDLERGTRESAFVYALSAA

AISHAIARACTSGDLPGCSCGPVPGEPPGPGNRWGGCADNLSYGLLMGAKFSDAPMKVKK

TGSQANKLMRLHNSEVGRQALRASLEMKCKCHGVSGSCSIRTCWKGLQELQDVAADLKTR

YLSATKVVHRPMGTRKHLVPKDLDIRPVKDSELVYLQSSPDFCMKNEKVGSHGTQDRQCN

KTSNGSDSCDLMCCGRGYNPYTDRVVERCHCKYHWCCYVTCRRCERTVERYVCK

>HUMAN-WNT10B

MLEEPRPRPPPSGLAGLLFLALCSRALSNEILGLKLPGEPPLTANTVCLTLSGLSKRQLG

LCLRNPDVTASALQGLHIAVHECQHQLRDQRWNCSALEGGGRLPHHSAILKRGFRESAFS

FSMLAAGVMHAVATACSLGKLVSCGCGWKGSGEQDRLRAKLLQLQALSRGKSFPHSLPSP

GPGSSPSPGPQDTWEWGGCNHDMDFGEKFSRDFLDSREAPRDIQARMRIHNNRVGRQVVT

ENLKRKCKCHGTSGSCQFKTCWRAAPEFRAVGAALRERLGRAIFIDTHNRNSGAFQPRLR

PRRLSGELVYFEKSPDFCERDPTMGSPGTRGRACNKTSRLLDGCGSLCCGRGHNVLRQTR

VERCHCRFHWCCYVLCDECKVTEWVNVCK

>HUMAN-WNT3A

MAPLGYFLLLCSLKQALGSYPIWWSLAVGPQYSSLGSQPILCASIPGLVPKQLRFCRNYV

EIMPSVAEGIKIGIQECQHQFRGRRWNCTTVHDSLAIFGPVLDKATRESAFVHAIASAGV

AFAVTRSCAEGTAAICGCSSRHQGSPGKGWKWGGCSEDIEFGGMVSREFADARENRPDAR

SAMNRHNNEAGRQAIASHMHLKCKCHGLSGSCEVKTCWWSQPDFRAIGDFLKDKYDSASE

MVVEKHRESRGWVETLRPRYTYFKVPTERDLVYYEASPNFCEPNPETGSFGTRDRTCNVS

SHGIDGCDLLCCGRGHNARAERRREKCRCVFHWCCYVSCQECTRVYDVHTCK

>HUMAN-WNT6

MLPPLPSRLGLLLLLLLCPAHVGGLWWAVGSPLVMDPTSICRKARRLAGRQAELCQAEPE

VVAELARGARLGVRECQFQFRFRRWNCSSHSKAFGRILQQDIRETAFVFAITAAGASHAV

TQACSMGELLQCGCQAPRGRAPPRPSGLPGTPGPPGPAGSPEGSAAWEWGGCGDDVDFGD

EKSRLFMDARHKRGRGDIRALVQLHNNEAGRLAVRSHTRTECKCHGLSGSCALRTCWQKL

PPFREVGARLLERFHGASRVMGTNDGKALLPAVRTLKPPGRADLLYAADSPDFCAPNRRT

GSPGTRGRACNSSAPDLSGCDLLCCGRGHRQESVQLEENCLCRFHWCCVVQCHRCRVRKE

LSLCL

>HUMAN-WNT7B

MHRNFRKWIFYVFLCFGVLYVKLGALSSVVALGANIICNKIPGLAPRQRAICQSRPDAII

VIGEGAQMGINECQYQFRFGRWNCSALGEKTVFGQELRVGSREAAFTYAITAAGVAHAVT

AACSQGNLSNCGCDREKQGYYNQAEGWKWGGCSADVRYGIDFSRRFVDAREIKKNARRLM

NLHNNEAGRKVLEDRMQLECKCHGVSGSCTTKTCWTTLPKFREVGHLLKEKYNAAVQVEV

VRASRLRQPTFLRIKQLRSYQKPMETDLVYIEKSPNYCEEDAATGSVGTQGRLCNRTSPG

ADGCDTMCCGRGYNTHQYTKVWQCNCKFHWCCFVKCNTCSERTEVFTCK

>HUMAN-WNT5A

MKKSIGILSPGVALGMAGSAMSSKFFLVALAIFFSFAQVVIEANSWWSLGMNNPVQMSEV

YIIGAQPLCSQLAGLSQGQKKLCHLYQDHMQYIGEGAKTGIKECQYQFRHRRWNCSTVDN

TSVFGRVMQIGSRETAFTYAVSAAGVVNAMSRACREGELSTCGCSRAARPKDLPRDWLWG

GCGDNIDYGYRFAKEFVDARERERIHAKGSYESARILMNLHNNEAGRRTVYNLADVACKC

HGVSGSCSLKTCWLQLADFRKVGDALKEKYDSAAAMRLNSRGKLVQVNSRFNSPTTQDLV

YIDPSPDYCVRNESTGSLGTQGRLCNKTSEGMDGCELMCCGRGYDQFKTVQTERCHCKFH

WCCYVKCKKCTEIVDQFVCK

>HUMAN-WNT7A

MNRKARRCLGHLFLSLGMVYLRIGGFSSVVALGASIICNKIPGLAPRQRAICQSRPDAII

VIGEGSQMGLDECQFQFRNGRWNCSALGERTVFGKELKVGSREAAFTYAIIAAGVAHAIT

AACTQGNLSDCGCDKEKQGQYHRDEGWKWGGCSADIRYGIGFAKVFVDAREIKQNARTLM

NLHNNEAGRKILEENMKLECKCHGVSGSCTTKTCWTTLPQFRELGYVLKDKYNEAVHVEP

VRASRNKRPTFLKIKKPLSYRKPMDTDLVYIEKSPNYCEEDPVTGSVGTQGRACNKTAPQ

ASGCDLMCCGRGYNTHQYARVWQCNCKFHWCCYVKCNTCSERTEMYTCK

>HUMAN-WNT9B

MRPPPALALAGLCLLALPAAAASYFGLTGREVLTPFPGLGTAAAPAQGGAHLKQCDLLKL

SRRQKQLCRREPGLAETLRDAAHLGLLECQFQFRHERWNCSLEGRMGLLKRGFKETAFLY

AVSSAALTHTLARACSAGRMERCTCDDSPGLESRQAWQWGVCGDNLKYSTKFLSNFLGSK

RGNKDLRARADAHNTHVGIKAVKSGLRTTCKCHGVSGSCAVRTCWKQLSPFRETGQVLKL

RYDSAVKVSSATNEALGRLELWAPARQGSLTKGLAPRSGDLVYMEDSPSFCRPSKYSPGT

AGRVCSREASCSSLCCGRGYDTQSRLVAFSCHCQVQWCCYVECQQCVQEELVYTCKH

>HUMAN-WNT4

MSPRSCLRSLRLLVFAVFSAAASNWLYLAKLSSVGSISEEETCEKLKGLIQRQVQMCKRN

LEVMDSVRRGAQLAIEECQYQFRNRRWNCSTLDSLPVFGKVVTQGTREAAFVYAISSAGV

AFAVTRACSSGELEKCGCDRTVHGVSPQGFQWSGCSDNIAYGVAFSQSFVDVRERSKGAS

SSRALMNLHNNEAGRKAILTHMRVECKCHGVSGSCEVKTCWRAVPPFRQVGHALKEKFDG

ATEVEPRRVGSSRALVPRNAQFKPHTDEDLVYLEPSPDFCEQDMRSGVLGTRGRTCNKTS

KAIDGCELLCCGRGFHTAQVELAERCSCKFHWCCFVKCRQCQRLVELHTCR

>HUMAN-WNT8A

MGNLFMLWAALGICCAAFSASAWSVNNFLITGPKAYLTYTTSVALGAQSGIEECKFQFAW

ERWNCPENALQLSTHNRLRSATRETSFIHAISSAGVMYIITKNCSMGDFENCGCDGSNNG

KTGGHGWIWGGCSDNVEFGERISKLFVDSLEKGKDARALMNLHNNRAGRLAVRATMKRTC

KCHGISGSCSIQTCWLQLAEFREMGDYLKAKYDQALKIEMDKRQLRAGNSAEGHWVPAEA

FLPSAEAELIFLEESPDYCTCNSSLGIYGTEGRECLQNSHNTSRWERRSCGRLCTECGLQ

VEERKTEVISSCNCKFQWCCTVKCDQCRHVVSKYYCARSPGSAQSLGKGSA

>HUMAN-WNT1

MGLWALLPGWVSATLLLALAALPAALAANSSGRWWGIVNVASSTNLLTDSKSLQLVLEPS

LQLLSRKQRRLIRQNPGILHSVSGGLQSAVRECKWQFRNRRWNCPTAPGPHLFGKIVNRG

CRETAFIFAITSAGVTHSVARSCSEGSIESCTCDYRRRGPGGPDWHWGGCSDNIDFGRLF

GREFVDSGEKGRDLRFLMNLHNNEAGRTTVFSEMRQECKCHGMSGSCTVRTCWMRLPTLR

AVGDVLRDRFDGASRVLYGNRGSNRASRAELLRLEPEDPAHKPPSPHDLVYFEKSPNFCT

YSGRLGTAGTAGRACNSSSPALDGCELLCCGRGHRTRTQRVTERCNCTFHWCCHVSCRNC

THTRVLHECL

>HUMAN-WNT2B

MLRPGGAEEAAQLPLRRASAPVPVPSPAAPDGSRASARLGLACLLLLLLLTLPARVDTSW

WYIGALGARVICDNIPGLVSRQRQLCQRYPDIMRSVGEGAREWIRECQHQFRHHRWNCTT

LDRDHTVFGRVMLRSSREAAFVYAISSAGVVHAITRACSQGELSVCSCDPYTRGRHHDQR

GDFDWGGCSDNIHYGVRFAKAFVDAKEKRLKDARALMNLHNNRCGRTAVRRFLKLECKCH

GVSGSCTLRTCWRALSDFRRTGDYLRRRYDGAVQVMATQDGANFTAARQGYRRATRTDLV

YFDNSPDYCVLDKAAGSLGTAGRVCSKTSKGTDGCEIMCCGRGYDTTRVTRVTQCECKFH

WCCAVRCKECRNTVDVHTCKAPKKAEWLDQT

>HUMAN-WNT5B

MPSLLLLFTAALLSSWAQLLTDANSWWSLALNPVQRPEMFIIGAQPVCSQLPGLSPGQRK

LCQLYQEHMAYIGEGAKTGIKECQHQFRQRRWNCSTADNASVFGRVMQIGSRETAFTHAV

SAAGVVNAISRACREGELSTCGCSRTARPKDLPRDWLWGGCGDNVEYGYRFAKEFVDARE

REKNFAKGSEEQGRVLMNLQNNEAGRRAVYKMADVACKCHGVSGSCSLKTCWLQLAEFRK

VGDRLKEKYDSAAAMRVTRKGRLELVNSRFTQPTPEDLVYVDPSPDYCLRNESTGSLGTQ

GRLCNKTSEGMDGCELMCCGRGYNQFKSVQVERCHCKFHWCCFVRCKKCTEIVDQYICK

>HUMAN-WNT9A

MLDGSPLARWLAAAFGLTLLLAALRPSAAYFGLTGSEPLTILPLTLEPEAAAQAHYKACD

RLKLERKQRRMCRRDPGVAETLVEAVSMSALECQFQFRFERWNCTLEGRYRASLLKRGFK

ETAFLYAISSAGLTHALAKACSAGRMERCTCDEAPDLENREAWQWGGCGDNLKYSSKFVK

EFLGRRSSKDLRARVDFHNNLVGVKVIKAGVETTCKCHGVSGSCTVRTCWRQLAPFHEVG

KHLKHKYETALKVGSTTNEAAGEAGAISPPRGRASGAGGSDPLPRTPELVHLDDSPSFCL

AGRFSPGTAGRRCHREKNCESICCGRGHNTQSRVVTRPCQCQVRWCCYVECRQCTQREEV

YTCKG

>HUMAN-WNT10A

MGSAHPRPWLRLRPQPQPRPALWVLLFFLLLLAAAMPRSAPNDILDLRLPPEPVLNANTV

CLTLPGLSRRQMEVCVRHPDVAASAIQGIQIAIHECQHQFRDQRWNCSSLETRNKIPYES

PIFSRGFRESAFAYAIAAAGVVHAVSNACALGKLKACGCDASRRGDEEAFRRKLHRLQLD

ALQRGKGLSHGVPEHPALPTASPGLQDSWEWGGCSPDMGFGERFSKDFLDSREPHRDIHA

RMRLHNNRVGRQAVMENMRRKCKCHGTSGSCQLKTCWQVTPEFRTVGALLRSRFHRATLI

RPHNRNGGQLEPGPAGAPSPAPGAPGPRRRASPADLVYFEKSPDFCEREPRLDSAGTVGR

LCNKSSAGSDGCGSMCCGRGHNILRQTRSERCHCRFHWCCFVVCEECRITEWVSVCK

>HUMAN-WNT3

MEPHLLGLLLGLLLGGTRVLAGYPIWWSLALGQQYTSLGSQPLLCGSIPGLVPKQLRFCR

NYIEIMPSVAEGVKLGIQECQHQFRGRRWNCTTIDDSLAIFGPVLDKATRESAFVHAIAS

AGVAFAVTRSCAEGTSTICGCDSHHKGPPGEGWKWGGCSEDADFGVLVSREFADARENRP

DARSAMNKHNNEAGRTTILDHMHLKCKCHGLSGSCEVKTCWWAQPDFRAIGDFLKDKYDS

ASEMVVEKHRESRGWVETLRAKYSLFKPPTERDLVYYENSPNFCEPNPETGSFGTRDRTC

NVTSHGIDGCDLLCCGRGHNTRTEKRKEKCHCIFHWCCYVSCQECIRIYDVHTCK

>HUMAN-WNT2

MNAPLGGIWLWLPLLLTWLTPEVNSSWWYMRATGGSSRVMCDNVPGLVSSQRQLCHRHPD

VMRAISQGVAEWTAECQHQFRQHRWNCNTLDRDHSLFGRVLLRSSRESAFVYAISSAGVV

FAITRACSQGEVKSCSCDPKKMGSAKDSKGIFDWGGCSDNIDYGIKFARAFVDAKERKGK

DARALMNLHNNRAGRKAVKRFLKQECKCHGVSGSCTLRTCWLAMADFRKTGDYLWRKYNG

AIQVVMNQDGTGFTVANERFKKPTKNDLVYFENSPDYCIRDREAGSLGTAGRVCNLTSRG

MDSCEVMCCGRGYDTSHVTRMTKCGCKFHWCCAVRCQDCLEALDVHTCKAPKNADWTTAT

>MOUSE-WNT10A

MGSAHPRPWLRLPQGPQPRPEFWALLFFLLLLAAAVPRSAPNDILGLRLPPEPVLNANTV

CLTLPGLSRRQMEVCVRHPDVAASAIQGIQIAIHECQHQFRDQRWNCSSLETRNKVPYES

PIFSRGFRESAFAYAIAAAGVVHAVSNACALGKLKACGCDASRRGDEEAFRRKLHRLQLD

ALQRGKGLSHGVPEHPAILPASPGLQDSWEWGGCSPDVGFGERFSKDFLDSREPHRDIHA

RMRLHNNRVGRQAVMENMRRKCKCHGTSGSCQLKTCWQVTPEFRTVGALLRNRFHRATLI

RPHNRNGGQLEPGPAGAPSPAPGTPGLRRRASHSDLVYFEKSPDFCEREPRLDSAGTVGR

LCNKSSTGPDGCGSMCCGRGHNILRQTRSERCHCRFHWCCFVVCEECRITEWVSVCK

>MOUSE-WNT5A

MKKPIGILSPGVALGTAGGAMSSKFFLMALATFFSFAQVVIEANSWWSLGMNNPVQMSEV

YIIGAQPLCSQLAGLSQGQKKLCHLYQDHMQYIGEGAKTGIKECQYQFRHRRWNCSTVDN

TSVFGRVMQIGSRETAFTYAVSAAGVVNAMSRACREGELSTCGCSRAARPKDLPRDWLWG

GCGDNIDYGYRFAKEFVDARERERIHAKGSYESARILMNLHNNEAGRRTVYNLADVACKC

HGVSGSCSLKTCWLQLADFRKVGDALKEKYDSAAAMRLNSRGKLVQVNSRFNSPTTQDLV

YIDPSPDYCVRNESTGSLGTQGRLCNKTSEGMDGCELMCCGRGYDQFKTVQTERCHCKFH

WCCYVKCKKCTEIVDQFVCK

>MOUSE-WNT7B

MHRNFRKWIFYVFLCFGVLYVKLGALSSVVALGANIICNKIPGLAPRQRAICQSRPDAII

VIGEGAQMGIDECQHQFRFGRWNCSALGEKTVFGQELRVGSREAAFTYAITAAGVAHAVT

AACSQGNLSNCGCDREKQGYYNQAEGWKWGGCSADVRYGIDFSRRFVDAREIKKNARRLM

NLHNNEAGRKVLEDRMKLECKCHGVSGSCTTKTCWTTLPKFREVGHLLKEKYNAAVQVEV

VRASRLRQPTFLRIKQLRSYQKPMETDLVYIEKSPNYCEEDAATGSVGTQGRLCNRTSPG

ADGCDTMCCGRGYNTHQYTKVWQCNCKFHWCCFVKCNTCSERTEVFTCK

>MOUSE-WNT5B

MPSLLLVVVAALLSSWAQLLTDANSWWSLALNPVQRPEMFIIGAQPVCSQLPGLSPGQRK

LCQLYQEHMSYIGEGAKTGIRECQHQFRQRRWNCSTVDNTSVFGRVMQIGSRETAFTYAV

SAAGVVNAISRACREGELSTCGCSRAARPKDLPRDWLWGGCGDNVEYGYRFAKEFVDARE

REKNFAKGSEEQGRALMNLQNNEAGRRAVYKMADVACKCHGVSGSCSLKTCWLQLAEFRK

VGDRLKEKYDSAAAMRITRQGKLELANSRFNQPTPEDLVYVDPSPDYCLRNETTGSLGTQ

GRLCNKTSEGMDGCELMCCGRGYDRFKSVQVERCHCRFHWCCFVRCKKCTEVVDQYVCK

>MOUSE-WNT1

MGLWALLPSWVSTTLLLALTALPAALAANSSGRWWGIVNIASSTNLLTDSKSLQLVLEPS

LQLLSRKQRRLIRQNPGILHSVSGGLQSAVRECKWQFRNRRWNCPTAPGPHLFGKIVNRG

CRETAFIFAITSAGVTHSVARSCSEGSIESCTCDYRRRGPGGPDWHWGGCSDNIDFGRLF

GREFVDSGEKGRDLRFLMNLHNNEAGRTTVFSEMRQECKCHGMSGSCTVRTCWMRLPTLR

AVGDVLRDRFDGASRVLYGNRGSNRASRAELLRLEPEDPAHKPPSPHDLVYFEKSPNFCT

YSGRLGTAGTAGRACNSSSPALDGCELLCCGRGHRTRTQRVTERCNCTFHWCCHVSCRNC

THTRVLHECL

>MOUSE-WNT7A

MTRKARRCLGHLFLSLGIVYLRIGGFSSVVALGASIICNKIPGLAPRQRAICQSRPDAII

VIGEGSQMGLDECQFQFRNGRWNCSALGERTVFGKELKVGSREAAFTYAIIAAGVAHAIT

AACTQGNLSDCGCDKEKQGQYHRDEGWKWGGCSADIRYGIGFAKVFVDAREIKQNARTLM

NLHNNEAGRKILEENMKLECKCHGVSGSCTTKTCWTTLPQFRELGYVLKDKYNEAVHVEP

VRASRNKRPTFLKIKKPLSYRKPMDTDLVYIEKSPNYCEEDPVTGSVGTQGRACNKTAPQ

ASGCDLMCCGRGYNTHQYARVWQCNCKFHWCCYVKCNTCSERTEMYTCK

>MOUSE-WNT4

MSPRSCLRSLRLLVFAVFSAAASNWLYLAKLSSVGSISEEETCEKLKGLIQRQVQMCKRN

LEVMDSVRRGAQLAIEECQYQFRNRRWNCSTLDSLPVFGKVVTQGTREAAFVYAISSAGV

AFAVTRACSSGELEKCGCDRTVHGVSPQGFQWSGCSDNIAYGVAFSQSFVDVRERSKGAS

SSRALMNLHNNEAGRKAILTHMRVECKCHGVSGSCEVKTCWRAVPPFRQVGHALKEKFDG

ATEVEPRRVGSSRALVPRNAQFKPHTDEDLVYLEPSPDFCEQDIRSGVLGTRGRTCNKTS

KAIDGCELLCCGRGFHTAQVELAERCGCRFHWCCFVKCRQCQRLVEMHTCR

>MOUSE-WNT8A

MGHLLMLWVAAGMCYPALGASAWSVNNFLITRPKAYLTYTASVALGAQIGIEECKFQFAW

ERWNCPEHAFQFSTHNRLRAATRETSFIHAIRSAAIMYAVTKNCSMGDLENCGCDESQNG

KTGGHGWIWGGCSDNVEFGEKISRLFVDSLEKGKDARALVNLHNNRAGRLAVRASTKRTC

KCHGISGSCSIQTCWLQLADFRQMGNYLKAKYDRALKIEMDKRQLRAGNRAEGRWALTEA

FLPSTEAELIFLEGSPDYCNRNASLSIQGTEGRECLQNARSASRREQRSCGRLCTECGLQ

VEERRAEAVSSCDCNFQWCCTVKCGQCRRVVSRYYCTRPVGSARPRGRGKDSAW

>MOUSE-WNT2

MNVPLGGIWLWLPLLLTWLTPEVSSSWWYMRATGGSSRVMCDNVPGLVSRQRQLCHRHPD

VMRAIGLGVAEWTAECQHQFRQHRWNCNTLDRDHSLFGRVLLRSSRESAFVYAISSAGVV

FAITRACSQGELKSCSCDPKKKGSAKDSKGTFDWGGCSDNIDYGIKFARAFVDAKERKGK

DARALMNLHNNRAGRKAVKRFLKQECKCHGVSGSCTLRTCWLAMADFRKTGDYLWRKYNG

AIQVVMNQDGTGFTVANKRFKKPTKNDLVYFENSPDYCIRDREAGSLGTAGRVCNLTSRG

MDSCEVMCCGRGYDTSHVTRMTKCECKFHWCCAVRCQDCLEALDVHTCKAPKSADWATPT

>MOUSE-WNT6

MLPPVPSRLGLLLLLLCPAHVDGLWWAVGSPLVMDPTSICRKARRLAGRQAELCQAEPEV

VAELARGARLGVRECQFQFRFRRWNCSSHSKAFGRVLQQDIRETAFVFAITAAGASHAVT

QACSMGELLQCGCQAPRGRAPPRPSGLLGTPGPPGPTGSPDASAAWEWGGCGDDVDFGDE

KSRLFMDAQHKRGRGDIRALVQLHNNEAGRLAVRSHTRTECKCHGLSGSCALRTCWQKLP

PFREVGARLLERFHGASRVMGTNDGKALLPAVRTLKPPGRADLLYAADSPDFCAPNRRTG

SPGTRGRACNSSAPDLSGCDLLCCGRGHRQESVQLEENCLCRFHWCCVVQCHRCRVRKEL

SLCL

>MOUSE-WNT3

MEPHLLGLLLGLLLSGTRVLAGYPIWWSLALGQQYTSLASQPLLCGSIPGLVPKQLRFCR

NYIEIMPSVAEGVKLGIQECQHQFRGRRWNCTTIDDSLAIFGPVLDKATRESAFVHAIAS

AGVAFAVTRSCAEGTSTICGCDSHHKGPPGEGWKWGGCSEDADFGVLVSREFADARENRP

DARSAMNKHNNEAGRTTILDHMHLKCKCHGLSGSCEVKTCWWAQPDFRAIGDFLKDKYDS

ASEMVVEKHRESRGWVETLRAKYALFKPPTERDLVYYENSPNFCEPNPETGSFGTRDRTC

NVTSHGIDGCDLLCCGRGHNTRTEKRKEKCHCVFHWCCYVSCQECIRIYDVHTCK

>MOUSE-WNT9A

MLDGSLLARWLAAAFGLTLLLAALRPSAAYFGLTGSEPLTILPLTLETEAAAQAHYKACD

RLKLERKQRRMCRRDPGVAETLVEAVSMSALECQYQFRFERWNCTLEGRYRASLLKRGFK

ETAFLYAISSAGLTHALAKACSAGRMERCTCDEAPDLENREAWQWGGCGDNLKYSSKFVK

EFLGRRSSKDLRARVDFHNNLVGVKVIKAGVETTCKCHGVSGSCTVRTCWRQLAPFHEVG

KHLKHKYETSLKVGSTTNEATGEAGAISPPRGRASGSGGGDPLPRTPELVHLDDSPSFCL

AGRFSPGTAGRRCHREKNCESICCGRGHNTQSRVVTRPCQCQVRWCCYVECRQCTQREEV

YTCKG

>MOUSE-WNT2B

MLKLQGEDEAAQLAPRRARVPVPRPTAPDVSPSSARLGLACLLLLLLLTLPARVDTSWWY

IGALGARVICDNIPGLVSRQRQLCQRYPDIMRSVGEGAREWIRECQHQFRHHRWNCTTLD

RDHTVFGRAMLRSSREAAFVYAISSAGVVHAITRACSQGELSVCSCDPYTRGRHHDQRGD

FDWGGCSDNIHYGVRFAKAFVDAKEKRLKDARALMNLHNNRCGRTAVRRFLKLECKCHGV

SGSCTLRTCWRALSDFRRTGDYLRRRYDGAVQVTATQDGANFTAARQGYRHATRTDLVYF

DNSPDYCVLDKAAGSLGTAGRVCSKTSKGTDGCEIMCCGRGYDTTRVTRVTQCECKFHWC

CAVRCKECRNTVDVHTCKAPKKAEWLDQT

>MOUSE-WNT10B

MLEEPRSRPPPLGLAGLLFLALFSRALSNEILGLKLPGEPPLTANTVCLTLSGLSKRQLG

LCLRSPDVTASALQGLHIAVHECQHQLRDQRWNCSALEGGGRLPHHSAILKRGFRESAFS

FSMLAAGVMHAVATACSLGKLVSCGCGWKGSGEQDRLRAKLLQLQALSRGKTFPISQPSP

VPGSVPSPGPQDTWEWGGCNHDMDFGEKFSRDFLDSREAPRDIQARMRIHNNRVGRQVVT

ENLKRKCKCHGTSGSCQFKTCWRAAPEFRAIGAALRERLSRAIFIDTHNRNSGAFQPRLR

PRRLSGELVYFEKSPDFCERDPTLGSPGTRGRACNKTSRLLDGCGSLCCGRGHNVLRQTR

VERCHCRFHWCCYVLCDECKVTEWVNVCK

>MOUSE-WNT11

MRARPQVCEALLFALALHTGVCYGIKWLALSKTPAALALNQTQHCKQLEGLVSAQVQLCR

SNLELMRTIVHAARGAMKACRRAFADMRWNCSSIELAPNYLLDLERGTRESAFVYALSAA

TISHTIARACTSGDLPGCSCGPVPGEPPGPGNRWGGCADNLSYGLLMGAKFSDAPMKVKK

TGSQANKLMRLHNSEVGRQALRASLETKCKCHGVSGSCSIRTCWKGLQELQDVAADLKTR

YLSATKVVHRPMGTRKHLVPKDLDIRPVKDSELVYLQSSPDFCMKNEKVGSHGTQDRQCN

KTSNGSDSCDLMCCGRGYNPYTDRVVERCHCKYHWCCYVTCRRCERTVERYVCK

>MOUSE-WNT9B

MRPAPALALAALCLLVLPAAAAAAAYFGLTGREVLTPFPGLGTAAAPAQAGAHLKQCDLL

KLSRRQKQLCRREPGLAETLRDAAHLGLLECQFQFRQERWNCSLEGRTGLLQRGFKETAF

LYAVSAAALTHALARACSAGRMERCTCDDSPGLESRQAWQWGVCGDNLKYSTKFLSNFLG

PKRGSKDLRARADAHNTHVGIKAVKSGLRTTCKCHGVSGSCAVRTCWKQLSPFRETGQVL

KLRYDTAVKVSSATNEALGRLELWAPAKPGGPAKGLAPRPGDLVYMEDSPSFCRPSKYSP

GTAGRVCSRDSSCSSLCCGRGYDTQSRMVVFSCHCQVQWCCYVECQQCAQQELVYTCKR

>MOUSE-WNT3A

MAPLGYLLVLCSLKQALGSYPIWWSLAVGPQYSSLSTQPILCASIPGLVPKQLRFCRNYV

EIMPSVAEGVKAGIQECQHQFRGRRWNCTTVSNSLAIFGPVLDKATRESAFVHAIASAGV

AFAVTRSCAEGSAAICGCSSRLQGSPGEGWKWGGCSEDIEFGGMVSREFADARENRPDAR

SAMNRHNNEAGRQAIASHMHLKCKCHGLSGSCEVKTCWWSQPDFRTIGDFLKDKYDSASE

MVVEKHRESRGWVETLRPRYTYFKVPTERDLVYYEASPNFCEPNPETGSFGTRDRTCNVS

SHGIDGCDLLCCGRGHNARTERRREKCHCVFHWCCYVSCQECTRVYDVHTCK

>MOUSE-WNT8B

MFLMKPVCVLLVTCVLHRSHAWSVNNFLMTGPKAYLVYSSSVAAGAQSGIEECKYQFAWD

RWNCPERALQLSSHGGLRSANRETAFVHAISSAGVMYTLTRNCSLGDFDNCGCDDSRNGQ

LGGQGWLWGGCSDNVGFGEAISKQFVDALETGQDARAAMNLHNNEAGRKAVKGTMKRTCK

CHGVSGSCTTQTCWLQLPEFREVGAHLKEKYHAALKVDLLQGAGNSAAGRGAIADTFRSI

STRELVHLEDSPDYCLENKTLGLLGTEGRECLRRGRALGRWERRSCRRLCGDCGLAVEER

RAETVSSCNCKFHWCCAVRCEQCRRRVTKYFCSRAERPPRGAAHKPGKNS

>MOUSE-WNT16

MDRAALLALPSLCALWAAVLSLLPCGTQGNWMWLGIASFGVPEKLGCADLPLNSRQKELC

KRKPYLLPSIREGARLGIQECRSQFRHERWNCMVATTTSTQLATAPLFGYELSSGTKETA

FIYAIMAAGLVHSVTRSCSAGNMTECSCDTTLQNGGSPSEGWHWGGCSDDVQYGMWFSRK

FLDLPIRNTTGKESRVLLAMNLHNNEAGRQAVAKLMSVDCRCHGVSGSCAVKTCWKTMSS

FEKIGHFLKDKYENSIQISDKTKRKMRRREKDQRQTPILKDDLLYVHKSPNYCVENKKLG

IPGTQGRECNRTSGGADGCNLLCCGRGYNTHVVRHVERCECKFIWCCYVRCRRCESMTDV

HTCK
