# Supplementary material for: Genome-wide identification and expression profiling analysis of Wnt family genes affecting adipocyte differentiation in cattle
Source: Sci Rep. 2022 Jan 11;12:489. doi: 10.1038/s41598-021-04468-1 (PMC8752766; doi:10.1038/s41598-021-04468-1)
Supplement: Supplementary file 3 — Supplementary Information 3. [file 41598_2021_4468_MOESM3_ESM.pdf]

a

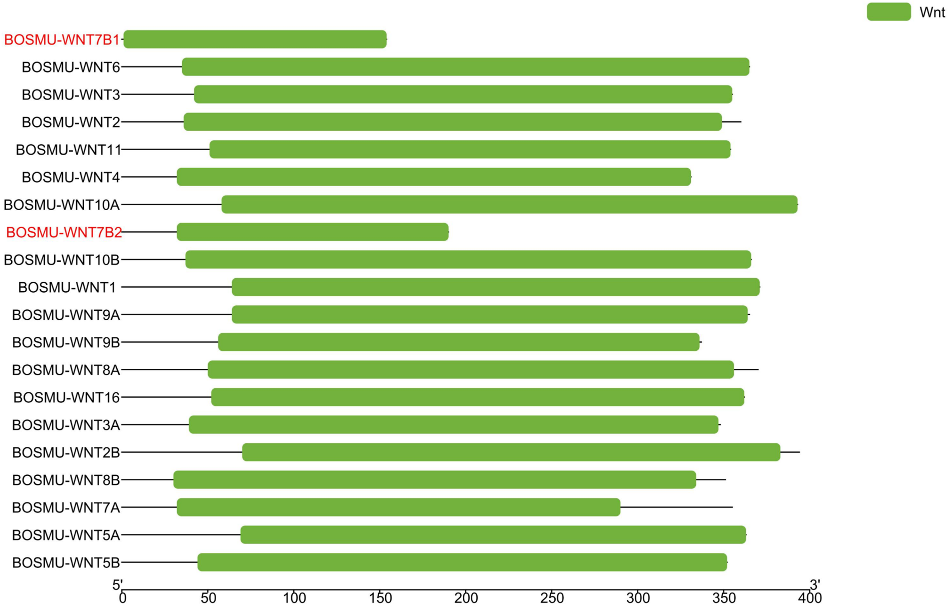

Conserved domain prediction of *Bos mutus* Wnt proteins

b

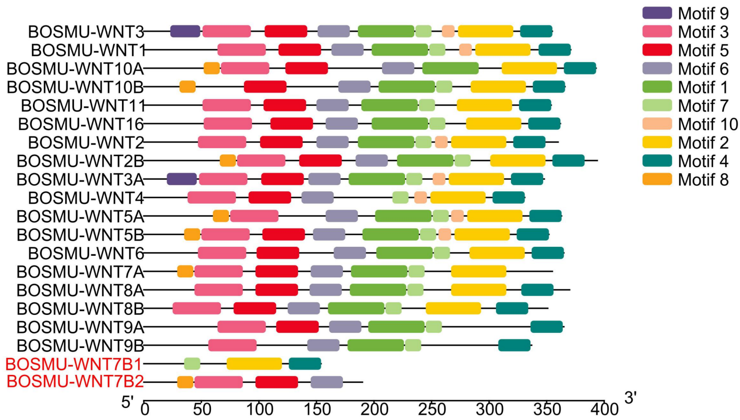

Conserved motifs prediction of *Bos mutus* Wnt proteins
