# Supplementary figures and images for: Genome-wide identification and expression profiling analysis of Wnt family genes affecting adipocyte differentiation in cattle
Source: Sci Rep. 2022 Jan 11;12:489. doi: 10.1038/s41598-021-04468-1 (PMC8752766; doi:10.1038/s41598-021-04468-1)

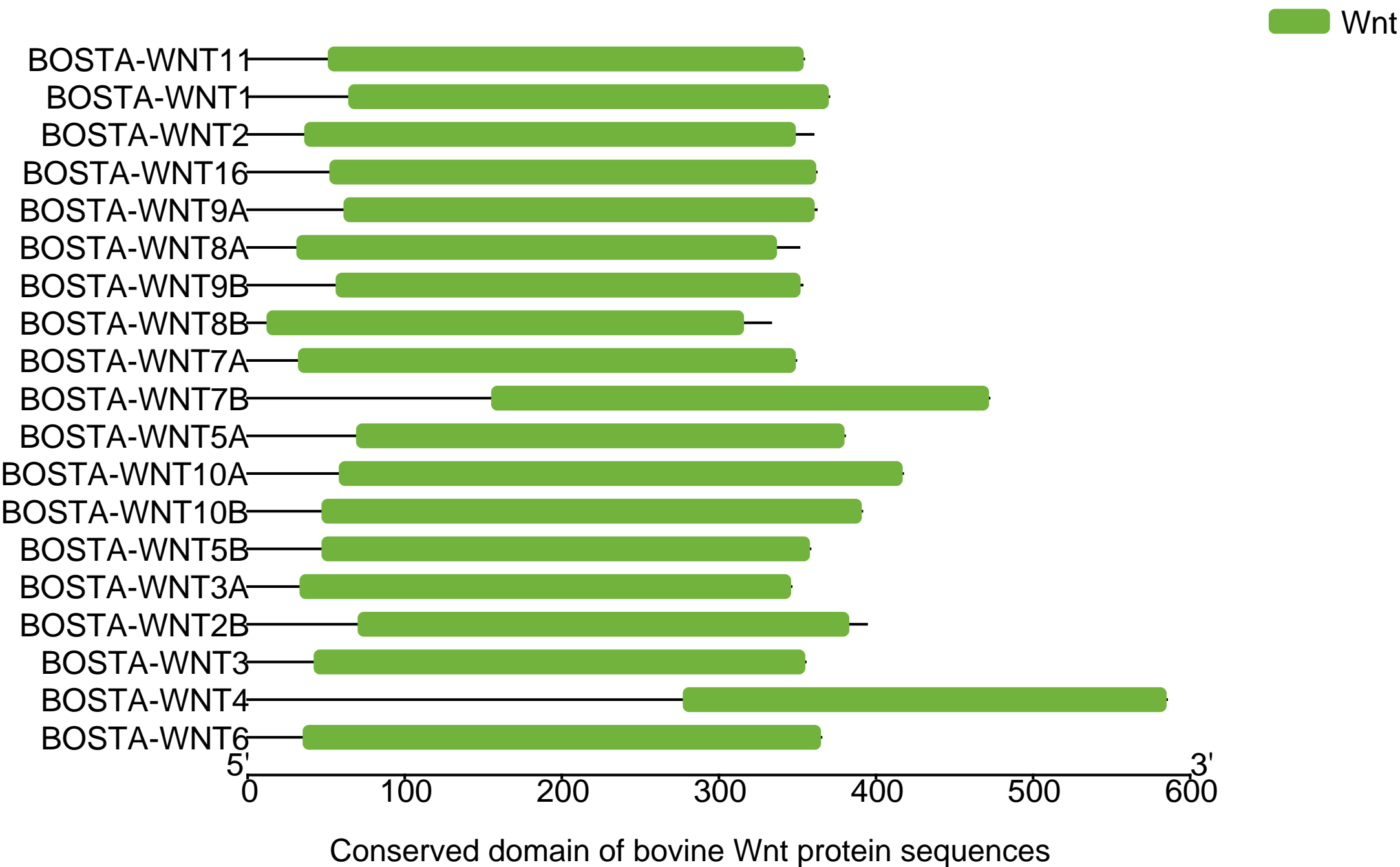

Supplement: Supplementary file 4 — Supplementary Information 4. [file 41598_2021_4468_MOESM4_ESM.pdf]

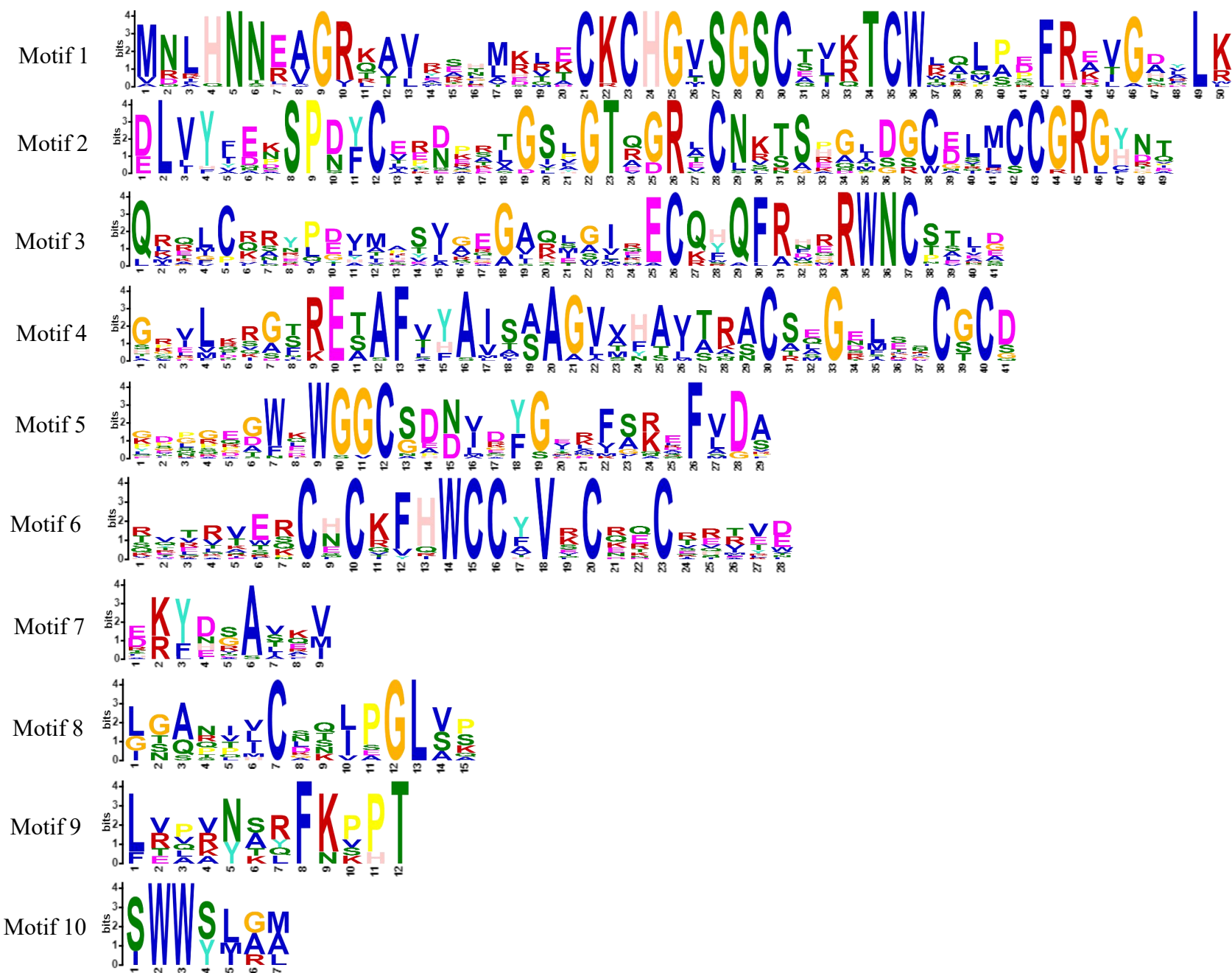

Amino acid sequence

Supplement: Supplementary file 5 — Supplementary Information 5. [file 41598_2021_4468_MOESM5_ESM.pdf]

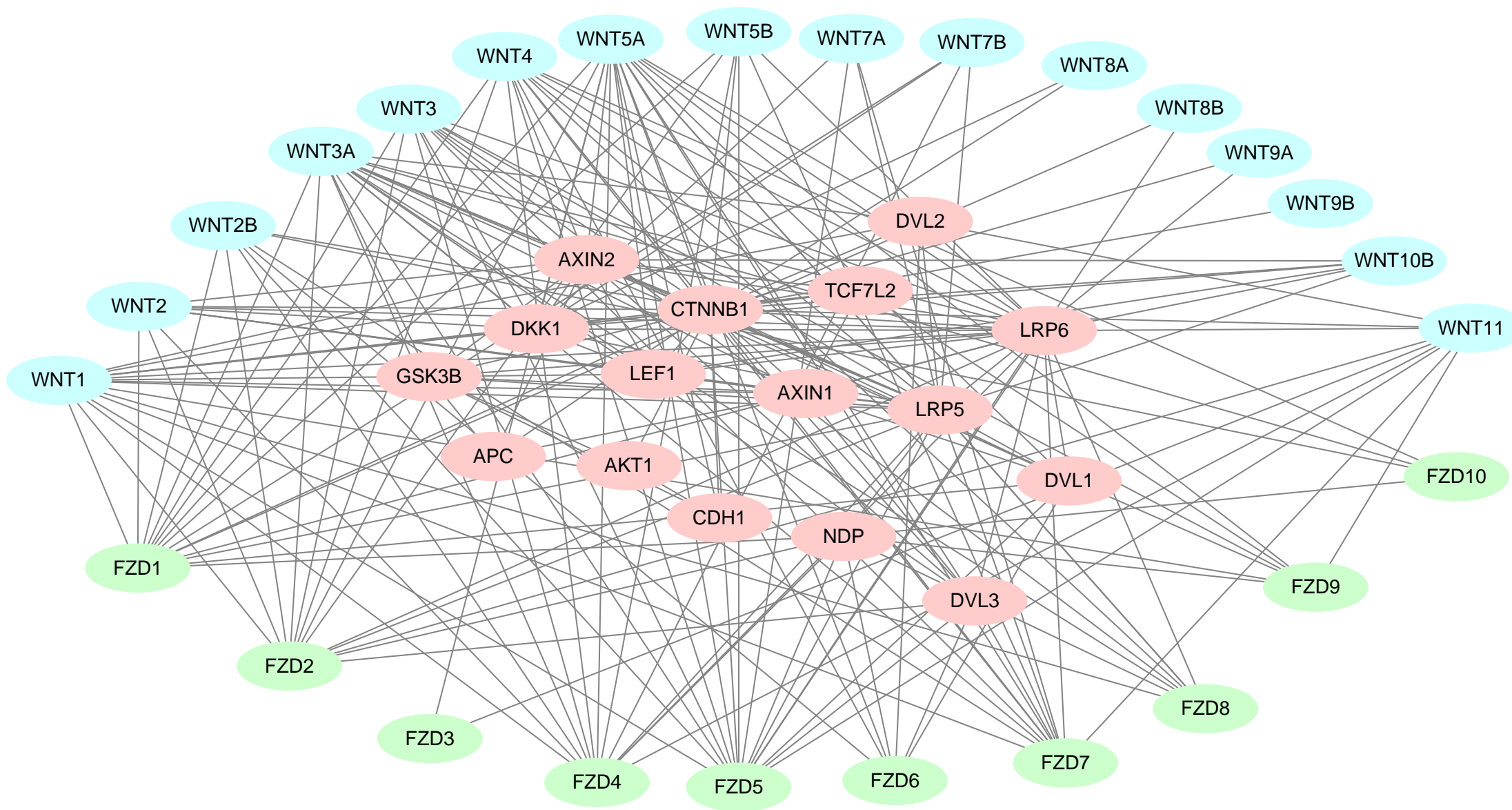

The interaction network for *Wnt* and its related genes

Supplement: Supplementary file 7 — Supplementary Information 7. [file 41598_2021_4468_MOESM7_ESM.pdf]
