# Supplementary material for: Genome-wide identification and expression profiling analysis of Wnt family genes affecting adipocyte differentiation in cattle
Source: Sci Rep. 2022 Jan 11;12:489. doi: 10.1038/s41598-021-04468-1 (PMC8752766; doi:10.1038/s41598-021-04468-1)
Supplement: Supplementary file 6 — Supplementary Information 6. [file 41598_2021_4468_MOESM6_ESM.pdf]

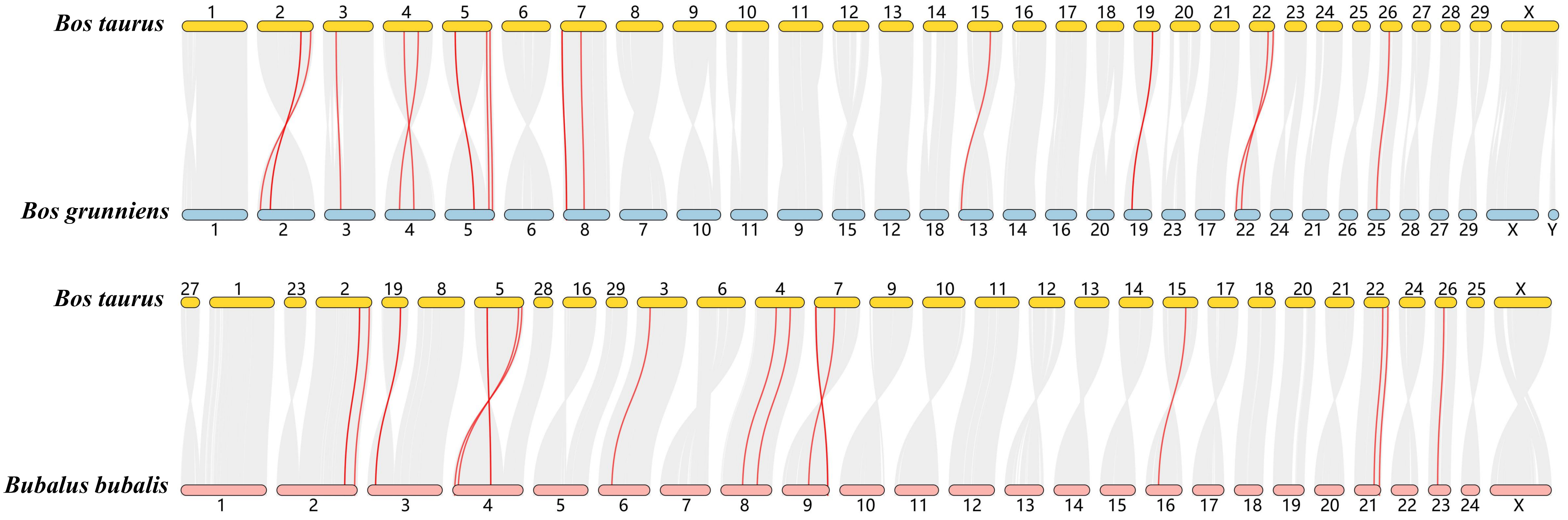

The adjusted collinearity relationship between *Bos taurus* and *Bos grunniens* and *Bubalus bubalis*
